# Plasma Metabolic Signatures of Healthy Overweight Subjects Challenged With an Oral Glucose Tolerance Test

**DOI:** 10.3389/fnut.2022.898782

**Published:** 2022-06-14

**Authors:** Jarlei Fiamoncini, Carlos M. Donado-Pestana, Graziela Biude Silva Duarte, Milena Rundle, Elizabeth Louise Thomas, Yoana Kiselova-Kaneva, Thomas E. Gundersen, Diana Bunzel, Jean-Pierre Trezzi, Sabine E. Kulling, Karsten Hiller, Denise Sonntag, Diana Ivanova, Lorraine Brennan, Suzan Wopereis, Ben van Ommen, Gary Frost, Jimmy Bell, Christian A. Drevon, Hannelore Daniel

**Affiliations:** ^1^Department Food and Nutrition, Technische Universität München, Freising, Germany; ^2^Food Research Center, Department of Food Science and Experimental Nutrition, School of Pharmaceutical Sciences, University of São Paulo, São Paulo, Brazil; ^3^Division of Diabetes, Endocrinology and Metabolism, Department of Medicine, Imperial College London, London, United Kingdom; ^4^Research Centre for Optimal Health, School of Life Sciences, University of Westminster, London, United Kingdom; ^5^Department of Biochemistry, Molecular Medicine and Nutrigenomics, Medical University, Varna, Bulgaria; ^6^Vitas Ltd., Oslo Science Park, Oslo, Norway; ^7^Department of Safety and Quality of Fruit and Vegetables, Federal Research Institute of Nutrition and Food, Max Rubner-Institut, Karlsruhe, Germany; ^8^Braunschweig Integrated Centre of Systems Biology, University of Braunschweig, Braunschweig, Germany; ^9^Department of Computational Biology of Infection Research, Helmholtz Centre for Infection Research, Braunschweig, Germany; ^10^biocrates life sciences AG, Innsbruck, Austria; ^11^UCD School of Agriculture and Food Science, Institute of Food and Health, Conway Institute, University College Dublin, Dublin, Ireland; ^12^Netherlands Organisation for Applied Scientific Research, Netherlands Institute for Applied Scientific Research, Microbiology and Systems Biology, Zeist, Netherlands; ^13^Department of Nutrition, Faculty of Medicine, Institute of Basic Medical Sciences, University of Oslo, Oslo, Norway

**Keywords:** dietary challenge test, metabotypes, OGTT, postprandial metabolism, insulin resistance

## Abstract

Insulin secretion following ingestion of a carbohydrate load affects a multitude of metabolic pathways that simultaneously change direction and quantity of interorgan fluxes of sugars, lipids and amino acids. In the present study, we aimed at identifying markers associated with differential responses to an OGTT a population of healthy adults. By use of three metabolite profiling platforms, we assessed these postprandial responses of a total of 202 metabolites in plasma of 72 healthy volunteers undergoing comprehensive phenotyping and of which half enrolled into a weight-loss program over a three-month period. A standard oral glucose tolerance test (OGTT) served as dietary challenge test to identify changes in postprandial metabolite profiles. Despite classified as healthy according to WHO criteria, two discrete clusters (A and B) were identified based on the postprandial glucose profiles with a balanced distribution of volunteers based on gender and other measures. Cluster A individuals displayed 26% higher postprandial glucose levels, delayed glucose clearance and increased fasting plasma concentrations of more than 20 known biomarkers of insulin resistance and diabetes previously identified in large cohort studies. The volunteers identified by canonical postprandial responses that form cluster A may be called pre-pre-diabetics and defined as “at risk” for development of insulin resistance. Moreover, postprandial changes in selected fatty acids and complex lipids, bile acids, amino acids, acylcarnitines and sugars like mannose revealed marked differences in the responses seen in cluster A and cluster B individuals that sustained over the entire challenge test period of 240 min. Almost all metabolites, including glucose and insulin, returned to baseline values at the end of the test (at 240 min), except a variety of amino acids and here those that have been linked to diabetes development. Analysis of the corresponding metabolite profile in a fasting blood sample may therefore allow for early identification of these subjects at risk for insulin resistance without the need to undergo an OGTT.

## Introduction

Subjects with insulin resistance display impaired phenotypic flexibility as a consequence of a reduced capacity for insulin-stimulated glucose uptake into muscle and adipose tissue and insufficient suppression of hepatic gluconeogenesis (1, 2). The oral glucose tolerance test (OGTT) is an effective assessment method for identification of an insulin resistant state, a pre-stage for multiple chronic diseases, including diabetes type II. Insulin by its pleiotropic actions affects interorgan fluxes of almost all nutrient classes and in turn, insulin resistance results in impaired metabolite partitioning and that may therefore contribute to metabolic dysregulation (3, 4).

The application of metabolic profiling attempts in human studies are often limited to biosamples that can be easily collected such as urine or plasma/serum. Yet, these metabolite patterns can provide insights into physiological control processes or the establishment of diseases (5, 6). Metabolic profiling techniques applied in human studies have led to the identification of plasma metabolite signatures associated with insulin resistance dominated by branched-chain amino acids (BCAA), acylcarnitines, gluconeogenesis precursors, bile acids (BA), ketone bodies and specific lipid groups (7–9). When applied together with a dietary challenge such as the OGTT these techniques may identify adaptive response patterns as well as biomarkers and pathways that describe the flexibility of the metabolic system or its impairment and role in the development of non-communicable chronic diseases (6, 10). Following digestion of food and absorption of nutrients, individual organs are provided with these substrates in processes mostly regulated by insulin. These processes also reveal a high individual variability with the term “metabotype” introduced to describe the different patterns of metabolic response or different “metabolic phenotypes” (11, 12). The characterization and classification may contribute to the development of new interventions in the framework of personalized nutrition (13). Unfortunately, most human studies that employ metabolite profiling technologies, assess subjects in the overnight fasting condition only, therefore lacking phenotypical information derived from the response to a meal. This creates a significant gap of information as humans with safe access to food spend most of their waking hours in the postprandial state.

We previously identified distinct metabotypes within a healthy study population, identified based on their response to a mixed meal, revealing a co-regulation of different physiologic processes, allowing early detection of metabolic impairments and susceptibility to the beneficial effects of energy restriction (14). In the present study, we aimed at identifying markers associated with differential responses to an OGTT in the same population of healthy adults. In this exercise, we describe the close association of several metabolites in plasma with the insulin-dependent postprandial responses employing 3 metabolite profiling platforms. The analysis enabled the identification of new biomarkers to be identified that are linked to the body's insulin-responsiveness.

## Materials and Methods

### Ethics Approval and Study Registration

The intervention study was conducted under the umbrella of the “NutriTech” project and was carried out at NIHR/Wellcome Trust Imperial Clinical Research Facility at Hammersmith Hospital of Imperial College London. The study was approved by the Brent Ethics Committee (REC ref: 12/LO/0139) and registered at clinicaltrials.gov record: NCT01684917. NutriTech was funded by the European Union Framework 7 program.

### Study Population and Experimental Design

The data presented here was obtained from one out of the three dietary challenges (OGTT) performed in the NutriTech study. Participants attended a health-screening visit at the research facility that included measurements of height, weight, body composition by bioelectric impedance, blood pressure, electrocardiogram and markers of clinical chemistry: glucose, insulin, glycated hemoglobin, plasma lipids, hematocrit, liver and kidney functions. A total of 72 subjects (38 women and 34 men) that displayed no signs of metabolic diseases judged by the clinical assessment during screening completed the study. All female participants were post-menopausal. The individuals were overweight/ obese, with BMI ranging from 24.7 to 35.5.

All volunteers underwent an oral glucose tolerance test (OGTT). Subjects were instructed to avoid alcohol consumption and strenuous exercise prior to each study visit. The OGTT started at 09:00 am following a 12-h fasting. Upon arrival, participants had a catheter placed in the antecubital vein by a trained nurse and a fasting blood sample was obtained. The test consisted in the ingestion of 75 g of glucose dissolved in 250 mL of water and blood sampling at 0, 15, 30, 60, 90, 120 and 240 min. The cannula was flushed with saline between blood collections and 3 mL of waste was drawn first to allow for the saline diluted blood before the blood was taken for analysis. Plasma (from heparin coated tubes) and serum were separated after centrifugation at 1,800 rpm and stored at −80°C for future analyses.

### Measurement of Markers of Intermediate Metabolism and Inflammation

Serum insulin concentration was measured by radioimmunoassay using a Human Specific Insulin RIA Kit (Millipore Corporation) accordingly to manufacturer's instructions. Glucose levels in serum were measured by an enzymatic method using an Abbott Architect ci8200 analyzer. Plasma glucagon was measured with a RIA kit (GL-32K Sigma-Aldrich). PYY and GLP-1 were also measured using RIA, following previously established methods (15, 16). Non-esterified fatty acids (NEFA), albumin, ammonia, urea, creatine, aspartate aminotransferase, gamma-glutamyl transpeptidase, total cholesterol, HDL-cholesterol, LDL-cholesterol, triacylglycerides, and uric acid levels, where measured using standard enzymatic methodology according to manufacturer's instructions. Leptin levels in plasma were assayed by a sandwich enzyme immunoassay (ELISA) (BioVendor, Czech Republic). The cytokines and other inflammation markers like CRP, IL-8, IL-10-, IL-18, ICAM-1, MCP-1 were measured by Vitas AS (www.vitas.no) using ELISA technology. IL-8, ICAM-1, IL-10, sE-selectin, TNF-α, CRP, adiponectin and IL-1β in plasma were analyzed using ELISA kits (Invitrogen Corporation, USA). MCP-1 and sVCAM-1 in plasma were analyzed using ELISA kits (Life Technologies, USA).

Plasma fatty acids (FA) were measured by 2 independent groups with different methodologies. One of the groups (Vitas AS, Oslo, Norway) quantified FA in the fasting state using GC-FID providing data expressed as μg/mL and a FA profile expressed in percentage (17), while the other group analyzed FA concentrations in plasma sampled during the OGTT using GC-MS.

### Assessment of Body Composition and Physical Activity

Adipose tissue content and distribution, as well as liver and muscle fat content were assessed using magnetic resonance imaging (MRI) and spectroscopy (MRS), on a 1.5T Phillips multinuclear system as previously described (18). Briefly, single voxel spectra (2 x 2 x 2 cm^3^; TE/TR= 135/1500 ms) were obtained from the liver using a PRESS sequence for measurement of intrahepatocellular lipid (IHCL). Spectra were also acquired from the Soleus and Tibialis muscles to measure intra-myocellular lipid (IMCL). Body fat was assessed using a whole-body rapid T1-weighted spin echo sequence. Participants were scanned from head to toes by acquiring 10 mm thick transverse images with 10 mm gaps between slices.

In order to assess the level of physical activity, participants were asked to wear an accelerometer (BodyMedia SenseWear, USA) on the non-dominant arm for 7 days. Final value was presented as day average in metabolic equivalents (METs).

### Mass Spectrometry-Based Plasma Metabolite Profiling

All plasma samples were randomized to exclude batch variation. Quality control plasma samples (Recipe chemicals and instruments, Munich, Germany) were included into each set of samples to control for instrument drifting and other technical issues during measurements.

Acylcarnitines (19) amino acids (20), biogenic amines (12), glycerophospholipids (90), and sphingolipids (15) were quantified in plasma using the LC-MS/MS based AbsoluteIDQ® p180 Kit (biocrates life sciences AG, Innsbruck, Austria), following the manufacturer's protocol and excluding metabolites below the limit of detection. Additional acylcarnitines (21) were quantified after sample extraction with methanol in the presence of deuterated standards and butylated prior to analysis using LC-MS/MS coupled to a Sciex 5500 MS (Sciex, USA) following a previously described method (22). The 13 most abundant bile acids in plasma were quantified using an adaptation of the method previously described. Briefly, 10 μL of plasma were mixed with deuterated internal standards and after methanolic extraction, the samples were evaporated to dryness, reconstituted in methanol:water (1:1) and injected into the LC-MS/MS system as described (23).

For the GC-MS analysis, metabolites were extracted from 40 μL plasma aliquots using ice-cold methanol:H_2_O (8:1) in a ratio of 1:10 (sample:solvent). After centrifugation (13,200 g, 4 min, 4°C), 200 μL of supernatant was completely dried under vacuum. A 2-step derivatization was performed using an autosampler (Agilent 7693, Agilent Technologies, Germany) by incubating the samples with methoxyamine hydrochloride (20 mg/mL in pyridine) for 30 min at 45°C, followed by the addition of N-methyl-N-trimethylsilyl-triflouroacetamide, and a second incubation for 30 min at 45°C. Each sample was thereafter immediately submitted to GC–MS analysis (Agilent 6890N GC coupled to an Agilent 5975C inert XL - Agilent Technologies, Germany). The gas chromatograph was equipped with a 30 m DB-35MS capillary column (Agilent J&W GC Column). Metabolites were eluted by a temperature gradient starting at 80°C and rising by 11°C/min to 325°C with 5 min hold at 325°C. Metabolite identification and quantification was accomplished using the Metabolite Detector software. Metabolites were identified according to their retention time and spectra similarity against the Golm metabolome database.

### Data Analysis

The aim of this study was to assess metabolic differences in subjects that display differential glucose responses to an OGTT. Our first approach was to check for different patterns of glycemic response to the OGTT using a hierarchical cluster analysis (HCA) based on glucose concentrations measured at 7 time points during the test. The analysis identified 2 clear clusters of subjects or patterns of response (Supplementary Figure 1). Cluster A was comprised of 33 subjects of whom 14 were female (42%), whereas cluster B had 39 subjects of whom 24 were females (61% female). A Chi-square test ruled out the gender discrepancy between the two groups (*p* = 0.10, Table 1).

**Table 1:** Characteristics of the study population.

	**A**			**B**			***t* test**
	**Mean**	**SD**	**N**	**Mean**	**SD**	**N**	***p*-Value**
Age (years)	60.2	3.26	33	58.3	4.66	39	0.063
BMI (kg/m^2^)	29.39	3.02	33	29.01	2.53	39	0.565
TAT (kg)	31.52	10.20	29	30.97	7.93	39	0.806
TAT (%BW)	36.00	9.32	29	37.92	9.05	39	0.395
DBP (mmHg)	77.89	6.76	33	77.31	10.14	39	0.778
SBP (mmHg)	126.64	10.38	33	126.74	14.08	39	0.971
Fasting glucose (mmol/L)	5.58	0.42	33	4.81	0.39	39	<0.000001
Fasting insulin (mIU/L)	19.09	8.42	31	13.12	4.13	39	0.0002
HOMA-IR	4.53	1.79	30	2.81	0.90	39	0.000002
Cholesterol (mmol/L)	4.83	0.93	32	4.90	0.83	37	0.736
HDL-chol (mmol/L)	1.62	0.15	32	1.69	0.20	37	0.088
LDL-chol (mmol/L)	2.53	0.86	32	2.69	0.77	37	0.414
GGTP (U/L)	28.72	21.79	32	22.22	22.08	37	0.224
AST (U/L)	20.84	7.06	32	21.76	9.60	37	0.659
AP (IU/L)	77.44	20.44	32	72.32	23.18	37	0.338
Uric acid (μmol/L)	319.94	90.28	32	286.32	72.04	37	0.090
TSH (mUl/L)	1.68	0.76	32	1.81	0.96	39	0.546
T4 (pmol/L)	13.47	1.46	32	13.23	1.37	39	0.481
Leptin (ng/mL)	15.72	8.32	32	14.53	8.02	38	0.547
TNF-α (pg/mL)	3.31	1.12	33	3.57	2.08	39	0.526
IL-18 (pg/mL)	266.42	88.40	33	235.82	105.30	39	0.191
VCAM (ng/mL)	1,909.76	980.45	33	1,674.46	709.51	39	0.243
CRP (ng/mL)	674.61	709.68	33	1,198.10	2,529.50	39	0.254
Female (N)	14 (42%)			24 (61%)			X2 *p-*value = 0.10

*As a recruitment criterion, participants of the study were considered healthy after a medical assessment. Data presented as mean ± standard devdiation. The bottom row provides the gender distribution in each cluster, which was found not to be different, according to a Chi square test (p = 0.10). AST, aspartate aminotransferase; AP, alkaline phosphatase; BMI, body mass index; CRP, C-reactive protein; DBP, diastolic blood pressure; GGTP, gamma-glutamyl transpeptidase; HDL, high density lipoproteins; HOMA-IR, Homeostatic model assessment for insulin resistance; IL-18, interleukin 18; LDL, low density lipoproteins; SBP, systolic blood pressure; TAT, total adipose tissue; TNF-a, tumor necrosis factor-alpha; TSH, thyroid-stimulating hormone; T4, thyroxine; VCAM, vascular cell adhesion molecule*.

A partial least squares discriminant analysis (PLS-DA) was performed including all measured variables, except for glucose and insulin to compare clusters A and B and identify discriminant variables. A list with all metabolites and other variables used in the analysis is presented in Supplementary Table 1. The model was built with 1,222 variables classified into the following 14 categories: bile acids, acylcarnitines, oxidative stress markers, amino acid metabolism markers, signaling molecules, biogenic amines, body composition, lipid metabolism markers, inflammation, leucocytes, markers of glucose metabolism, glycerophospholipids and sphingomyelins (Supplementary Table 1). In case of variables measured in all 7 plasma samples collected during the OGTT, each data point was considered as an independent variable. The variables with Variable Importance in the Projection (VIP) value ≥ 1 were further analyzed in univariate statistical analysis, except when only one data point of a given metabolite measured during the OGTT received a VIP value >1. In these cases, the metabolite was not considered of relevance and was not further analyzed. The selected variables are presented as line graphs, if considered different between the clusters after a mixed-effects analysis. Additionally, a Pearson's correlation analysis was performed between all selected variables that were statistically different between clusters A and B with the average glucose concentration during the OGTT (postprandial glycaemia).

Depending on the nature of comparisons, ordinary one-way ANOVA or a mixed-effects analysis (in case of variables measured in samples collected during the OGTT) were used to test differences between the groups. Multiple comparisons were tested using Tukey's *post-hoc* test. Differences with *p* < 0.05 were considered as significant. Shapiro-Wilk test was used to assess normality (*p* < 0.05). In cases where outliers were removed, their detection was done using the ROUT method. Pearson's multiple correlation analysis was performed assuming that most of the variables followed a normal distribution. GraphPad Prism Software (version 9.0) was used for these analyses and preparing most of the figures. PLS-DA was performed using SIMCA software (version 16) after data scaling using unit variance.

## Results

The study population was considered overweight/obese according to the WHO (average BMI = 29.2), but did not present high blood pressure nor hyperlipidemia, despite the mean age of 59.2 years old. The presence of obese individuals (BMI > 29.9) was proportional in both groups: 45% in group A and 36% in group B. There were also no differences in the plasma concentrations of thyroid hormones, leptin or inflammatory markers between clusters A and B (Table 1).

**Figure 1:**
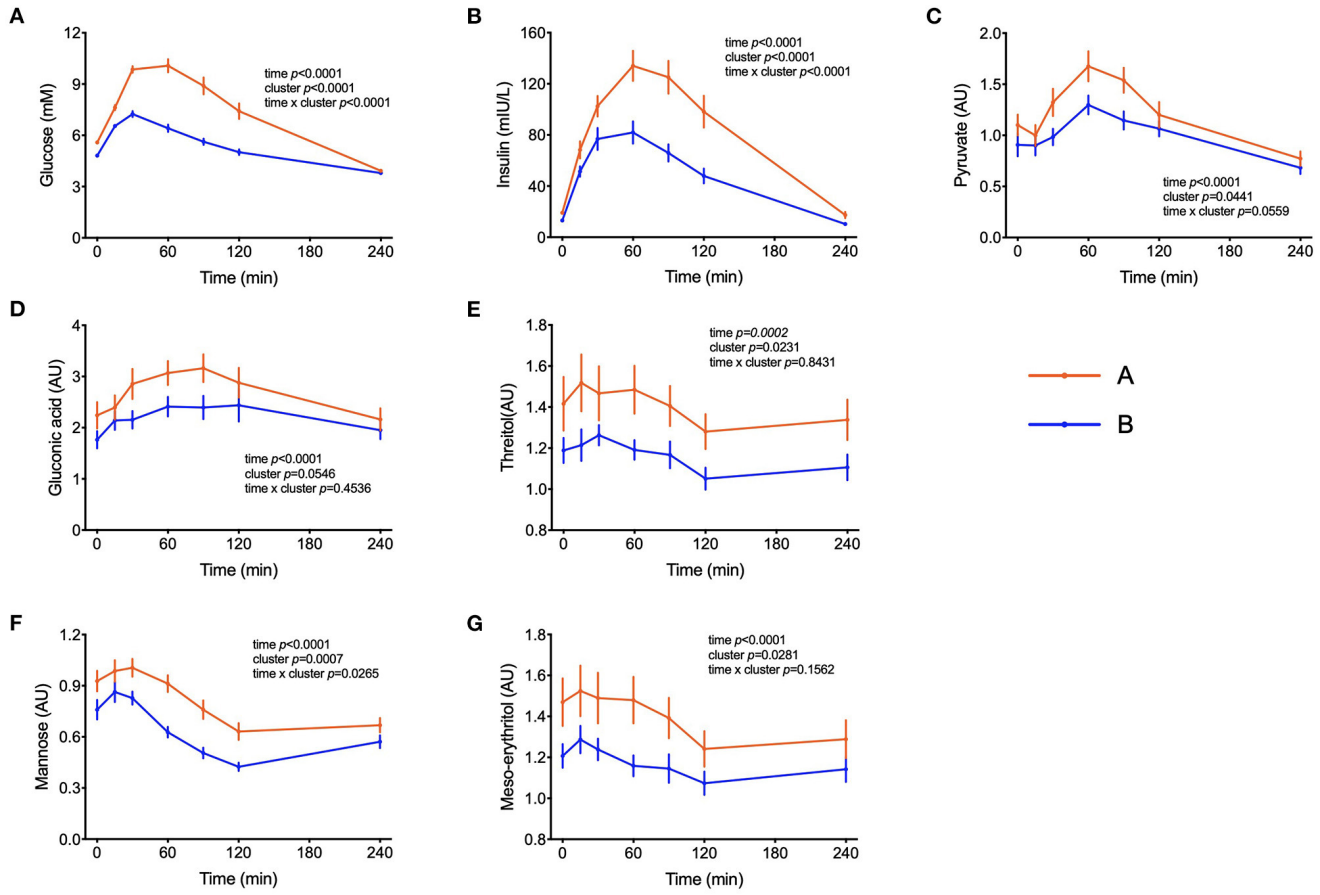
Handling of glucose and other sugars and sugar-derivatives. **(A–G)** Plasma glucose and insulin concentrations during the OGTT and levels of pyruvate, gluconate, threitol, mannose and meso-erythritol (AU = arbitrary units). Data are presented as the means and standard errors of the mean. Results from mixed-effects analysis indicated in each graph.

The focus of this study was to identify markers of metabolic responses to an OGTT, and the chosen approach included a hierarchical cluster analysis considering glucose concentrations measured throughout the test, resulting in the identification of two groups of volunteers, named clusters A and B. As seen in Figure 1, cluster A displayed higher fasting and postprandial plasma glucose levels and an exaggerated insulin response, indicating a certain state of insulin resistance, also confirmed by the HOMA-IR values that were 60% higher than those of individuals in cluster B (Table 1). A PLS-DA model comparing clusters A and B in search of discriminant variables revealed differences between these 2 groups (the model has 1 component, R^2^ = 0,5 and Q^2^ = 0,27). A cross-validation performed using the K-fold method (Supplementary Figure 1) indicates robust differences between the two groups and the analyses of variance of the cross-validated residuals returned a *p* value = 0.000028 as statistical significance of the model.

The next step of our analysis was an univariate statistical test among the variables that received a VIP value > 1 in the PLS-DA model. Table 2 reports these variables obtained in the overnight fasting state only. Despite the absence of differences in the BMI between groups (Table 1), the content of intra-abdominal adipose tissue (IAAT) was 35% higher in cluster A as well as internal body fat and liver fat associated with cluster A. Liver fat content appeared as particularly relevant, as it revealed a strong correlation with the average glucose concentrations during the test (r = 0.69; *p* = 1.77E-10) and was 3.1-fold higher in subjects from cluster A, as compared to cluster B individuals. Interestingly, the proportion of non-abdominal subcutaneous adipose tissue (NASAT) to total body fat was higher in cluster B, suggesting that given the same amount of body fat, its higher presence in the subcutaneous depot may provide some protection from insulin resistance (Table 2). As expected, higher HbA1c concentrations were associated with cluster A, as it was also the case for malondialdehyde and s-E-selectin concentrations.

**Table 2:** Discriminant variables measured in fasting state.

	**A**			**B**			***t*** **test**		**Pearson corr x Glucose**		**A/B**
	**Mean**	**SEM**	**N**	**Mean**	**SEM**	**N**	***p*-Value**	**q value**	**rho**	***p*-Value**	
**Plasma fatty acids (μg/ml)**
C14:0	37.48	2.99	33	23.49	1.79	39	0.0001	0.0007	0.44	0.0001	1.60
C15:0	7.13	0.36	33	5.79	0.29	39	0.0040	0.0072	0.31	0.0077	1.23
C16:0	766.45	39.79	33	606.54	22.03	39	0.0005	0.0024	0.40	0.0005	1.26
C16:1 n-7	86.85	6.20	33	60.05	4.97	39	0.0011	0.0038	0.33	0.0044	1.45
C18:0	218.52	8.92	33	187.38	4.94	39	0.0022	0.0056	0.31	0.0091	1.17
C18:1 c11	61.94	3.49	33	51.18	2.20	39	0.0089	0.0131	0.30	0.0109	1.21
C18:1 c9	707.03	38.67	33	566.62	19.66	39	0.0012	0.0038	0.40	0.0005	1.25
C18:1 t6-11	41.58	3.19	33	32.67	2.29	39	0.0236	0.0258	0.23	0.0486	1.27
C18:3 n-3	24.12	2.08	33	18.87	0.81	39	0.0149	0.0203	0.31	0.0081	1.28
C20:1 n-9	5.10	0.28	33	4.33	0.17	39	0.0190	0.0220	0.33	0.0049	1.18
C20:2 n-6	7.89	0.50	33	6.51	0.32	39	0.0190	0.0220	0.25	0.0349	1.21
C20:3 n-6	44.21	2.17	33	38.49	1.72	39	0.0398	0.0402	0.24	0.0406	1.15
C22:5 n-3	21.36	1.02	33	17.26	0.53	39	0.0004	0.0023	0.31	0.0081	1.24
C22:6 n-3	80.73	4.95	33	69.13	2.82	39	0.0380	0.0394	0.14	0.2343	1.17
**Plasma fatty acids (%)**
C14:0	1.07	0.05	33	0.79	0.04	39	2.30E-05	0.0004	0.49	1.15E-05	1.37
C16:0	22.45	0.29	33	20.77	0.25	39	2.80E-05	0.0004	0.50	9.91E-06	1.08
C16:1,n-7	2.51	0.12	33	2.00	0.13	39	0.0057	0.0089	0.28	0.0198	1.25
C18:1,c9	20.58	0.46	33	19.46	0.32	39	0.0458	0.0440	0.38	0.0012	1.06
C18:2,n-6	22.24	0.52	33	25.13	0.50	39	0.0002	0.0011	−0.45	0.0001	0.89
C20:0	0.26	0.01	33	0.28	0.01	39	0.0187	0.0220	−0.34	0.0042	0.91
C22:0	0.58	0.02	33	0.67	0.02	39	0.0036	0.0068	−0.39	0.0008	0.87
C23:0	0.29	0.01	33	0.34	0.01	39	0.0054	0.0088	−0.39	0.0008	0.85
C24:0	0.63	0.02	33	0.71	0.02	39	0.0081	0.0123	−0.38	0.0011	0.88
C24:1,n-9	1.12	0.04	33	1.38	0.04	39	4.10E-05	0.0004	−0.49	1.37E-05	0.81
**Body composition/anthropometric variables**
Waist (cm)	102.24	1.77	33	97.30	1.70	39	0.0489	0.0459	0.39	0.0008	1.05
IAAT (%BW)	5.37	0.28	29	4.14	0.22	39	0.0009	0.0038	0.46	0.0001	1.30
IAAT (%TBF)	15.78	1.02	29	11.70	0.84	39	0.0028	0.0062	0.43	0.0003	1.35
IAAT (kg)	4.70	0.29	29	3.48	0.23	39	0.0012	0.0038	0.52	6.50E-06	1.35
IAAT:ASAT	0.80	0.07	29	0.53	0.05	39	0.0016	0.0046	0.39	0.0010	1.51
Internal (%TBF)	26.96	1.45	29	21.34	1.14	39	0.0030	0.0062	0.39	0.0011	1.26
Internal (%BW)	9.19	0.36	29	7.65	0.29	39	0.0014	0.0042	0.39	0.0010	1.20
Internal (kg)	8.04	0.42	29	6.38	0.33	39	0.0023	0.0056	0.47	0.0001	1.26
Internal: subcutaneous	0.38	0.03	29	0.28	0.02	39	0.0032	0.0063	0.37	0.0018	1.37
Liver lipids	7.40	1.15	29	2.38	0.28	38	0.00001	0.0004	0.69	1.77E-10	3.11
NAIAT (%TBF)	11.18	0.56	29	9.65	0.38	39	0.0225	0.0254	0.22	0.0702	1.16
NAIAT (kg)	3.34	0.17	29	2.90	0.13	39	0.0454	0.0440	0.27	0.0246	1.15
Soleus m. IMCL	18.06	1.58	29	14.73	0.88	39	0.0534	0.0480	0.21	0.0950	1.23
Tibialis m. IMCL	8.11	0.61	29	6.72	0.42	39	0.0582	0.0504	0.18	0.1507	1.21
ASAT (%TBF)	21.08	0.70	29	22.99	0.50	39	0.0260	0.0276	−0.21	0.0931	0.92
NASAT (%TBF)	51.96	1.01	29	55.67	1.00	39	0.0127	0.0179	−0.38	0.0014	0.93
SAT (%TBF)	73.04	1.45	29	78.66	1.14	39	0.0030	0.0062	−0.39	0.0011	0.93
**Metabolism markers**
HbA1c (mmol/mol Hb)	37.64	0.61	28	35.53	0.43	38	0.0049	0.0084	0.38	0.0017	1.06
MDA (μmol/L)	2.44	0.15	32	1.96	0.13	38	0.0186	0.0220	0.36	0.0025	1.24
s-E-Selectin (ng/mL)	41.85	3.81	33	29.33	3.52	39	0.0185	0.0220	0.34	0.0032	1.43
Adiponectin (μg/mL)	13.32	0.96	33	16.67	1.39	39	0.0589	0.0504	−0.40	0.0007	0.80

*Only variables with a VIP value > 1 in the PLS-DA model are depicted in this table. The differences between the two clusters were tested with a T-test and their correlation (Pearson) with the average glucose concentration during the OGTT presented. The right (last) column presents the ratio of each variable between clusters A and B individuals. Highlighted rows indicate variables associated with cluster B. Variables that were not considered different between the two clusters after the T-test are not included in the table. ASAT, abdominal subcutaneous adipose tissue; AT, adipose tissue; IAAT, intra-abdominal adipose tissue; IMCL, intramyocellular lipids; MDA, malondialdehyde; NAIAT, non-abdominal internal adipose tissue; NASA, non-abdominal subcutaneous adipose tissue; SAT, subcutaneous adipose tissue. Total internal AT (Internal) was subdivided into Intra-abdominal AT (IAAT) and non-abdominal internal AT (NAIAT). Total Subcutaneous AT (SAT) was subdivided into abdominal subcutaneous AT (ASAT) and non-abdominal subcutaneous AT, with the abdominal region defined as the region between the top of the liver and the femoral heads. Adipose tissue was expressed relative to total body weight (%BW) or relative to total fat mass (%TBF)*.

Several fatty acids (FA) displayed higher overnight fasting concentrations in cluster A as compared to cluster B subjects. Myristic acid (C14:0) deserves particular attention, as its concentration, despite corresponding to only ~1% of total FA, was 60% higher in cluster A and had the highest correlation among FA with the average glucose concentrations during the test (r = 0.44; *p* = 0.0001). Palmitic acid (C16:0) revealed a similar behavior, but even oleic (C18:1c9) and linolenic (C18:3n3) acids - often classified as beneficial FA - were most abundant in cluster A (Table 2). In the composition of the FA profile (as % of total), very long chain fatty acids such as lignoceric (C24:0) and nervonic (C24:1n9) had around 15% higher concentration than cluster A and their level was negatively correlated (r = −0.38 and −0.49; *p* < 0.001) with the average glucose concentrations during the OGTT (Table 2).

Peak plasma glucose reached Cmax at t = 30 min in both groups but individuals in cluster A displayed a 77% increase in comparison to fasting values, whereas in cluster B it increased by only 50%. After 120 min, plasma glucose levels in individuals from cluster B already returned to fasting values, whereas in cluster A these values were still 32% higher (*p* = 0.0001) than fasting levels (Figure 1A). Plasma insulin concentrations reached also Cmax at 30 min in cluster B but kept increasing till t = 60 min in cluster A, reaching values 65% higher (*p* = 0.0005) than in cluster B (Figure 1B). The concentrations of pyruvate, mannose, gluconic acid, threitol and meso-erythritol were also higher in individuals from cluster A (Figure 1 and Table 3).

**Table 3:** Discriminant variables measured during the OGTT.

	**A**			**B**			***t*** **test**		**Pearson corr x Glucose**		**A/B**
	**Mean**	**SEM**	**N**	**Mean**	**SEM**	**N**	***p*-Value**	**q value**	**rho**	***p*-value**	
**Bile acids**
TUDCA (%)	0.33	0.06	30	0.20	0.02	35	0.021	0.087	0.06	0.6505	1.67
Unconj. BA (%)	38.59	2.83	33	31.36	2.67	39	0.068	0.132	0.24	0.0414	1.23
Conj./ Unconj. BA	2.95	0.41	33	4.44	0.53	39	0.034	0.088	−0.31	0.0079	0.66
Gly-conj. BA (%)	51.85	2.26	33	60.36	2.28	39	0.011	0.068	−0.28	0.0179	0.86
Gly-conj. BA (nmol/L)	1,597.61	127.71	33	2,345.40	273.06	39	0.022	0.087	−0.26	0.0262	0.68
GCDCA (nmol/L)	853.72	79.15	33	1,164.85	106.33	39	0.026	0.087	−0.25	0.0380	0.73
**Fatty acid-derived acylcarnitines**
C14 (μmol/L)	0.02	0.00	33	0.02	0.00	39	0.028	0.087	0.35	0.0028	1.18
C16 (μmol/L)	0.11	0.01	33	0.10	0.00	39	0.033	0.088	0.39	0.0007	1.14
C18 (μmol/L)	0.03	0.00	33	0.02	0.00	39	0.039	0.096	0.31	0.0083	1.16
C3 (μmol/L)	0.34	0.02	33	0.26	0.01	39	0.004	0.055	0.47	3.13E-05	1.28
C4 (μmol/L)	0.07	0.01	33	0.05	0.00	39	0.018	0.087	0.37	0.0014	1.37
C6 (μmol/L)	0.04	0.00	33	0.03	0.00	39	0.018	0.087	0.39	0.0008	1.34
**Amino acids and amino acid-related metabolites**
Isovaleryl-carnitine (μmol/L)	0.08	0.01	33	0.06	0.00	39	0.011	0.068	0.56	4.00E-07	1.25
Spermidine (μmol/L)	0.18	0.03	33	0.12	0.01	39	0.041	0.097	0.41	0.0004	1.53
Spermine (μmol/L)	0.25	0.06	33	0.13	0.01	39	0.031	0.087	0.33	0.0052	1.90
Urea (mmol/L)	5.94	0.21	33	5.34	0.20	39	0.047	0.100	0.13	0.2626	1.11
Free carnitine (μmol/L)	39.65	1.49	33	35.94	1.30	39	0.064	0.129	0.26	0.0290	1.10
Glu (μmol/L)	51.52	4.43	33	31.41	2.39	39	0.0001	0.0017	0.64	1.39E-09	1.64
Ile (μmol/L)	59.61	2.20	33	53.16	1.90	39	0.029	0.087	0.56	3.31E-07	1.12
Leu (μmol/L)	114.85	4.51	33	101.89	3.12	39	0.018	0.087	0.52	2.21E-06	1.13
Phe (μmol/L)	53.84	1.38	33	49.93	1.09	39	0.028	0.087	0.30	0.0114	1.08
Trp (μmol/L)	54.19	1.26	33	49.66	1.20	39	0.011	0.068	0.33	0.0051	1.09
Val (μmol/L)	202.08	6.18	33	186.47	4.78	39	0.046	0.100	0.53	1.30E-06	1.08
Gln (μmol/L)	575.08	11.52	33	622.93	12.04	39	0.006	0.056	−0.29	0.0140	0.92
Gly (μmol/L)	206.06	9.64	33	247.27	12.05	39	0.011	0.068	−0.31	0.0077	0.83
Ser (μmol/L)	92.49	2.74	33	101.72	3.18	39	0.035	0.088	−0.22	0.0602	0.91
**Glucose metabolism**
Glucose (mmol/L)	7.62	0.21	33	5.64	0.09	39	<0.000001	<0.000001	1		1.35
Insulin (mIU/L)	80.99	5.94	33	49.65	4.13	39	0.00003	0.00130	0.56	2.87E-07	1.63
Mannose (AU)	0.82	0.05	33	0.69	0.05	39	0.056	0.116	0.47	2.95E-05	1.18
Meso-Erythritol (AU)	1.42	0.10	33	1.18	0.05	39	0.030	0.087	0.24	0.0413	1.20
Threitol (AU)	1.41	0.10	33	1.17	0.04	39	0.023	0.087	0.27	0.0225	1.21
Pyruvate (AU)	1.22	0.10	33	0.98	0.07	39	0.042	0.097	0.37	0.0013	1.25
**Lipids**
Palmitic acid (AU)	2.35	0.12	33	2.01	0.09	39	0.029	0.087	0.31	0.0080	1.17
Stearic acid (AU)	2.13	0.09	33	1.81	0.07	39	0.005	0.055	0.32	0.0060	1.18
Cholesterol (AU)	2.57	0.19	33	2.09	0.14	37	0.045	0.100	0.16	0.1951	1.23
Triglycerides (mmol/L)	1.52	0.08	33	1.09	0.06	39	0.0001	0.0017	0.52	2.37E-06	1.39
**Leucocytes**
Monocytes (giga/L)	0.50	0.03	33	0.39	0.02	39	0.005	0.055	0.25	0.0340	1.27
Neutrophils (giga/L)	3.88	0.46	33	2.83	0.14	39	0.023	0.087	0.23	0.0518	1.37
White blood cells (giga/L)	6.31	0.49	33	4.98	0.18	39	0.008	0.068	0.27	0.0206	1.27

*The variables measured in samples collected during the OGTT that received a VIP value > 1 in the PLS-DA model at a minimum of two time points are presented with the average concentration during the test. The differences between the 2 clusters were tested with a T-test and their correlation (Pearson) with the average glucose concentration during the OGTT is presented. The right (last) column presents the ratio of each variable between cluster A and B individuals. Highlighted rows indicate variables predominantly associated with cluster B. Variables not considered different between the two clusters after the T-test were not included in the table*.

**Figure 2:**
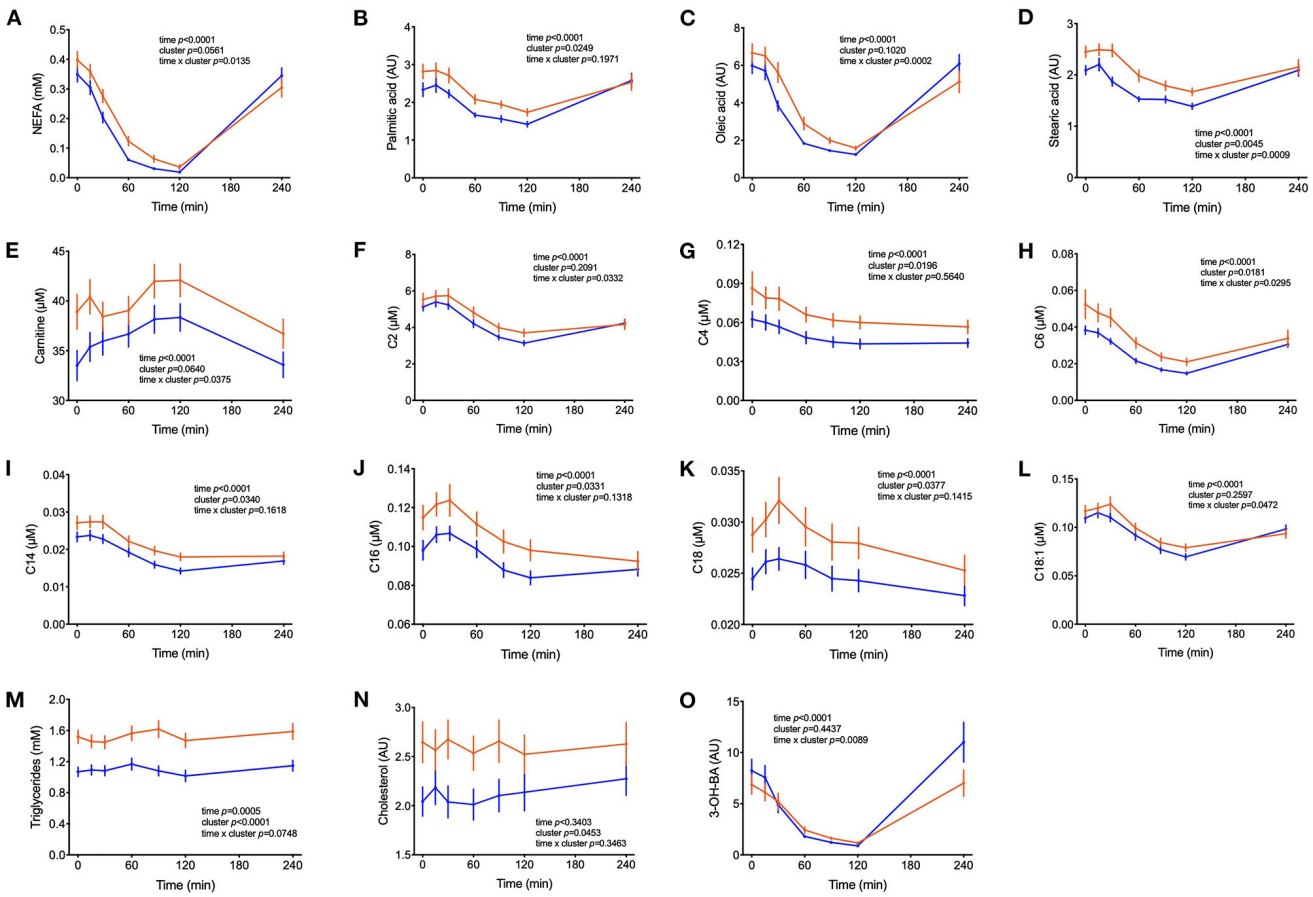
Lipid metabolism. **(A–O)** Plasma concentrations of the sum of non-esterified fatty acids, free palmitic, oleic and stearic acids, free carnitine and fatty acid-derived acylcarnitines, triglycerides, cholesterol and 3-hydroxy-butyric acid (AU = arbitrary units). Data are presented as means and standard errors of mean. Results from mixed-effects analysis indicated in each graph.

We previously reported that markers of lipid catabolism in response to mixed meal tolerance test could be used as the basis for the separation of individuals into different metabotypes (14). In the present dataset, a similar observation was made. Postprandial plasma concentration of non-esterified fatty acids (NEFA) as well as individual FA such as palmitic and oleic acids and 3-hydroxy-butyrate ranked highest amongst metabolites that discriminate clusters A and B (Figure 2). As seen in Table 3, palmitate and stearate were ~17% higher in cluster A during the OGTT and were positively correlated to postprandial glucose concentration (r = 0.31, *p* = 0.008). Plasma concentration of several acylcarnitines derived from metabolization of FA displayed similar responses. Of note, miristoylcarnitine (C14) and hexanoylcarnitine (C6) displayed throughout the OGTT consistently lower plasma concentrations in cluster B, in comparison to cluster A, but positively correlated with postprandial glycaemia (Figure 2 and Table 3).

**Figure 3:**
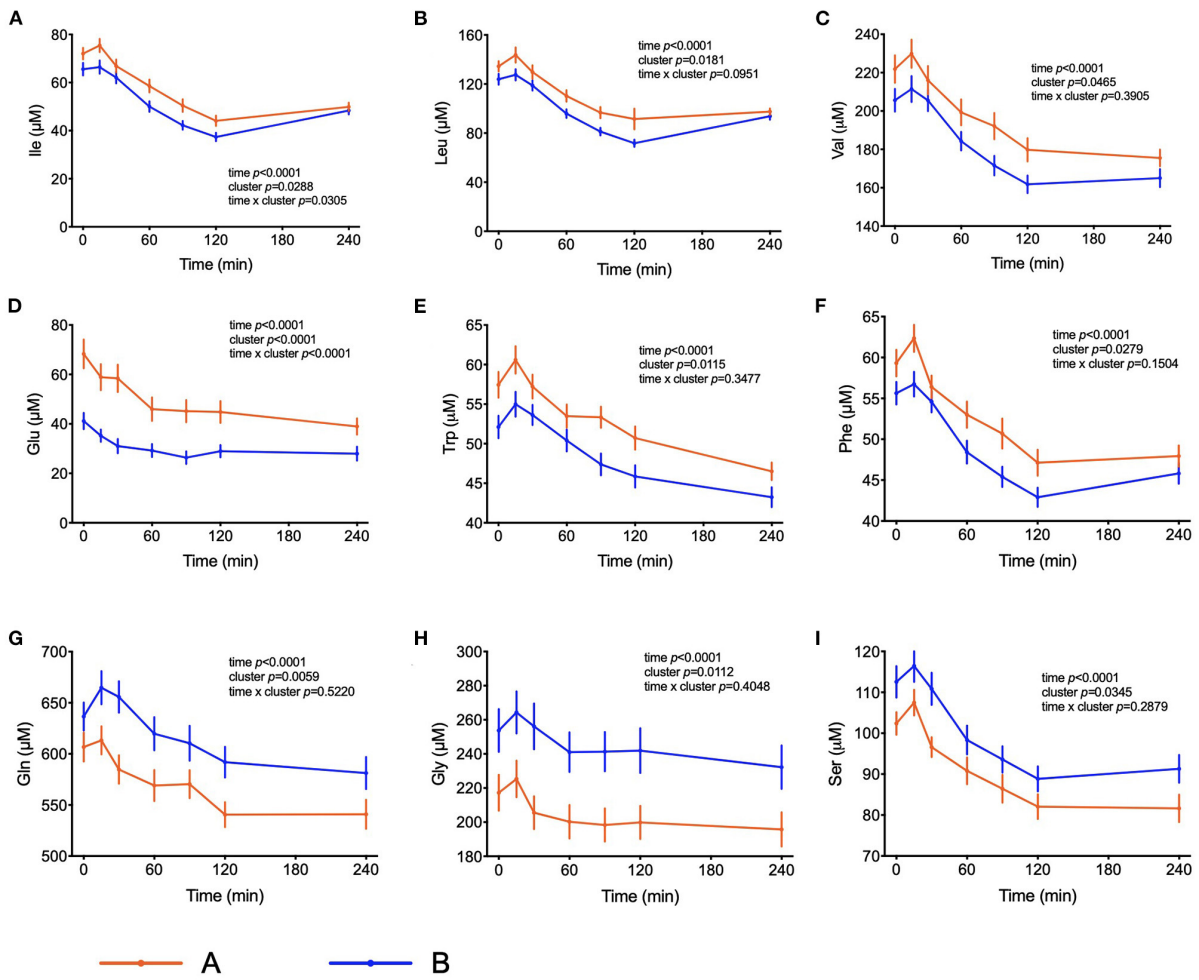
Amino acid metabolism. **(A–F)** Plasma concentrations of selected amino acids that may serve as markers of insulin resistance. **(G–I)** Plasma concentrations of amino acids associated with insulin sensitivity. Data presented as the means and the standard errors of mean. Results from mixed-effects analysis indicated in each graph.

**Figure 4:**
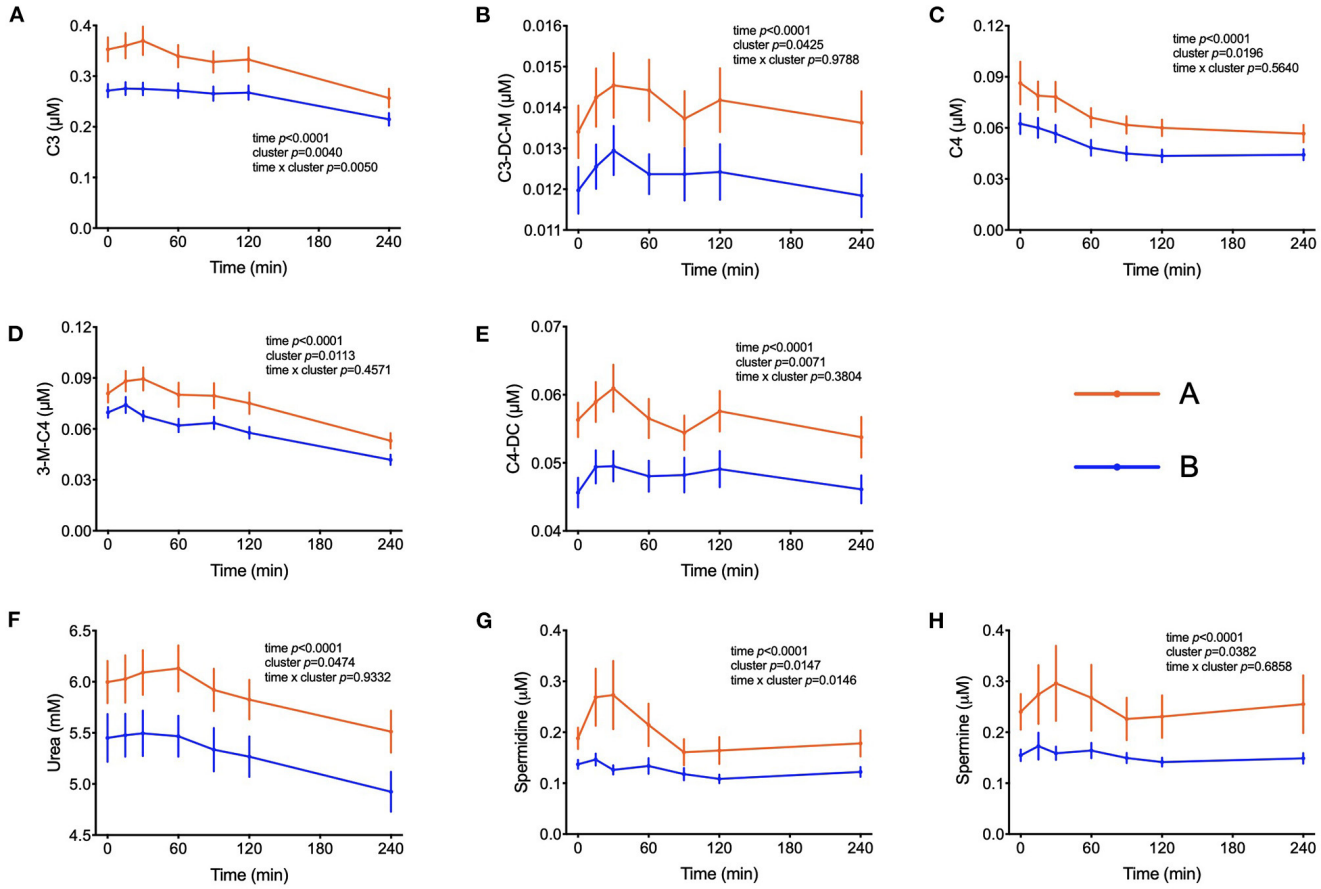
Amino acid degradation. **(A–E)** Plasma concentrations of acylcarnitines derived from the degradation of amino acids. **(F)** Plasma concentrations of urea. **(G,H)** Plasma concentration of spermidine and spermine. Data presented as means and standard errors of mean. Results from mixed-effects analysis indicated in each graph.

The amino acids BCAA, glutamate, tyrosine, tryptophan, alanine and phenylalanine were found at higher concentrations in plasma of individuals in cluster A (Figure 3) and confirm that they are linked to insulin action. These amino acids all displayed strong correlations with postprandial glucose concentration with r-values ranging from 0.3 for phenylalanine to 0.64 for glutamate (Table 3). Similarly, plasma concentration of those acylcarnitines that are derived from the degradation of amino acids such as propionylcarnitine (C3), isovaleryl-carnitine (3-M-C4) and succinylcarnitine (C4-DC) had higher levels in subjects from cluster A than from cluster B (Figure 4). Isovalerylcarnitine plasma levels were 25% higher in the plasma of subjects from cluster A and displayed a strong correlation with postprandial glucose levels (r = 0.56, *p* = 0.0000004) (Table 3). Finally, glutamine, glycine and serine, showed 8–17% lower plasma concentration in subjects from cluster A than B, confirming also previous findings in their association with insulin sensitivity (Figure 3 and Table 3). Amongst the biogenic amines spermidine and spermine were also found in higher concentrations in cluster A, as it was the case for urea, suggesting an altered amino acid handling during the OGTT in cluster A or B individuals. The average concentration of these metabolites across all 7 samples collected during the OGTT are given in Table 3.

**Figure 5:**
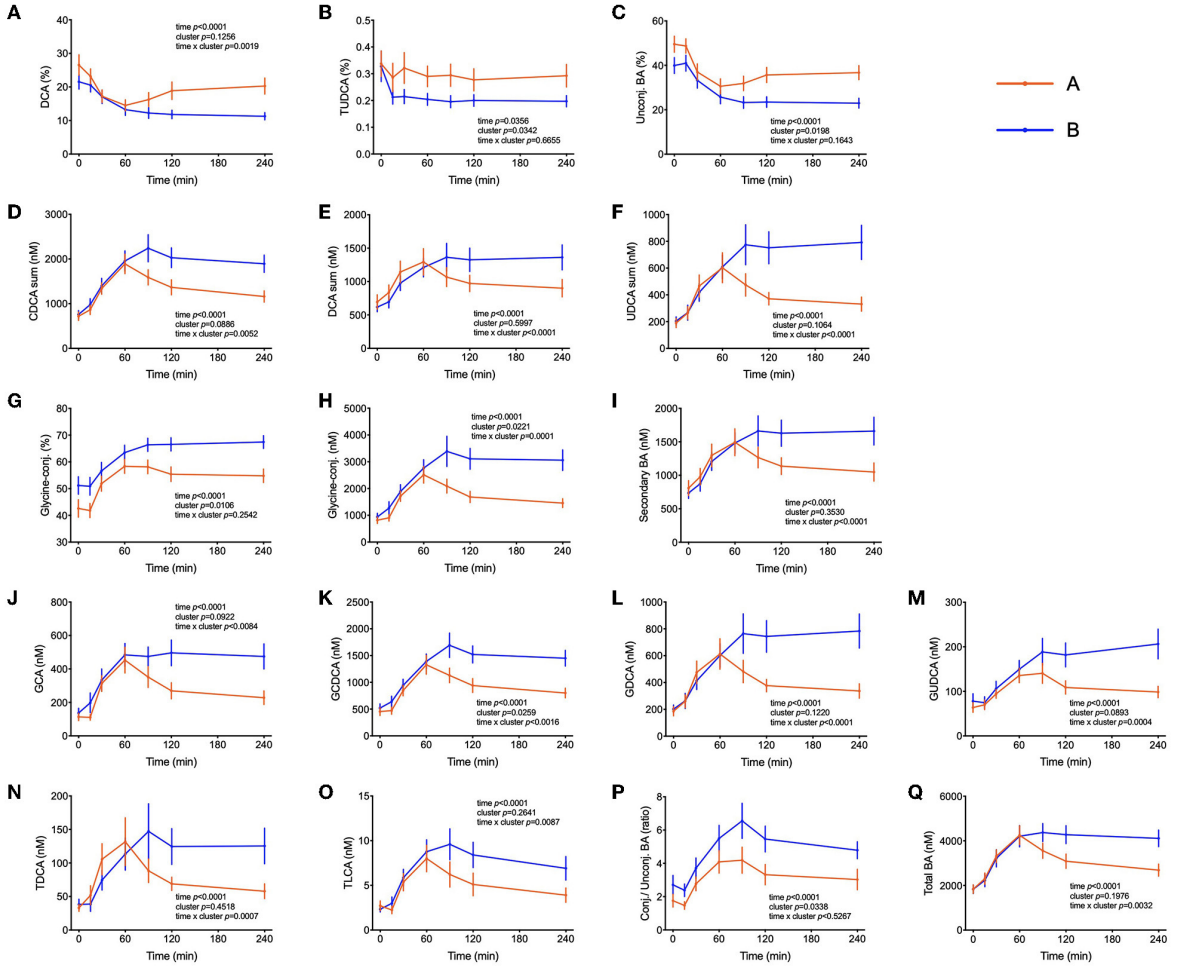
Bile acids postprandial kinetics. **(A–C)** Bile acids associated with cluster A provided as % of the total pool of plasma bile acids. **(D–Q)** Plasma concentrations of most abundant bile acids in subjects from cluster B. Data are presented as means and standard errors of the mean. Results from mixed-effects analysis indicated in each graph.

Several bile acids (BA) were amongst the metabolites identified in the PLS-DA model as most discriminant between clusters A and B. In both groups, the sum of BA reached Cmax at t = 60 min. While concentrations started to decrease in cluster A participants after this time, in cluster B the concentration of total BA were kept at maximum levels until the end of the test, remaining 38% higher in comparison to cluster A (*p* = 0.02) as shown in Figure 5. A similar pattern was observed for the sum of glycine-conjugated and secondary BA as well as for the unconjugated and glycine-conjugated forms of cholic, chenodeoxycholic, deoxycholic and ursodeoxycholic acids and the taurine-conjugated forms of deoxycholic and lithocholic acids. Interestingly, individuals from cluster A displayed a higher proportional (percentual) fraction of unconjugated BA, deoxycholic and tauroursodeoxycholic acids (Figure 5), and a lower fraction of glycine-conjugated BA and a lower ratio of conjugated: unconjugated BA (Figure 4). Glycine-conjugated BA were negatively correlated to postprandial glycaemia (r = −0.26, *p* = 0.02) (Table 3).

**Figure 6:**
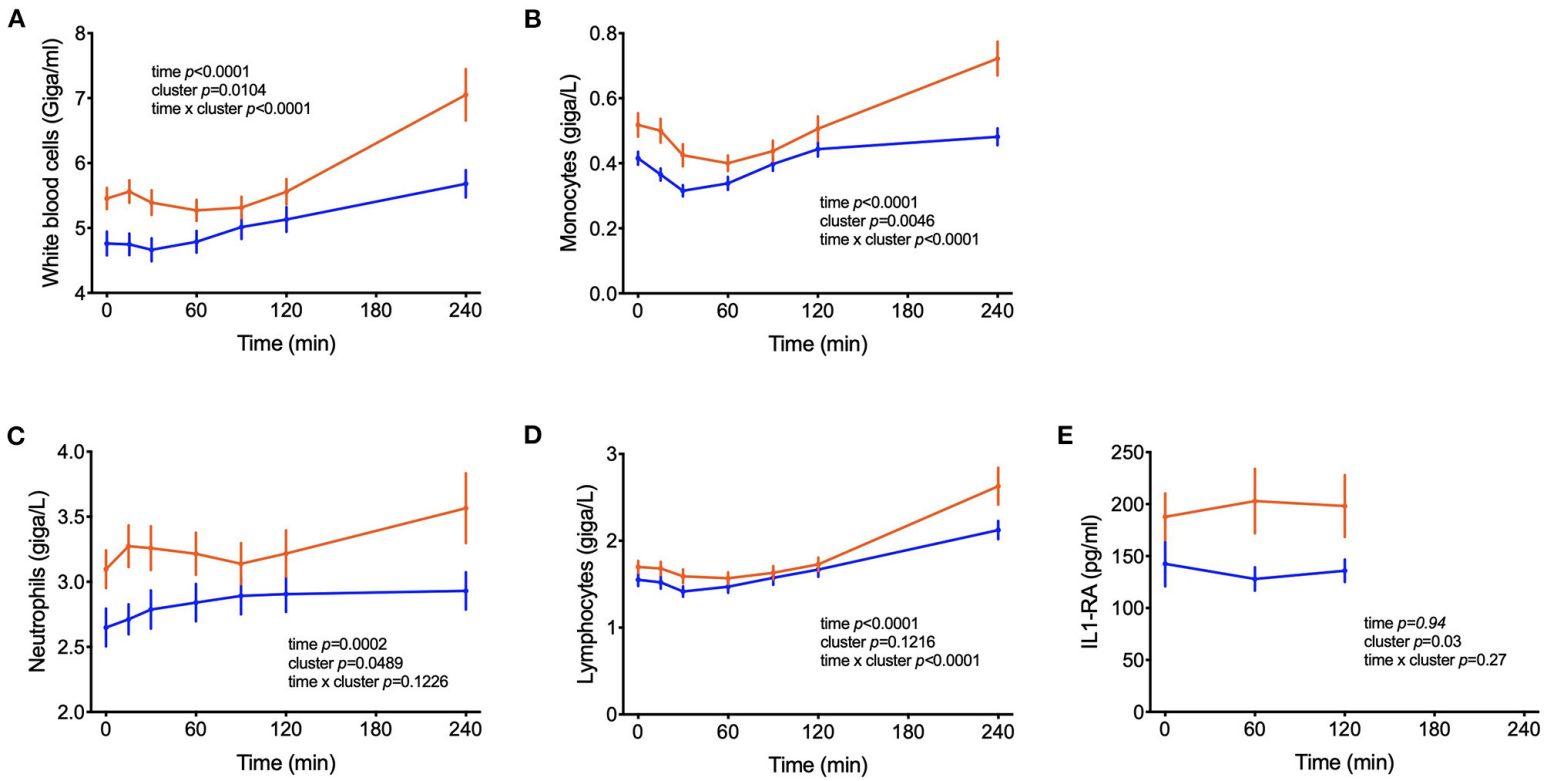
Counts of circulating leucocytes. **(A–D)** Cell counts of white blood cells, monocytes, neutrophils, lymphocytes; **(E)** IL1-RA levels in postprandial blood. Data are presented as means and standard errors of the mean. Results from mixed-effects analysis indicated in each graph.

In addition to metabolites other differences between clusters were observed in blood cell sub-populations during the OGTT. In both groups of subjects, the numbers of leucocytes in blood increased during the test, with total counts of white blood cells (WBC) reaching 23% higher counts in the 4^th^ h of the test as compared to the fasting values (*p* < 0.0001) (Figure 6). On average across the 7 blood samples collected from each subject, 26% higher counts of WBC were found in cluster A and 37 and 27% higher numbers of neutrophils and monocytes, respectively. The levels of s-E-selectin were 43% higher in subjects from cluster A in comparison to cluster B (*p* = 0.018).

**Figure 7:**
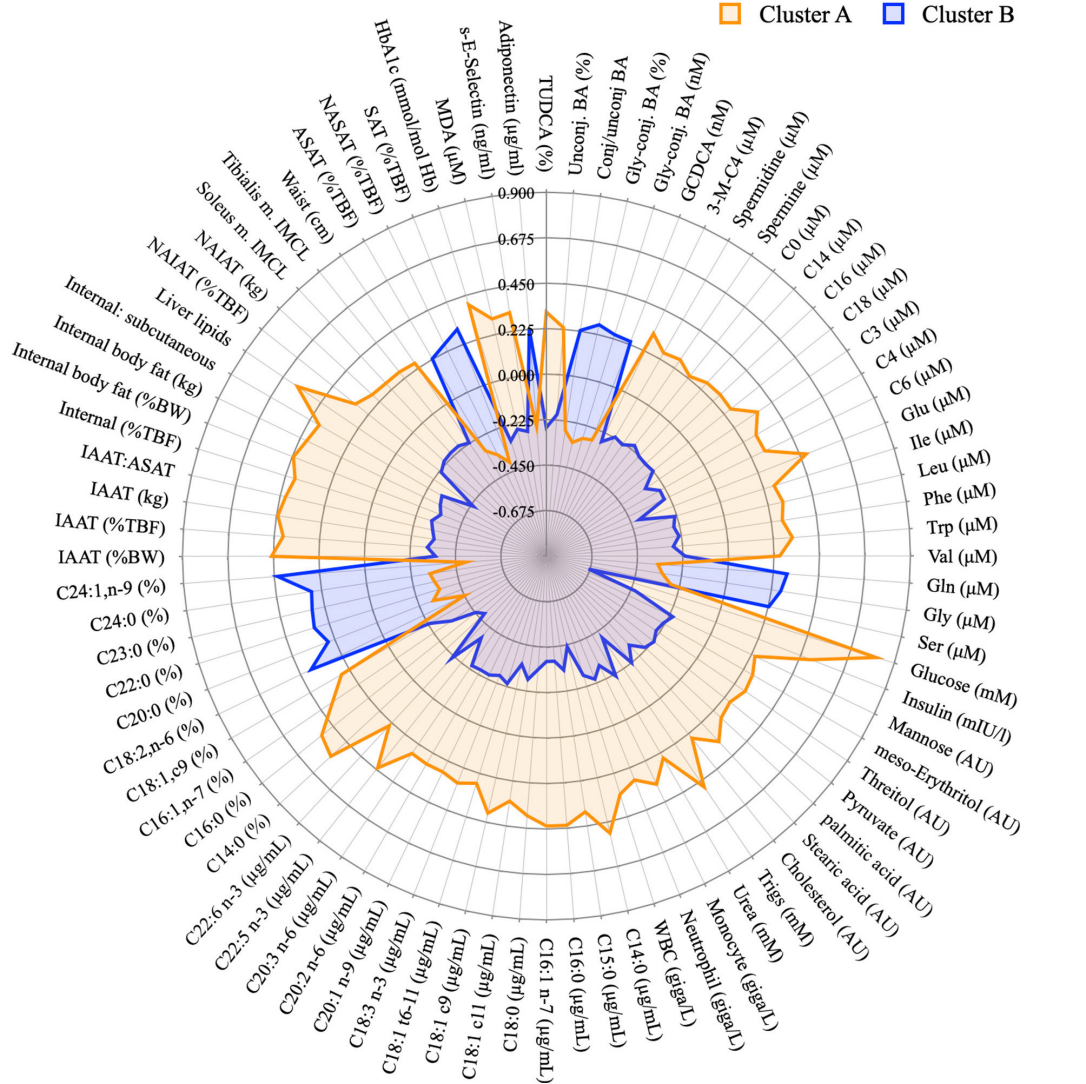
Graphical representation of the phenotypical differences between cluster A and B. Radar plot of the variables identified in the PLS-DA model as discriminant between individuals from cluster A and B and whose concentration was considered different after a T-test. Data presented as z-scores.

Figure 7 displays summary of the major differences between groups A and B considering anthropometric and body composition data, markers of clinical chemistry, and metabolites assessed in different metabolomics platforms. It clearly indicates which metabolites or other phenotypical features could be used for the “diagnosis” of glucose homeostasis. It compiles well known markers such as glucose concentration itself and liver lipids, but also less discussed markers such as different classes of BA or very long chain fatty acids.

## Discussion

According to the Word Health Organisation, impaired fasting glycemia (IFG) is characterized by fasting glucose levels between 6.1 and 7 mmol/L, while impaired glucose tolerance (IGT) is diagnosed when glucose levels are > 7 mmol/L at fasting and between 7.8 and 11 mmol/L at t = 120 minutes after the intake of 75 g glucose (24). Based on the WHO classification, only 3 subjects (4.6% of the study population) could be classified as impaired fasting glucose (IFG) and nobody fulfilled the criteria for IGT. Nevertheless, the PLS-DA model revealed 2 clear clusters that displayed distinct glucose concentrations after an overnight fasting and in the postprandial state (Table 1 and Figure 1).

All volunteers were thoroughly phenotyped including anthropometrics, clinical parameters, whole body MRI. The comprehensive metabolite profiling with hundreds of metabolites from various chemical classes revealed as one of the key findings that cluster A individuals–separated from cluster B solely by the glycemic response–had altered plasma levels of almost all known biomarkers of insulin resistance previously identified in large cohorts including increased levels of the BCAA and related acylcarnitines, reduced glycine levels and numerous altered lipid species.

Other additional marker metabolites, seldom included in other metabolite platforms are sugars and sugar-derivatives such as mannose, meso-erythritol, threitol, gluconate and pyruvate. Mannose has recently been identified in an OGTT as a monosaccharide that behaving like glucose with insulin-dependence and altered levels in prediabetic and diabetic volunteers (20). Mannose levels were strongly correlated with glycaemia (r = 0.47, *p* < 0.0001) and such a correlation between plasma mannose and glucose in the fasting state was previously described and response of plasma levels of mannose to an OGTT has also been reported (19, 25, 26). Meso-erythritol was recently associated with adiposity gain in healthy subjects and pyruvate is the ultimate precursor of liver gluconeogenesis which is important for glycemic homeostasis in the fasting state (21, 27). In both clusters, during the OGTT, pyruvate reached C_max_ at t = 60 min and thus 30 min later than the glucose peak with average postprandial pyruvate concentration exceeding those in cluster B individuals by 25% in subjects from cluster A (Table 3) as also observed by Meyer et al. (28) and Wang et al. (29).

The higher postprandial insulin increase as well as its delayed return to fasting concentrations in subjects from cluster A might have a direct effect on the postprandial concentration of markers of lipolysis and catabolism of fatty acids. Elevated insulin concentrations suppress lipolysis in the adipose tissue, leading to a decrease in NEFA levels during an OGTT that reach their nadir at t = 120 min, followed by a rebound to fasting levels at t = 240 min. This characteristic postprandial kinetic profile of fatty acid and their catabolic products is due to the coordinated regulation of hormone sensitive lipase (HSL) and lipoprotein lipase (LPL), both timely altered in activity by insulin in the postprandial period (30). Levels of NEFA, palmitic, oleic, and stearic acids were all higher in cluster A compared to B in the early phase of the OGTT (until t = 120 min) whereas after reaching the nadir, the rebound was faster in cluster B individuals. Both observations are suggestive of a more efficient and precise regulation of lipolysis by insulin, or higher metabolic flexibility in cluster B individuals. Acylcarnitines derived from fatty acid β-oxidation and 3-OH-butyrate (Figure 2) also revealed this higher responsiveness in cluster B. Fatty acids and fatty acid-derived acylcarnitines in the fasting state were increased in patients with impaired glucose tolerance as compared to individuals with normal glucose tolerance (31, 32). Nowak et al. (33) observed a decline in plasma levels of fatty acids and acylcarnitines during a glucose challenge that was blunted in participants with insulin resistance as compared to insulin sensitive individuals (33).

The increased glucose uptake into muscle, adipose tissue and liver followed by storage in glycogen (liver and muscle) or conversion into long-chain FA (liver and adipose) but also increased flux through glycolysis and TCA, cause a transient increase in plasma levels of long-chain acyl-carnitines (>16 carbon) although levels of the corresponding free FA decline simultaneously by inhibition of lipolysis. The efflux of mitochondrial-derived acylcarnitines into plasma seems to represent an overflow of substrates for oxidation (9). Although the carnitine palmitoyl transferase (CPT)-system in the inner mitochondrial membrane operates as an exchanger, the transport pathway for the cellular exit is less effective but it may also be of an exchange character as it was previously shown that free carnitine and acyl-carnitines in plasma under anabolic and catabolic conditions always behave mirror-like (4).

Elevated plasma BCAA levels and their degradation-derived acylcarnitines (C3 and C5) are established markers of insulin resistance, prediabetes and future onset of type 2 diabetes associated with an altered flux through BCAA catabolic pathways in obesity (34, 35). BCAA play a central role in insulin resistance and appear detrimental to insulin sensitivity in animals and humans (36). Amino acids may also affect a variety of other processes involved in glucose homeostasis (37). It has also been shown that amino acids influence the distribution of GLUT4-containing vesicles indicating that the insulin-dependent glucose influx mediated by an increase of GLUT4 density in the plasma membrane may already be compromised (38). It is interesting to note that troglitazone was able to increase amino acid uptake into preadipocytes, suggesting that amino acid uptake into adipose tissue is under insulin control (39). The response to the OGTT is characterized by a rapid decrease in circulating BCAA levels. We observed higher plasma BCAA concentrations among the individuals from cluster A compared to cluster B in the fasting state, which was subsequently maintained throughout the entire challenge, with the exception of the time point t = 240 min, when the differences among the two groups were no longer visible. Yet, plasma levels 4 h after the intake of glucose were still considerably below initial fasting levels. These effects may be due to a dysregulated BCAA uptake via system A transporters in the plasma membranes of insulin target organs and by changes in activity of BCAA catabolizing enzymes (40). A previous study identified expression of genes that regulate the initial, rate-limiting steps of BCAA oxidation (including BCAA transaminase 2 and inner-mitochondrial enzymes from the branched-chain alpha-ketoacid dehydrogenase complex) as dependent on the degree of insulin resistance in subjects (41). Since plasma levels of several amino acids decrease in circulation up to t = 120 min and start increasing after that time point only in individuals from cluster B, we can speculate that the absence of a rebound in subjects from cluster A is a response to the higher levels of insulin. It is possible that the higher levels of insulin that are kept higher for a longer time in these subjects is keeping the values of some amino acids (Ile, Leu and Phe) at a lower concentration even at t = 240 min. Impaired sensitivity to insulin could also be the reason behind such responses, given that membrane density and/or translocation of amino acid transporters is insulin dependent.

The PLS-DA model also identified significantly elevated plasma levels of glutamate, tryptophan and phenylalanine in cluster A subjects (Figure 3). Mean glutamate concentrations during the OGTT were 64% higher in subjects from cluster A compared to cluster B and were positively correlated with postprandial glycemic profile (r = 0.64, *p* = 1.39E-09) (Table 3). Aromatic amino acids in plasma are also related to insulin resistance, and emerged as predictors of future development of type 2 diabetes (42, 43). We found reduced levels of glycine and serine in cluster A as compared to cluster B individuals. Elevated levels of plasma glycine and serine have previously been linked to increased insulin sensitivity, with an association between high plasma glycine and decreased odds of abnormal HOMA-IR. In a recent review, White et al. (44) postulated a mechanism underlying the inverse association between glycine and BCAA levels which is based on increased nitrogen load in tissues by elevated BCAA levels which is dissipated by using glutamate to form alanine from pyruvate by alanine transaminase (44). The depletion of pyruvate in these pathways can be replenished from glycine by serine dehydratase and serine hydroxymethyl transferase, thereby reducing glycine and serine levels during insulin resistance or obesity.

Higher plasma triglyceride concentrations as observed in cluster A subjects confirm and extend previous findings with higher levels and predictive quality for insulin resistance (45, 46). Although cluster A subjects had higher concentrations (μg/mL) of NEFA in the fasting state, very long-chain fatty acids (VLCFA) were consistently more abundant among individuals from cluster B and displayed a negative correlation with the average glucose concentration during the OGTT (Table 2). These observations can also be extended to linoleic acid. Previous studies have described such an association between VLCFA and lower incidence of diabetes, but we here show that even in healthy individuals, the percentage of these compounds within the entire plasma fatty acid profile is associated with an improved glycemic response during the OGTT (47, 48). Whereas, mechanistic explanations are still missing, the higher proportion of VLCFA has been linked to lower *de novo* lipogenesis or changes in sphingolipid metabolism (49).

High-fat and high-carbohydrate meals can trigger an acute increase of plasma inflammatory biomarkers such as IL-6 and TNF-α in the first hours of the postprandial period (50, 51). In parallel, the number of leucocytes such as macrophages and neutrophils increase, probably participating in this transient inflammatory response to the meal (52–54). This postprandial inflammation might be an important risk factor for the development of chronic diseases such as type 2 diabetes and cardiovascular disease and depends on meal composition and lifestyle factors (52, 55, 56). We report increased numbers of WBC, monocytes, lymphocytes and neutrophils in the subjects from cluster A compared to cluster B, suggesting increased inflammation in the first group. Although changes in the plasma levels of inflammatory cytokines were not observed in our study (Table 1), s-E-selectin concentrations were higher in subjects from cluster A in comparison to B, supporting the notion of an inflammatory state in cluster A individuals (Table 2). Previous reports identified an association between s-E-selectin with obesity, insulin resistance and metabolic inflexibility (57–59).

BA are released from the gallbladder following a meal by contraction mediated primarily by cholecystokinin. That glucose can elicit as well a BA secretion with an increase in plasma that follows glucose appearance has been shown before and BA are now considered to play an important role in coordinating metabolic responses during the postprandial period (60). In addition to their well-established function in facilitating dietary lipid emulsification and absorption, BA may be important signaling molecules able to exert pleiotropic physiological effects on different organs (61). Insulin resistance has been previously associated with increased plasma levels of deoxycholic acid and its conjugated forms and increments in the ratio of unconjugated/conjugated BA were attributed to subjects with high plasma concentrations of insulin, NEFA, and triglyceride levels (62). Other studies have reported higher levels of BA in diabetic patients in fasting and in the postprandial state and it has been reported that BA hydrophobicity can influence insulin resistance. In fact, DCA (a hydrophobic secondary BA) administration decreased insulin signaling and endoplasmic reticulum homeostasis, exacerbating impaired glucose homeostasis in mice (63–65). The relationship between BA and glucose metabolism is long known, but the mechanisms by which BA affect glucose homeostasis and vice versa are not yet understood. In our present study with healthy individuals, there were no differences in fasting plasma BA concentrations between the two groups of individuals, but higher concentrations of BA were observed in subjects with a better glycemic response in the OGTT (cluster B) beginning 1 hour after the start of the OGTT (Figure 5). Similar results were observed by Higgins et al. (66), reporting that obese adolescents had reduced postprandial plasma BA concentrations in comparison to lean controls, despite similar fasting BA levels. Mantovani et al. (67) demonstrated also that postprandial BA concentrations were distinctly different between healthy and diabetic individuals (66, 67). Since BA concentrations in plasma increased similar in both clusters in the initial phase of the OGTT (Figure 1) their absorption from the intestine seems not to be different between cluster. However, in cluster B individuals BA levels did not decline over time as fast as in cluster A subjects suggesting that either their removal is slower than in less healthy individuals or that there is a higher flux through the enteric-hepatic cycle providing higher plasma BA levels higher throughout the test in more healthy volunteers. To find an explanation for this discrepancy more studies are needed in healthy and compromised individuals with proper reporting of time-dependent changes during an OGTT or a mixed meal test.

## Conclusion

According to their glycaemia during an OGTT, healthy adult individuals could be classified into two subgroups characterized by a multitude of metabolites with altered plasma levels. These altered metabolite levels included many known markers of obesity, insulin resistance and type 2 diabetes, that were increased in cluster A individuals already in the fasting state. Most of the plasma concentrations of these marker metabolites remained as different during the 240 min of the test, demonstrating that the time-dependent measurements during the OGTT provide no particular benefit for a sub-clustering according to metabotype. Most of the discriminant metabolites can be linked to insulin effects on uptake and utilization of glucose, amino acids and fatty acids with the alterations in plasma levels given as a signature of impaired insulin response. Differences between the clusters also included BA concentrations for which a mechanistic basis for the association cannot be provided. Moreover, higher number of leucocytes were observed in cluster A. These individuals also displayed major differences in body fat stores and fat distribution for which associations with impaired insulin signaling have been reported before. The present study reveals that a panel of metabolites in fasting plasma allows the identification of an individual as “pre-pre-diabetic” and at increased risk for diabetes that is still otherwise classified as healthy according to the definitions of the WHO.

## Data Availability Statement

The original contributions presented in the study are included in the article/Supplementary Material, further inquiries can be directed to the corresponding author.

## Ethics Statement

The studies involving human participants were reviewed and approved by Brent Ethics Committee (REC ref: 12/LO/0139) and registered at clinicaltrials.gov record: NCT01684917. The patients/participants provided their written informed consent to participate in this study.

## Author Contributions

BO, HD, JB, LB, SW, DI, CD, and GF designed the research program. JF, MR, ET, TG, DB, and YK-K performed research leading to the findings reported here. ET and JF analyzed data. DS, J-PT, SK, and KH contributed new reagents or specific analytical tools and expertise. JF, CD-P, GD, and HD wrote the manuscript. All authors reviewed the paper.

## Conflict of Interest

TG is the CEO and stockowner in Vitas Ltd. CD is a founder, stockowner, board member and consultant in Vitas Ltd. DS was an employee of biocrates life sciences AG. The remaining authors declare that the research was conducted in the absence of any commercial or financial relationships that could be construed as a potential conflict of interest.

## Publisher's Note

All claims expressed in this article are solely those of the authors and do not necessarily represent those of their affiliated organizations, or those of the publisher, the editors and the reviewers. Any product that may be evaluated in this article, or claim that may be made by its manufacturer, is not guaranteed or endorsed by the publisher.

## Supplementary Material

Supplementary Figure 1 Multivariate analysis. **(A)** Graphical representation of hierarchical cluster analysis that generated the classification of the individuals into clusters A and B. **(B)** Score plot showing the separation of cluster A and B. **(C)** Cross-validation, indicating the robustness of the model.

## References

[1] Hatting M Tavares CDJ Sharabi K Rines AK Puigserver P. Insulin regulation of gluconeogenesis. Ann N Y Acad Sci. (2018) 1411:21–35. 10.1111/nyas.13435 28868790 PMC5927596

[2] Galgani JE Moro C Ravussin E. Metabolic flexibility and insulin resistance. Am J Physiol Endocrinol Metab. (2008) 295:1009–17. 10.1152/ajpendo.90558.2008 18765680 PMC2584808

[3] Legro RS Castracane D Kauffman RP. Detecting insulin resistance in polycystic ovary syndrome: purposes and pitfalls. Obstet Gynecol Surv. (2004) 59:141–54. 10.1097/01.OGX.0000109523.25076.E2 14752302

[4] Krug S Kastenmüller G Stückler F Rist MJ Skurk T Sailer M. The dynamic range of the human metabolome revealed by challenges. FASEB J. (2012) 26:2607–19. 10.1096/fj.11-198093 22426117

[5] Zhao X Fritsche J Wang J Chen J Rittig K Schmitt-Kopplin P. Metabonomic fingerprints of fasting plasma and spot urine reveal human pre-diabetic metabolic traits. Metabolomics. (2010) 6:362–74. 10.1007/s11306-010-0203-1 20676218 PMC2899018

[6] LaBarre JL Singer K Burant CF. Advantages of studying the metabolome in response to mixed-macronutrient challenges and suggestions for future research designs. J Nutr. (2021) 151:2868–81. 10.1093/jn/nxab223 34255076 PMC8681069

[7] Rhee EP Cheng S Larson MG Walford GA Lewis GD McCabe E. Lipid profiling identifies a triacylglycerol signature of insulin resistance and improves diabetes prediction in humans. J Clin Investig. (2011) 121:1402–11. 10.1172/JCI44442 21403394 PMC3069773

[8] Würtz P Mäkinen VP Soininen P Kangas AJ Tukiainen T Kettunen J. Metabolic signatures of insulin resistance in 7,098 young adults. Diabetes. (2012) 61:1372–80. 10.2337/db11-1355 22511205 PMC3357275

[9] Newgard CB. Metabolomics and metabolic diseases: where do we stand? Cell Metab. (2017) 25:43–56. 10.1016/j.cmet.2016.09.018 28094011 PMC5245686

[10] Lépine G Tremblay-Franco M Bouder S Dimina L Fouillet H Mariotti F. Investigating the postprandial metabolome after challenge tests to assess metabolic flexibility and dysregulations associated with cardiometabolic diseases. Nutrients. (2022) 14:472. 10.3390/nu14030472 35276829 PMC8840206

[11] Gavaghan CL Holmes E Lenz E Wilson ID Nicholson JK. An NMR-based metabonomic approach to investigate the biochemical consequences of genetic strain differences: application to the C57BL10J and Alpk:ApfCD mouse. FEBS Lett. (2000) 484:169–74. 10.1016/S0014-5793(00)02147-5 11078872

[12] Stella C Beckwith-Hall B Cloarec O Holmes E Lindon JC Powell J. Susceptibility of human metabolic phenotypes to dietary modulation. J Proteome Res. (2006) 5:2780–8. 10.1021/pr060265y 17022649

[13] Morris C O'Grada C Ryan M Roche HM Gibney MJ Gibney ER. Identification of differential responses to an oral glucose tolerance test in healthy adults. PLoS ONE. (2013) 8:e72890. 10.1371/journal.pone.0072890 23991163 PMC3749984

[14] Fiamoncini J Rundle M Gibbons H Thomas EL Geillinger-Kästle K Bunzel D. Plasma metabolome analysis identifies distinct human metabotypes in the postprandial state with different susceptibility to weight loss-mediated metabolic improvements. FASEB J. (2018) 32:5447–58. 10.1096/fj.201800330R 29718708

[15] Adrian TE Ferri GL Bacarese-Hamilton AJ Fuessl HS Polak JM Bloom SR. Human distribution and release of a putative new gut hormone, peptide YY. Gastroenterology. (1985) 89:1070–7. 10.1016/0016-5085(85)90211-2 3840109

[16] Kreymann B Williams G Ghatei M Bloom S. Glucagon-like peptide-1 7-36: a physiological incretin in man. The Lancet. (1987) 5:1300–4. 10.1016/S0140-6736(87)91194-9 2890903

[17] Vinknes KJ Elshorbagy AK Nurk E Drevon CA Gjesdal CG Tell GS. Plasma stearoyl-CoA desaturase indices: association with lifestyle, diet, and body composition. Obesity. (2013) 21:E294–302. 10.1002/oby.20011 23404690

[18] Thomas EL Parkinson JR Frost GS Goldstone AP Doré CJ McCarthy JP. The missing risk: MRI and MRS phenotyping of abdominal adiposity and ectopic fat. Obesity. (2012) 20, 76–87. 10.1038/oby.2011.142 21660078

[19] Yoshimura K Hirano S Takata H Funakoshi S Ohmi S Amano E. Plasma mannose level, a putative indicator of glycogenolysis, and glucose tolerance in Japanese individuals. J Diabetes Investig. (2017) 8:489–95. 10.1111/jdi.12622 28084015 PMC5497030

[20] Mack CI Ferrario PG Weinert CH Egert B Hoefle AS Lee Y-M. Exploring the diversity of sugar compounds in healthy, prediabetic, and diabetic volunteers. Mol Nutr Food Res. (2020) 64:1–14. 10.1002/mnfr.201901190 32170825

[21] Hootman KC Trezzi JP Kraemer L Burwell LS Dong X Guertin KA. Erythritol is a pentose-phosphate pathway metabolite and associated with adiposity gain in young adults. Proc Nat Acad Sci. (2017) 114:E4233–40. 10.1073/pnas.1620079114 28484010 PMC5448202

[22] Giesbertz P Ecker J Haag A Spanier B Daniel H. An LC-MS/MS method to quantify acylcarnitine species including isomeric and odd-numbered forms in plasma and tissues. J Lipid Res. (2015) 56:2029–39. 10.1194/jlr.D061721 26239049 PMC4583086

[23] Fiamoncini J Yiorkas AM Gedrich K Rundle M Alsters SI Roeselers G. Determinants of postprandial plasma bile acid kinetics in human volunteers. Am J Physiol Gastrointest Liver Physiol. (2017) 313:G300–12. 10.1152/ajpgi.00157.2017 28663304

[24] Alberti KGMM Zimmet PZ. Definition, diagnosis and classification of diabetes mellitus and its complications part 1: diagnosis and classification of diabetes mellitus provisional report of a WHO Consultation. Diab Med. (1998) 15:539–53. 10.1002/(SICI)1096-9136(199807)15:7<539::AID-DIA668>3.0.CO;2-S 9686693

[25] Sone H Shimano H Ebinuma H Takahashi A Yano Y Iida KT. Physiological changes in circulating mannose levels in normal, glucose-intolerant, and diabetic subjects. Metabolism. (2003) 52:1019–27. 10.1016/S0026-0495(03)00153-7 12898467

[26] Amano E Funakoshi S Yoshimura K Hirano S Ohmi S Takata H. Fasting plasma mannose levels are associated with insulin sensitivity independent of BMI in Japanese individuals with diabetes. Diabetol Metab Syndr. (2018) 10:1–7. 10.1186/s13098-018-0391-9 30534205 PMC6280490

[27] Dimitriadis GD Maratou E Kountouri A Board M Lambadiari V. Regulation of postabsorptive and postprandial glucose metabolism by insulin-dependent and insulin-independent mechanisms: an integrative approach. Nutrients. (2021) 13:1–33. 10.3390/nu13010159 33419065 PMC7825450

[28] Meyer FL Mattox H Bolick M Macdonald C. Metabolic changes after test meaIs with different carbohydrates: blood levels of pyruvic acid, glucose, and lactic dehydrogenase. Am J Clin Nutr. (1971) 24:615–21. 10.1093/ajcn/24.6.615 5581001

[29] Wang Q Jokelainen J Auvinen J Puukka K Keinänen-Kiukaanniemi S Järvelin MR. Insulin resistance and systemic metabolic changes in oral glucose tolerance test in 5340 individuals: an interventional study. BMC Med. (2019) 17:1–12. 10.1186/s12916-019-1440-4 31779625 PMC6883544

[30] Frayn KN Coppack SW Fielding BA Humphreys SM. Coordinated regulation of hormone-sensitive lipase and lipoprotein lipase in human adipose tissue *in vivo*: implications for the control of fat storage and fat mobilization. Adv Enzyme Regul. (1995) 35:163–78. 10.1016/0065-2571(94)00011-Q 7572342

[31] Hanefeld M Koehler C Fuecker K Chem D Henkel E Schaper F. Insulin secretion and insulin sensitivity pattern is different in isolated impaired glucose tolerance and impaired fasting glucose. Diabetes Care. (2003) 26:868–74. 10.2337/diacare.26.3.868 12610051

[32] Mai M Tönjes A Kovacs P Stumvoll M Fiedler GM Leichtle AB. Serum levels of acylcarnitines are altered in prediabetic conditions. PLoS ONE. (2013) 8:e82459. 10.1371/journal.pone.0082459 24358186 PMC3865089

[33] Nowak C Hetty S Salihovic S Castillejo-Lopez C Ganna A Cook NL. Glucose challenge metabolomics implicates medium-chain acylcarnitines in insulin resistance. Sci Rep. (2018) 8:1–10. 10.1038/s41598-018-26701-0 29875472 PMC5989236

[34] Glynn EL Piner LW Huffman KM Slentz CA Elliot-Penry L AbouAssi H. Impact of combined resistance and aerobic exercise training on branched-chain amino acid turnover, glycine metabolism and insulin sensitivity in overweight humans. Diabetologia. (2015) 58:2324–35. 10.1007/s00125-015-3705-6 26254576 PMC4793723

[35] Gar C Rottenkolber M Prehn C Adamski J Seissler J Lechner A. Serum and plasma amino acids as markers of prediabetes, insulin resistance, and incident diabetes. Crit Rev Clin Lab Sci. (2018) 55:21–32. 10.1080/10408363.2017.1414143 29239245

[36] Zhou M Shao J Wu CY Shu L Dong W Liu Y. Targeting BCAA catabolism to treat obesity-associated insulin resistance. Diabetes. (2019) 68:1730–46. 10.2337/db18-0927 31167878 PMC6702639

[37] Bröer S. Amino acid transporters as modulators of glucose homeostasis. Trends Endocrinol Metab. (2022) 33:120–35. 10.1016/j.tem.2021.11.004 34924221

[38] Bogan JS Mckee AE Lodish HF. Insulin-responsive compartments containing GLUT4 in 3T3-L1 and CHO cells: regulation by amino acid concentrations. Mol Cell Biol. (2001) 21:4785–806. 10.1128/MCB.21.14.4785-4806.2001 11416153 PMC87167

[39] Su TZ Wang M Oxender DL Saltiel AR. Troglitazone increases system A amino acid transport in 3T3-L1 cells. Endocrinology. (1998) 139:832–7. 10.1210/endo.139.3.5795 9492010

[40] Gannon NP Schnuck JK Vaughan RA. BCAA metabolism and insulin sensitivity – dysregulated by metabolic status? Mol Nutr Food Res. (2018) 62:1700756. 10.1002/mnfr.201700756 29377510

[41] Sears DD Hsiao G Hsiao A Yu JG Courtney CH Ofrecio JM. Mechanisms of human insulin resistance and thiazolidinedione-mediated insulin sensitization. PNAS. (2009) 3:18745–50. 10.1073/pnas.0903032106 19841271 PMC2763882

[42] Wang TJ Larson MG Vasan RS Cheng S Rhee EP McCabe E. Metabolite profiles and the risk of developing diabetes. Nat Med. (2011) 17:448–53. 10.1038/nm.2307 21423183 PMC3126616

[43] Floegel A Stefan N Yu Z Mühlenbruch K Drogan D Joost HG. Identification of serum metabolites associated with risk of type 2 diabetes using a targeted metabolomic approach. Diabetes. (2013) 62:639–48. 10.2337/db12-0495 23043162 PMC3554384

[44] White PJ McGarrah RW Herman MA Bain JR Shah SH Newgard CB. Insulin action, type 2 diabetes, and branched-chain amino acids: a two-way street. Mol Metab. (2021) 52:101261. 10.1016/j.molmet.2021.101261 34044180 PMC8513145

[45] Khan SH Sobia F Niazi NK Manzoor SM Fazal N Ahmad F. Metabolic clustering of risk factors: evaluation of triglyceride-glucose index (TyG index) for evaluation of insulin resistance. Diabetol Metab Syndr. (2018) 10:1–8. 10.1186/s13098-018-0376-8 30323862 PMC6173832

[46] Mazidi M Kengne AP Katsiki N Mikhailidis DP Banach M. Lipid accumulation product and triglycerides/glucose index are useful predictors of insulin resistance. J Diabetes Complications. (2018) 32:266–70. 10.1016/j.jdiacomp.2017.10.007 29395839

[47] Lemaitre RN Fretts AM Sitlani CM Biggs ML Mukamal K King IB. Plasma phospholipid very-long-chain saturated fatty acids and incident diabetes in older adults: the cardiovascular health study. Am J Clin Nutr. (2015) 101:1047–54. 10.3945/ajcn.114.101857 25787996 PMC4409688

[48] Fretts AM Imamura F Marklund M Micha R Wu JHY Murphy RA. Associations of circulating very-long-chain saturated fatty acids and incident type 2 diabetes: A pooled analysis of prospective cohort studies. Am J Clin Nutr. (2019) 109:1216–23. 10.1093/ajcn/nqz005 30982858 PMC6500926

[49] Lemaitre RN King IB. Very long-chain saturated fatty acids and diabetes and cardiovascular disease. Curr Opin Lipidol. (2022) 33:76–82. 10.1097/MOL.0000000000000806 34907969 PMC8702474

[50] Herieka M Erridge C. High-fat meal induced postprandial inflammation. Mol Nutr Food Res. (2014) 58:136–46. 10.1002/mnfr.201300104 23847095

[51] Meessen ECE Warmbrunn MV Nieuwdorp M Soeters MR. Human postprandial nutrient metabolism and low-grade inflammation: a narrative review. Nutrients. (2019) 11:3000. 10.3390/nu11123000 31817857 PMC6950246

[52] Margioris AN. Fatty acids and postprandial inflammation. Curr Opin Clin Nutr Metab Care. (2009) 12:129–37. 10.1097/MCO.0b013e3283232a11 19202384

[53] Gregor MF Hotamisligil GS. Inflammatory mechanisms in obesity. Annu Rev Immunol. (2011) 29:415–45. 10.1146/annurev-immunol-031210-101322 21219177

[54] Kardinaal AFM van Erk MJ Dutman AE Stroeve JHM van de Steeg E Bijlsma S. Quantifying phenotypic flexibility as the response to a high-fat challenge test in different states of metabolic health. FASEB J. (2015) 29:4600–13. 10.1096/fj.14-269852 26198450

[55] Hotamisligil GS. Inflammation, metaflammation and immunometabolic disorders. Nature. (2017) 542:177–85. 10.1038/nature21363 28179656

[56] Rogero MM Calder PC. Obesity, inflammation, toll-like receptor 4 and fatty acids. Nutrients. (2018) 10:432. 10.3390/nu10040432 29601492 PMC5946217

[57] Adamska A Karczewska-Kupczewska M Nikołajuk A Otziomek E Górska M Kowalska I. Relationships of serum soluble E-selectin concentration with insulin sensitivity and metabolic flexibility in lean and obese women. Endocrine. (2014) 45:422–9. 10.1007/s12020-013-0025-9 23934358 PMC3951956

[58] Mulhem A Moulla Y Klöting N Ebert T Tönjes A Fasshauer M. Circulating cell adhesion molecules in metabolically healthy obesity. Int J Obes. (2021) 45:331–6. 10.1038/s41366-020-00667-4 32873909 PMC7840499

[59] Lee CH Kuo FC Tang WH Lu CH Su SC Liu JS. Serum E-selectin concentration is associated with risk of metabolic syndrome in females. PLoS ONE. (2019) 14:e0222815. 10.1371/journal.pone.0222815 31550292 PMC6759160

[60] Kuipers F Bloks VW Groen AK. Beyond intestinal soap - bile acids in metabolic control. Nat Rev Endocrinol. (2014) 10:488–98. 10.1038/nrendo.2014.60 24821328

[61] Perino A Demagny H Velazquez-Villegas L Schoonjans K. Molecular physiology of bile acid signaling in health, disease, and aging. Physiol Rev. (2021) 101:683–731. 10.1152/physrev.00049.2019 32790577

[62] Haeusler RA Astiarraga B Camastra S Accili D Ferrannini E. Human insulin resistance is associated with increased plasma levels of 12a-hydroxylated bile acids. Diabetes. (2013) 62:4184–91. 10.2337/db13-0639 23884887 PMC3837033

[63] Wewalka M Patti ME Barbato C Houten SM Goldfine AB. Fasting serum taurine-conjugated bile acids are elevated in type 2 diabetes and do not change with intensification of insulin. J Clin Endocrinol Metab. (2014) 99:1442–51. 10.1210/jc.2013-3367 24432996 PMC5393473

[64] Sonne DP van Nierop FS Kulik W Soeters MR Vilsbøll T Knop FK. Postprandial plasma concentrations of individual bile acids and FGF-19 in patients with type 2 diabetes. J Clin Endocrinol Metab. (2016) 101:3002–9. 10.1210/jc.2016-1607 27270475

[65] Zaborska KE Lee SA Garribay D Cha E Cummings BP. Deoxycholic acid supplementation impairs glucose homeostasis in mice. PLoS ONE. (2018) 13:e0200908. 10.1371/journal.pone.0200908 30059528 PMC6066200

[66] Higgins V Asgari S Hamilton JK Wolska A Remaley AT Hartmann B. Postprandial dyslipidemia, hyperinsulinemia, and impaired gut peptides/bile acids in adolescents with obesity. J Clin Endocrinol Metab. (2020) 105:1228–41. 10.1210/clinem/dgz261 31825485 PMC7065844

[67] Mantovani A Dalbeni A Peserico D Cattazzo F Bevilacqua M Salvagno GL. Plasma bile acid profile in patients with and without type 2 diabetes. Metabolites. (2021) 11:1–14. 10.3390/metabo11070453 34357347 PMC8304030

