# Supplementary figures and images for: Plasma Metabolic Signatures of Healthy Overweight Subjects Challenged With an Oral Glucose Tolerance Test
Source: Front Nutr. 2022 Jun 14;9:898782. doi: 10.3389/fnut.2022.898782 (PMC9237474; doi:10.3389/fnut.2022.898782)

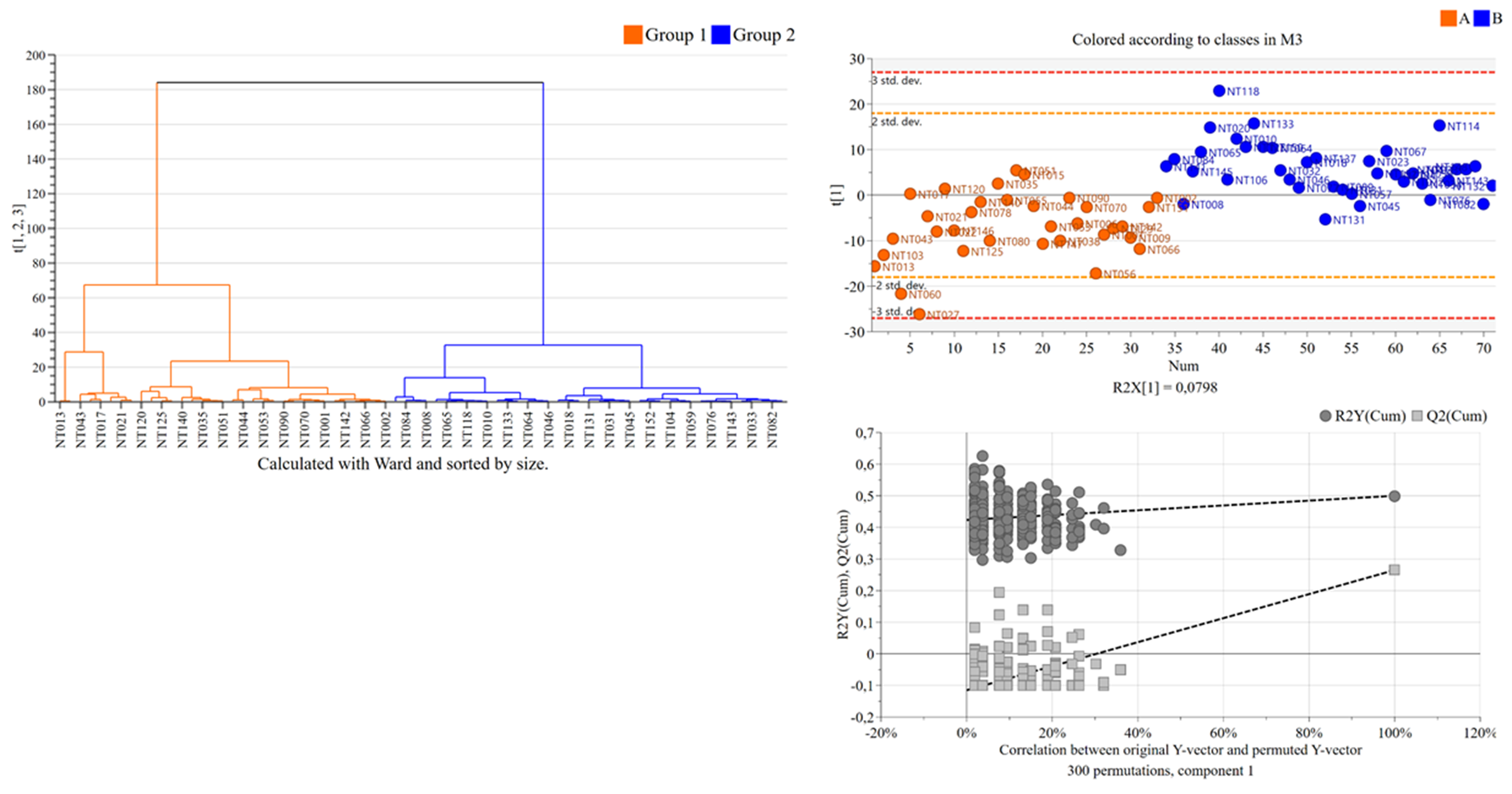

Supplement: Supplementary Figure 1 — Multivariate analysis. (A) Graphical representation of hierarchical cluster analysis that generated the classification of the individuals into clusters A and B. (B) Score plot showing the separation of cluster A and B. (C) Cross-validation, indicating the robustness of the model. [file Image_1.TIF]
