# Supplementary material for: Plasma Metabolic Signatures of Healthy Overweight Subjects Challenged With an Oral Glucose Tolerance Test
Source: Front Nutr. 2022 Jun 14;9:898782. doi: 10.3389/fnut.2022.898782 (PMC9237474; doi:10.3389/fnut.2022.898782)
Supplement: Supplementary file 2 [file Table_1.pdf]

Supplemental Table 1. Variables included in the PLS-DA model. Variables are presented with their respective VIP values and loadings, method of detection and units.

Variables included in the PLS-DA model in alphabetical order

Primary ID	Sampling time	Unit	Category	Method	VIP	Loadings
2-M-C3	0	µM	Acylcarnitine	LC-MS/MS	**0,633**	**0,0181**
2-M-C3	120	µM	Acylcarnitine	LC-MS/MS	**1,160**	**0,0332**
2-M-C3	15	µM	Acylcarnitine	LC-MS/MS	**0,648**	**0,0185**
2-M-C3	240	µM	Acylcarnitine	LC-MS/MS	**1,051**	**0,0300**
2-M-C3	30	µM	Acylcarnitine	LC-MS/MS	**0,581**	**0,0166**
2-M-C3	60	µM	Acylcarnitine	LC-MS/MS	**0,973**	**0,0278**
2-M-C3	90	µM	Acylcarnitine	LC-MS/MS	**1,221**	**0,0349**
2-M-C4	0	µM	Acylcarnitine	LC-MS/MS	**0,912**	**-0,0261**
2-M-C4	120	µM	Acylcarnitine	LC-MS/MS	**0,827**	**-0,0237**
2-M-C4	15	µM	Acylcarnitine	LC-MS/MS	**0,919**	**-0,0263**
2-M-C4	240	µM	Acylcarnitine	LC-MS/MS	**0,811**	**-0,0232**
2-M-C4	30	µM	Acylcarnitine	LC-MS/MS	**0,989**	**-0,0283**
2-M-C4	60	µM	Acylcarnitine	LC-MS/MS	**0,927**	**-0,0265**
2-M-C4	90	µM	Acylcarnitine	LC-MS/MS	**0,857**	**-0,0245**
3-Hydroxybutyric acid	0	AU	Lipid metabolism	GC-MS	**0,627**	**0,0179**
3-Hydroxybutyric acid	120	AU	Lipid metabolism	GC-MS	**1,526**	**-0,0436**
3-Hydroxybutyric acid	15	AU	Lipid metabolism	GC-MS	**0,696**	**0,0199**
3-Hydroxybutyric acid	240	AU	Lipid metabolism	GC-MS	**1,116**	**0,0319**
3-Hydroxybutyric acid	30	AU	Lipid metabolism	GC-MS	**0,187**	**-0,0053**
3-Hydroxybutyric acid	60	AU	Lipid metabolism	GC-MS	**1,284**	**-0,0367**
3-Hydroxybutyric acid	90	AU	Lipid metabolism	GC-MS	**1,818**	**-0,0520**
3-M-C4	0	µM	Acylcarnitine	LC-MS/MS	**1,260**	**-0,0360**
3-M-C4	120	µM	Acylcarnitine	LC-MS/MS	**1,568**	**-0,0449**
3-M-C4	15	µM	Acylcarnitine	LC-MS/MS	**1,285**	**-0,0368**
3-M-C4	240	µM	Acylcarnitine	LC-MS/MS	**1,294**	**-0,0370**
3-M-C4	30	µM	Acylcarnitine	LC-MS/MS	**1,894**	**-0,0541**
3-M-C4	60	µM	Acylcarnitine	LC-MS/MS	**1,438**	**-0,0411**
3-M-C4	90	µM	Acylcarnitine	LC-MS/MS	**1,305**	**-0,0373**
8-hydroxy-2'-deoxyguanosine	fasting	pg/ml	Oxidative stress	x	**0,418**	**-0,0119**
Ac-Orn	0	µM	Amino acid metabolism	Biocrates	**0,444**	**-0,0127**
Ac-Orn	120	µM	Amino acid metabolism	Biocrates	**0,445**	**-0,0127**
Ac-Orn	15	µM	Amino acid metabolism	Biocrates	**0,617**	**-0,0176**
Ac-Orn	240	µM	Amino acid metabolism	Biocrates	**0,432**	**-0,0124**
Ac-Orn	30	µM	Amino acid metabolism	Biocrates	**0,365**	**-0,0104**
Ac-Orn	60	µM	Amino acid metabolism	Biocrates	**0,377**	**-0,0108**
Ac-Orn	90	µM	Amino acid metabolism	Biocrates	**0,452**	**-0,0129**
Activity level	7 days assessm.	MET/ day	General	Accelerometer	**1,125**	**0,0322**
Adiponectin	fasting	µg/ml	Signalling/ hormone	ELISA	**1,249**	**0,0357**
ADMA	0	µM	Biogenic amine	Biocrates	**0,838**	**0,0240**
ADMA	120	µM	Biogenic amine	Biocrates	**0,763**	**0,0218**
ADMA	15	µM	Biogenic amine	Biocrates	**0,539**	**-0,0154**
ADMA	240	µM	Biogenic amine	Biocrates	**0,412**	**0,0118**
ADMA	30	µM	Biogenic amine	Biocrates	**0,869**	**-0,0249**
ADMA	60	µM	Biogenic amine	Biocrates	**0,193**	**-0,0055**
ADMA	90	µM	Biogenic amine	Biocrates	**0,314**	**-0,0090**
Ala	0	µM	Amino acid metabolism	Biocrates	**1,161**	**-0,0332**
Ala	120	µM	Amino acid metabolism	Biocrates	**0,439**	**-0,0125**
Ala	15	µM	Amino acid metabolism	Biocrates	**1,169**	**-0,0334**
Ala	240	µM	Amino acid metabolism	Biocrates	**0,936**	**-0,0268**
Ala	30	µM	Amino acid metabolism	Biocrates	**0,791**	**-0,0226**
Ala	60	µM	Amino acid metabolism	Biocrates	**1,148**	**-0,0328**
Ala	90	µM	Amino acid metabolism	Biocrates	**0,914**	**-0,0261**
Alkaline phosphatase	fasting	IU/L	General	Enzymatic	**0,643**	**-0,0184**
alpha-AAA	0	µM	Biogenic amine	Biocrates	**1,252**	**-0,0358**
alpha-AAA	120	µM	Biogenic amine	Biocrates	**1,132**	**-0,0324**
alpha-AAA	15	µM	Biogenic amine	Biocrates	**0,886**	**-0,0253**
alpha-AAA	240	µM	Biogenic amine	Biocrates	**0,805**	**-0,0230**
alpha-AAA	30	µM	Biogenic amine	Biocrates	**0,783**	**-0,0224**
alpha-AAA	60	µM	Biogenic amine	Biocrates	**0,777**	**-0,0222**
alpha-AAA	90	µM	Biogenic amine	Biocrates	**0,444**	**-0,0127**
Arg	0	µM	Amino acid metabolism	Biocrates	**0,987**	**0,0282**
Arg	120	µM	Amino acid metabolism	Biocrates	**0,303**	**0,0087**
Arg	15	µM	Amino acid metabolism	Biocrates	**0,094**	**0,0027**
Arg	240	µM	Amino acid metabolism	Biocrates	**0,033**	**0,0010**
Arg	30	µM	Amino acid metabolism	Biocrates	**0,731**	**0,0209**
Arg	60	µM	Amino acid metabolism	Biocrates	**0,343**	**-0,0098**
Arg	90	µM	Amino acid metabolism	Biocrates	**0,274**	**0,0078**
ASAT	fasting	%BW	Body composition	MRI	**0,926**	**0,0265**

Supplemental Table 1. Variables included in the PLS-DA model. Variables are presented with their respective VIP values and loadings, method of detection and units.

**Primary ID**	**Sampling time**	**Unit**	**Category**	**Method**	**VIP**	**Loadings**
ASAT	fasting	%TBF	Body composition	MRI	**1,451**	**0,0415**
ASAT	fasting	kg	Body composition	MRI	**0,265**	**0,0076**
Asn	0	µM	Amino acid metabolism	Biocrates	**1,083**	**0,0310**
Asn	120	µM	Amino acid metabolism	Biocrates	**0,653**	**0,0187**
Asn	15	µM	Amino acid metabolism	Biocrates	**0,970**	**0,0277**
Asn	240	µM	Amino acid metabolism	Biocrates	**0,823**	**0,0235**
Asn	30	µM	Amino acid metabolism	Biocrates	**1,543**	**0,0441**
Asn	60	µM	Amino acid metabolism	Biocrates	**0,069**	**0,0020**
Asn	90	µM	Amino acid metabolism	Biocrates	**0,533**	**0,0153**
Asp	0	µM	Amino acid metabolism	Biocrates	**0,543**	**-0,0155**
Asp	120	µM	Amino acid metabolism	Biocrates	**0,665**	**-0,0190**
Asp	15	µM	Amino acid metabolism	Biocrates	**0,610**	**-0,0174**
Asp	240	µM	Amino acid metabolism	Biocrates	**0,467**	**0,0134**
Asp	30	µM	Amino acid metabolism	Biocrates	**0,262**	**-0,0075**
Asp	60	µM	Amino acid metabolism	Biocrates	**0,462**	**0,0132**
Asp	90	µM	Amino acid metabolism	Biocrates	**0,075**	**-0,0021**
Aspartate aminotrans.	fasting	U/L	General	Enzymatic	**0,320**	**0,0092**
BA conj/unconj	0	ratio	Bile acid	LC-MS/MS	**0,878**	**0,0251**
BA conj/unconj	120	ratio	Bile acid	LC-MS/MS	**1,380**	**0,0395**
BA conj/unconj	15	ratio	Bile acid	LC-MS/MS	**1,269**	**0,0363**
BA conj/unconj	240	ratio	Bile acid	LC-MS/MS	**1,470**	**0,0420**
BA conj/unconj	30	ratio	Bile acid	LC-MS/MS	**0,743**	**0,0212**
BA conj/unconj	60	ratio	Bile acid	LC-MS/MS	**0,784**	**0,0224**
BA conj/unconj	90	ratio	Bile acid	LC-MS/MS	**1,132**	**0,0324**
BA prim/sec	0	ratio	Bile acid	LC-MS/MS	**0,315**	**0,0090**
BA prim/sec	120	ratio	Bile acid	LC-MS/MS	**0,200**	**-0,0057**
BA prim/sec	15	ratio	Bile acid	LC-MS/MS	**0,079**	**-0,0023**
BA prim/sec	240	ratio	Bile acid	LC-MS/MS	**0,288**	**-0,0082**
BA prim/sec	30	ratio	Bile acid	LC-MS/MS	**0,199**	**-0,0057**
BA prim/sec	60	ratio	Bile acid	LC-MS/MS	**0,173**	**-0,0049**
BA prim/sec	90	ratio	Bile acid	LC-MS/MS	**0,217**	**-0,0062**
BA primary	0	%	Bile acid	LC-MS/MS	**0,575**	**0,0165**
BA primary	120	%	Bile acid	LC-MS/MS	**0,514**	**0,0147**
BA primary	15	%	Bile acid	LC-MS/MS	**0,363**	**0,0104**
BA primary	240	%	Bile acid	LC-MS/MS	**0,638**	**0,0182**
BA primary	30	%	Bile acid	LC-MS/MS	**0,076**	**-0,0022**
BA primary	60	%	Bile acid	LC-MS/MS	**0,015**	**-0,0004**
BA primary	90	%	Bile acid	LC-MS/MS	**0,158**	**0,0045**
BA primary	0	nM	Bile acid	LC-MS/MS	**0,414**	**0,0118**
BA primary	120	nM	Bile acid	LC-MS/MS	**1,472**	**0,0421**
BA primary	15	nM	Bile acid	LC-MS/MS	**0,502**	**0,0144**
BA primary	240	nM	Bile acid	LC-MS/MS	**1,745**	**0,0499**
BA primary	30	nM	Bile acid	LC-MS/MS	**0,322**	**0,0092**
BA primary	60	nM	Bile acid	LC-MS/MS	**0,327**	**0,0094**
BA primary	90	nM	Bile acid	LC-MS/MS	**1,085**	**0,0310**
BA secondary	0	%	Bile acid	LC-MS/MS	**0,575**	**-0,0165**
BA secondary	120	%	Bile acid	LC-MS/MS	**0,514**	**-0,0147**
BA secondary	15	%	Bile acid	LC-MS/MS	**0,363**	**-0,0104**
BA secondary	240	%	Bile acid	LC-MS/MS	**0,638**	**-0,0182**
BA secondary	30	%	Bile acid	LC-MS/MS	**0,076**	**0,0022**
BA secondary	60	%	Bile acid	LC-MS/MS	**0,015**	**0,0004**
BA secondary	90	%	Bile acid	LC-MS/MS	**0,158**	**-0,0045**
BA secondary	0	nM	Bile acid	LC-MS/MS	**0,401**	**-0,0115**
BA secondary	120	nM	Bile acid	LC-MS/MS	**1,346**	**0,0385**
BA secondary	15	nM	Bile acid	LC-MS/MS	**0,381**	**-0,0109**
BA secondary	240	nM	Bile acid	LC-MS/MS	**1,535**	**0,0439**
BA secondary	30	nM	Bile acid	LC-MS/MS	**0,279**	**-0,0080**
BA secondary	60	nM	Bile acid	LC-MS/MS	**0,064**	**-0,0018**
BA secondary	90	nM	Bile acid	LC-MS/MS	**0,935**	**0,0267**
BA total	0	nM	Bile acid	LC-MS/MS	**0,076**	**0,0022**
BA total	120	nM	Bile acid	LC-MS/MS	**1,558**	**0,0446**
BA total	15	nM	Bile acid	LC-MS/MS	**0,218**	**0,0062**
BA total	240	nM	Bile acid	LC-MS/MS	**1,817**	**0,0519**
BA total	30	nM	Bile acid	LC-MS/MS	**0,127**	**0,0036**
BA total	60	nM	Bile acid	LC-MS/MS	**0,229**	**0,0066**
BA total	90	nM	Bile acid	LC-MS/MS	**1,105**	**0,0316**
BA unconj.	0	nM	Bile acid	LC-MS/MS	**0,328**	**-0,0094**
BA unconj.	120	nM	Bile acid	LC-MS/MS	**0,016**	**0,0005**
BA unconj.	15	nM	Bile acid	LC-MS/MS	**0,451**	**-0,0129**
BA unconj.	240	nM	Bile acid	LC-MS/MS	**0,104**	**0,0030**

Supplemental Table 1. Variables included in the PLS-DA model. Variables are presented with their respective VIP values and loadings, method of detection and units.

BA unconj.	30	nM	Bile acid	LC-MS/MS	**0,049**	**-0,0014**
BA unconj.	60	nM	Bile acid	LC-MS/MS	**0,049**	**0,0014**
**Primary ID**	**Sampling time**	**Unit**	**Category**	**Method**	**VIP**	**Loadings**
BA unconj.	90	nM	Bile acid	LC-MS/MS	**0,007**	**-0,0002**
BA unconjugated	0	%	Bile acid	LC-MS/MS	**1,035**	**-0,0296**
BA unconjugated	120	%	Bile acid	LC-MS/MS	**1,532**	**-0,0438**
BA unconjugated	15	%	Bile acid	LC-MS/MS	**0,795**	**-0,0227**
BA unconjugated	240	%	Bile acid	LC-MS/MS	**1,852**	**-0,0530**
BA unconjugated	30	%	Bile acid	LC-MS/MS	**0,223**	**-0,0064**
BA unconjugated	60	%	Bile acid	LC-MS/MS	**0,403**	**-0,0115**
BA unconjugated	90	%	Bile acid	LC-MS/MS	**1,007**	**-0,0288**
BMI	fasting	kg/m2	Body composition	Scale	**0,448**	**-0,0128**
C0	0	µM	Amino acid metabolism	Biocrates	**1,475**	**-0,0422**
C0	120	µM	Amino acid metabolism	Biocrates	**1,116**	**-0,0319**
C0	15	µM	Amino acid metabolism	Biocrates	**1,420**	**-0,0406**
C0	240	µM	Amino acid metabolism	Biocrates	**1,020**	**-0,0292**
C0	30	µM	Amino acid metabolism	Biocrates	**0,756**	**-0,0216**
C0	60	µM	Amino acid metabolism	Biocrates	**0,785**	**-0,0225**
C0	90	µM	Amino acid metabolism	Biocrates	**1,094**	**-0,0313**
C10	0	µM	Acylcarnitine	LC-MS/MS	**0,332**	**-0,0095**
C10	120	µM	Acylcarnitine	LC-MS/MS	**1,605**	**-0,0459**
C10	15	µM	Acylcarnitine	LC-MS/MS	**0,459**	**-0,0131**
C10	240	µM	Acylcarnitine	LC-MS/MS	**0,675**	**-0,0193**
C10	30	µM	Acylcarnitine	LC-MS/MS	**0,596**	**-0,0171**
C10	60	µM	Acylcarnitine	LC-MS/MS	**1,338**	**-0,0382**
C10	90	µM	Acylcarnitine	LC-MS/MS	**1,401**	**-0,0401**
C10:1	0	µM	Acylcarnitine	Biocrates	**0,550**	**0,0157**
C10:1	120	µM	Acylcarnitine	Biocrates	**0,535**	**0,0153**
C10:1	15	µM	Acylcarnitine	Biocrates	**0,013**	**-0,0004**
C10:1	240	µM	Acylcarnitine	Biocrates	**0,465**	**0,0133**
C10:1	30	µM	Acylcarnitine	Biocrates	**0,855**	**0,0245**
C10:1	60	µM	Acylcarnitine	Biocrates	**1,133**	**0,0324**
C10:1	90	µM	Acylcarnitine	Biocrates	**0,439**	**0,0126**
C10:2	0	µM	Acylcarnitine	Biocrates	**0,887**	**0,0254**
C10:2	120	µM	Acylcarnitine	Biocrates	**0,289**	**0,0083**
C10:2	15	µM	Acylcarnitine	Biocrates	**0,086**	**-0,0024**
C10:2	240	µM	Acylcarnitine	Biocrates	**0,475**	**0,0136**
C10:2	30	µM	Acylcarnitine	Biocrates	**0,943**	**0,0270**
C10:2	60	µM	Acylcarnitine	Biocrates	**1,177**	**0,0336**
C10:2	90	µM	Acylcarnitine	Biocrates	**0,082**	**-0,0023**
C12	0	µM	Acylcarnitine	LC-MS/MS	**0,408**	**-0,0117**
C12	120	µM	Acylcarnitine	LC-MS/MS	**1,663**	**-0,0475**
C12	15	µM	Acylcarnitine	LC-MS/MS	**0,608**	**-0,0174**
C12	240	µM	Acylcarnitine	LC-MS/MS	**0,791**	**-0,0226**
C12	30	µM	Acylcarnitine	LC-MS/MS	**0,913**	**-0,0261**
C12	60	µM	Acylcarnitine	LC-MS/MS	**1,376**	**-0,0394**
C12	90	µM	Acylcarnitine	LC-MS/MS	**1,600**	**-0,0457**
C12:0	fasting	%	Lipid metabolism	GC-FID	**1,326**	**-0,0379**
C12:0VITAS	fasting	µg/mL	Lipid metabolism	GC-FID	**1,440**	**-0,0412**
C12:1	0	µM	Acylcarnitine	Biocrates	**0,698**	**0,0200**
C12:1	120	µM	Acylcarnitine	Biocrates	**0,424**	**0,0121**
C12:1	15	µM	Acylcarnitine	Biocrates	**0,333**	**0,0095**
C12:1	240	µM	Acylcarnitine	Biocrates	**0,969**	**0,0277**
C12:1	30	µM	Acylcarnitine	Biocrates	**0,588**	**0,0168**
C12:1	60	µM	Acylcarnitine	Biocrates	**0,780**	**0,0223**
C12:1	90	µM	Acylcarnitine	Biocrates	**0,302**	**0,0086**
C12-DC	0	µM	Acylcarnitine	Biocrates	**0,950**	**0,0272**
C12-DC	120	µM	Acylcarnitine	Biocrates	**0,753**	**0,0215**
C12-DC	15	µM	Acylcarnitine	Biocrates	**0,418**	**0,0120**
C12-DC	240	µM	Acylcarnitine	Biocrates	**1,268**	**0,0363**
C12-DC	30	µM	Acylcarnitine	Biocrates	**0,979**	**0,0280**
C12-DC	60	µM	Acylcarnitine	Biocrates	**1,218**	**0,0348**
C12-DC	90	µM	Acylcarnitine	Biocrates	**0,368**	**0,0105**
C13	0	µM	Acylcarnitine	LC-MS/MS	**0,039**	**0,0011**
C13	120	µM	Acylcarnitine	LC-MS/MS	**0,043**	**-0,0012**
C13	15	µM	Acylcarnitine	LC-MS/MS	**0,067**	**0,0019**
C13	240	µM	Acylcarnitine	LC-MS/MS	**0,548**	**-0,0157**
C13	30	µM	Acylcarnitine	LC-MS/MS	**0,206**	**0,0059**
C13	60	µM	Acylcarnitine	LC-MS/MS	**0,206**	**0,0059**
C13	90	µM	Acylcarnitine	LC-MS/MS	**0,220**	**0,0063**
C14	0	µM	Acylcarnitine	LC-MS/MS	**1,141**	**-0,0326**

Supplemental Table 1. Variables included in the PLS-DA model. Variables are presented with their respective VIP values and loadings, method of detection and units.

Primary ID	Sampling time	Unit	Category	Method	VIP	Loadings
C14	120	µM	Acylcarnitine	LC-MS/MS	1,853	-0,0530
C14	15	µM	Acylcarnitine	LC-MS/MS	1,198	-0,0342
C14	240	µM	Acylcarnitine	LC-MS/MS	0,667	-0,0191
C14	30	µM	Acylcarnitine	LC-MS/MS	1,417	-0,0405
C14	60	µM	Acylcarnitine	LC-MS/MS	1,040	-0,0297
C14	90	µM	Acylcarnitine	LC-MS/MS	1,651	-0,0472
C14:0	fasting	%	Lipid metabolism	GC-FID	2,817	-0,0805
C14:0	fasting	µg/mL	Lipid metabolism	GC-FID	2,718	-0,0777
C14:1	0	µM	Acylcarnitine	Biocrates	0,418	-0,0120
C14:1	120	µM	Acylcarnitine	Biocrates	0,448	0,0128
C14:1	15	µM	Acylcarnitine	Biocrates	0,703	-0,0201
C14:1	240	µM	Acylcarnitine	Biocrates	0,386	-0,0111
C14:1	30	µM	Acylcarnitine	Biocrates	0,112	0,0032
C14:1	60	µM	Acylcarnitine	Biocrates	0,666	0,0190
C14:1	90	µM	Acylcarnitine	Biocrates	0,297	-0,0085
C14:1-OH	0	µM	Acylcarnitine	Biocrates	0,228	-0,0065
C14:1-OH	120	µM	Acylcarnitine	Biocrates	0,322	0,0092
C14:1-OH	15	µM	Acylcarnitine	Biocrates	1,058	-0,0303
C14:1-OH	240	µM	Acylcarnitine	Biocrates	0,431	-0,0123
C14:1-OH	30	µM	Acylcarnitine	Biocrates	0,345	0,0099
C14:1-OH	60	µM	Acylcarnitine	Biocrates	0,335	0,0096
C14:1-OH	90	µM	Acylcarnitine	Biocrates	0,236	-0,0067
C14:2	0	µM	Acylcarnitine	Biocrates	0,292	-0,0083
C14:2	120	µM	Acylcarnitine	Biocrates	0,555	0,0159
C14:2	15	µM	Acylcarnitine	Biocrates	0,768	-0,0220
C14:2	240	µM	Acylcarnitine	Biocrates	0,221	-0,0063
C14:2	30	µM	Acylcarnitine	Biocrates	0,501	0,0143
C14:2	60	µM	Acylcarnitine	Biocrates	0,639	0,0183
C14:2	90	µM	Acylcarnitine	Biocrates	0,056	-0,0016
C14:2-OH	0	µM	Acylcarnitine	Biocrates	0,044	0,0013
C14:2-OH	120	µM	Acylcarnitine	Biocrates	0,587	0,0168
C14:2-OH	15	µM	Acylcarnitine	Biocrates	0,296	-0,0085
C14:2-OH	240	µM	Acylcarnitine	Biocrates	0,129	-0,0037
C14:2-OH	30	µM	Acylcarnitine	Biocrates	0,484	0,0138
C14:2-OH	60	µM	Acylcarnitine	Biocrates	0,802	0,0229
C14:2-OH	90	µM	Acylcarnitine	Biocrates	0,057	-0,0016
C15:0	fasting	%	Lipid metabolism	GC-FID	0,634	-0,0181
C15:0	fasting	µg/mL	Lipid metabolism	GC-FID	1,929	-0,0551
C16	0	µM	Acylcarnitine	LC-MS/MS	1,401	-0,0401
C16	120	µM	Acylcarnitine	LC-MS/MS	1,529	-0,0437
C16	15	µM	Acylcarnitine	LC-MS/MS	1,486	-0,0425
C16	240	µM	Acylcarnitine	LC-MS/MS	0,551	-0,0157
C16	30	µM	Acylcarnitine	LC-MS/MS	1,297	-0,0371
C16	60	µM	Acylcarnitine	LC-MS/MS	1,140	-0,0326
C16	90	µM	Acylcarnitine	LC-MS/MS	1,445	-0,0413
C16:0	fasting	%	Lipid metabolism	GC-FID	3,038	-0,0869
C16:0	fasting	µg/mL	Lipid metabolism	GC-FID	2,541	-0,0727
C16:1	0	µM	Acylcarnitine	LC-MS/MS	0,718	-0,0205
C16:1	120	µM	Acylcarnitine	LC-MS/MS	1,557	-0,0445
C16:1	15	µM	Acylcarnitine	LC-MS/MS	0,669	-0,0191
C16:1	240	µM	Acylcarnitine	LC-MS/MS	0,030	0,0009
C16:1	30	µM	Acylcarnitine	LC-MS/MS	1,085	-0,0310
C16:1	60	µM	Acylcarnitine	LC-MS/MS	1,052	-0,0301
C16:1	90	µM	Acylcarnitine	LC-MS/MS	1,495	-0,0428
C16:1 n-7	fasting	µg/mL	Lipid metabolism	GC-FID	2,269	-0,0649
C16:1,n-7	fasting	%	Lipid metabolism	GC-FID	1,868	-0,0534
C16:1-OH	0	µM	Acylcarnitine	Biocrates	0,025	0,0007
C16:1-OH	120	µM	Acylcarnitine	Biocrates	0,632	0,0181
C16:1-OH	15	µM	Acylcarnitine	Biocrates	0,638	-0,0182
C16:1-OH	240	µM	Acylcarnitine	Biocrates	0,250	-0,0071
C16:1-OH	30	µM	Acylcarnitine	Biocrates	0,385	0,0110
C16:1-OH	60	µM	Acylcarnitine	Biocrates	0,841	0,0240
C16:1-OH	90	µM	Acylcarnitine	Biocrates	0,289	-0,0083
C16:2	0	µM	Acylcarnitine	Biocrates	0,561	-0,0161
C16:2	120	µM	Acylcarnitine	Biocrates	0,429	0,0123
C16:2	15	µM	Acylcarnitine	Biocrates	1,007	-0,0288
C16:2	240	µM	Acylcarnitine	Biocrates	0,565	-0,0162
C16:2	30	µM	Acylcarnitine	Biocrates	0,061	0,0017
C16:2	60	µM	Acylcarnitine	Biocrates	0,720	0,0206
C16:2	90	µM	Acylcarnitine	Biocrates	0,660	-0,0189

Supplemental Table 1. Variables included in the PLS-DA model. Variables are presented with their respective VIP values and loadings, method of detection and units.

Primary ID	Sampling time	Unit	Category	Method	VIP	Loadings
C16:2-OH	0	µM	Acylcarnitine	Biocrates	0,159	0,0046
C16:2-OH	120	µM	Acylcarnitine	Biocrates	0,738	0,0211
C16:2-OH	15	µM	Acylcarnitine	Biocrates	0,404	-0,0116
C16:2-OH	240	µM	Acylcarnitine	Biocrates	0,014	0,0004
C16:2-OH	30	µM	Acylcarnitine	Biocrates	0,638	0,0182
C16:2-OH	60	µM	Acylcarnitine	Biocrates	0,958	0,0274
C16:2-OH	90	µM	Acylcarnitine	Biocrates	0,162	-0,0046
C16-OH	0	µM	Acylcarnitine	Biocrates	0,010	-0,0003
C16-OH	120	µM	Acylcarnitine	Biocrates	0,714	0,0204
C16-OH	15	µM	Acylcarnitine	Biocrates	0,326	-0,0093
C16-OH	240	µM	Acylcarnitine	Biocrates	0,040	0,0012
C16-OH	30	µM	Acylcarnitine	Biocrates	0,347	0,0099
C16-OH	60	µM	Acylcarnitine	Biocrates	1,183	0,0338
C16-OH	90	µM	Acylcarnitine	Biocrates	0,218	0,0062
C18	0	µM	Acylcarnitine	LC-MS/MS	1,390	-0,0398
C18	120	µM	Acylcarnitine	LC-MS/MS	1,275	-0,0365
C18	15	µM	Acylcarnitine	LC-MS/MS	1,315	-0,0376
C18	240	µM	Acylcarnitine	LC-MS/MS	0,897	-0,0257
C18	30	µM	Acylcarnitine	LC-MS/MS	1,476	-0,0422
C18	60	µM	Acylcarnitine	LC-MS/MS	1,025	-0,0293
C18	90	µM	Acylcarnitine	LC-MS/MS	1,079	-0,0308
C18:0	fasting	%	Lipid metabolism	GC-FID	0,018	-0,0005
C18:0	fasting	µg/mL	Lipid metabolism	GC-FID	2,114	-0,0604
C18:1	0	µM	Acylcarnitine	LC-MS/MS	0,619	-0,0177
C18:1	120	µM	Acylcarnitine	LC-MS/MS	1,230	-0,0352
C18:1	15	µM	Acylcarnitine	LC-MS/MS	0,479	-0,0137
C18:1	240	µM	Acylcarnitine	LC-MS/MS	0,508	0,0145
C18:1	30	µM	Acylcarnitine	LC-MS/MS	1,012	-0,0290
C18:1	60	µM	Acylcarnitine	LC-MS/MS	0,645	-0,0184
C18:1	90	µM	Acylcarnitine	LC-MS/MS	0,769	-0,0220
C18:1 c11	fasting	µg/mL	Lipid metabolism	GC-FID	1,903	-0,0544
C18:1 c9	fasting	µg/mL	Lipid metabolism	GC-FID	2,336	-0,0668
C18:1 t6-11	fasting	µg/mL	Lipid metabolism	GC-FID	1,622	-0,0464
C18:1,c11	fasting	%	Lipid metabolism	GC-FID	0,766	-0,0219
C18:1,c9	fasting	%	Lipid metabolism	GC-FID	1,459	-0,0417
C18:1,t6-11	fasting	%	Lipid metabolism	GC-FID	0,643	-0,0184
C18:1-OH	0	µM	Acylcarnitine	Biocrates	0,018	0,0005
C18:1-OH	120	µM	Acylcarnitine	Biocrates	0,835	0,0239
C18:1-OH	15	µM	Acylcarnitine	Biocrates	0,715	-0,0204
C18:1-OH	240	µM	Acylcarnitine	Biocrates	0,242	-0,0069
C18:1-OH	30	µM	Acylcarnitine	Biocrates	0,228	0,0065
C18:1-OH	60	µM	Acylcarnitine	Biocrates	0,791	0,0226
C18:1-OH	90	µM	Acylcarnitine	Biocrates	0,273	-0,0078
C18:2	0	µM	Acylcarnitine	Biocrates	0,452	-0,0129
C18:2	120	µM	Acylcarnitine	Biocrates	0,545	0,0156
C18:2	15	µM	Acylcarnitine	Biocrates	0,384	-0,0110
C18:2	240	µM	Acylcarnitine	Biocrates	0,269	-0,0077
C18:2	30	µM	Acylcarnitine	Biocrates	0,318	0,0091
C18:2	60	µM	Acylcarnitine	Biocrates	0,835	0,0239
C18:2	90	µM	Acylcarnitine	Biocrates	0,015	0,0004
C18:2	0	µM	Acylcarnitine	LC-MS/MS	0,211	-0,0060
C18:2	120	µM	Acylcarnitine	LC-MS/MS	0,595	-0,0170
C18:2	15	µM	Acylcarnitine	LC-MS/MS	0,067	-0,0019
C18:2	240	µM	Acylcarnitine	LC-MS/MS	0,318	0,0091
C18:2	30	µM	Acylcarnitine	LC-MS/MS	0,524	-0,0150
C18:2	60	µM	Acylcarnitine	LC-MS/MS	0,081	-0,0023
C18:2	90	µM	Acylcarnitine	LC-MS/MS	0,114	-0,0033
C18:2 n-6	fasting	µg/mL	Lipid metabolism	GC-FID	0,361	-0,0103
C18:2,n-6	fasting	%	Lipid metabolism	GC-FID	2,514	0,0719
C18:3 n-3	fasting	µg/mL	Lipid metabolism	GC-FID	1,681	-0,0481
C18:3 n-6	fasting	µg/mL	Lipid metabolism	GC-FID	1,172	-0,0335
C18:3,n-3	fasting	%	Lipid metabolism	GC-FID	0,807	-0,0231
C18:3,n-6	fasting	%	Lipid metabolism	GC-FID	0,134	-0,0038
C2	0	µM	Acylcarnitine	LC-MS/MS	0,637	-0,0182
C2	120	µM	Acylcarnitine	LC-MS/MS	1,512	-0,0432
C2	15	µM	Acylcarnitine	LC-MS/MS	0,518	-0,0148
C2	240	µM	Acylcarnitine	LC-MS/MS	0,409	0,0117
C2	30	µM	Acylcarnitine	LC-MS/MS	0,733	-0,0210
C2	60	µM	Acylcarnitine	LC-MS/MS	1,047	-0,0299
C2	90	µM	Acylcarnitine	LC-MS/MS	1,263	-0,0361

Supplemental Table 1. Variables included in the PLS-DA model. Variables are presented with their respective VIP values and loadings, method of detection and units.

C20:0	fasting	%	Lipid metabolism	GC-FID	1,601	0,0458
C20:0	fasting	µg/mL	Lipid metabolism	GC-FID	0,540	-0,0154
C20:1 n-9	fasting	µg/mL	Lipid metabolism	GC-FID	1,670	-0,0477
C20:1,n-9	fasting	%	Lipid metabolism	GC-FID	0,071	-0,0020
C20:2 n-6	fasting	µg/mL	Lipid metabolism	GC-FID	1,692	-0,0484
**Primary ID**	**Sampling time**	**Unit**	**Category**	**Method**	**VIP**	**Loadings**
C20:2,n-6	fasting	%	Lipid metabolism	GC-FID	0,481	-0,0137
C20:3 n-6	fasting	µg/mL	Lipid metabolism	GC-FID	1,514	-0,0433
C20:3,n-6	fasting	%	Lipid metabolism	GC-FID	0,032	0,0009
C20:4 n-6	fasting	µg/mL	Lipid metabolism	GC-FID	0,644	-0,0184
C20:4,n-6	fasting	%	Lipid metabolism	GC-FID	1,351	0,0386
C20:5 n-3	fasting	µg/mL	Lipid metabolism	GC-FID	0,815	-0,0233
C20:5,n-3	fasting	%	Lipid metabolism	GC-FID	0,083	0,0024
C22:0	fasting	%	Lipid metabolism	GC-FID	2,036	0,0582
C22:0	fasting	µg/mL	Lipid metabolism	GC-FID	0,222	0,0063
C22:5 n-3	fasting	µg/mL	Lipid metabolism	GC-FID	2,351	-0,0672
C22:5,n-3	fasting	%	Lipid metabolism	GC-FID	0,908	-0,0260
C22:6 n-3	fasting	µg/mL	Lipid metabolism	GC-FID	1,369	-0,0392
C22:6,n-3	fasting	%	Lipid metabolism	GC-FID	0,132	-0,0038
C23:0	fasting	%	Lipid metabolism	GC-FID	1,960	0,0560
C23:0	fasting	µg/mL	Lipid metabolism	GC-FID	0,250	0,0072
C24:0	fasting	%	Lipid metabolism	GC-FID	1,842	0,0527
C24:0	fasting	µg/mL	Lipid metabolism	GC-FID	0,034	-0,0010
C24:1 n-9	fasting	µg/mL	Lipid metabolism	GC-FID	1,126	0,0322
C24:1,n-9	fasting	%	Lipid metabolism	GC-FID	2,738	0,0783
C3	0	µM	Acylcarnitine	LC-MS/MS	1,945	-0,0556
C3	120	µM	Acylcarnitine	LC-MS/MS	1,534	-0,0439
C3	15	µM	Acylcarnitine	LC-MS/MS	1,935	-0,0553
C3	240	µM	Acylcarnitine	LC-MS/MS	1,227	-0,0351
C3	30	µM	Acylcarnitine	LC-MS/MS	1,992	-0,0570
C3	60	µM	Acylcarnitine	LC-MS/MS	1,641	-0,0469
C3	90	µM	Acylcarnitine	LC-MS/MS	1,614	-0,0461
C3:1	0	µM	Acylcarnitine	Biocrates	0,226	0,0065
C3:1	120	µM	Acylcarnitine	Biocrates	0,659	0,0188
C3:1	15	µM	Acylcarnitine	Biocrates	0,148	-0,0042
C3:1	240	µM	Acylcarnitine	Biocrates	0,195	0,0056
C3:1	30	µM	Acylcarnitine	Biocrates	0,528	0,0151
C3:1	60	µM	Acylcarnitine	Biocrates	0,996	0,0285
C3:1	90	µM	Acylcarnitine	Biocrates	0,196	0,0056
C3-DC-M	0	µM	Acylcarnitine	LC-MS/MS	0,815	-0,0233
C3-DC-M	120	µM	Acylcarnitine	LC-MS/MS	0,862	-0,0246
C3-DC-M	15	µM	Acylcarnitine	LC-MS/MS	1,004	-0,0287
C3-DC-M	240	µM	Acylcarnitine	LC-MS/MS	1,246	-0,0356
C3-DC-M	30	µM	Acylcarnitine	LC-MS/MS	0,746	-0,0213
C3-DC-M	60	µM	Acylcarnitine	LC-MS/MS	1,072	-0,0306
C3-DC-M	90	µM	Acylcarnitine	LC-MS/MS	0,177	0,0051
C3-OH	0	µM	Acylcarnitine	Biocrates	0,036	0,0010
C3-OH	120	µM	Acylcarnitine	Biocrates	0,450	0,0129
C3-OH	15	µM	Acylcarnitine	Biocrates	0,602	-0,0172
C3-OH	240	µM	Acylcarnitine	Biocrates	0,080	-0,0023
C3-OH	30	µM	Acylcarnitine	Biocrates	0,124	0,0035
C3-OH	60	µM	Acylcarnitine	Biocrates	0,726	0,0208
C3-OH	90	µM	Acylcarnitine	Biocrates	0,134	-0,0038
C4	0	µM	Acylcarnitine	LC-MS/MS	1,161	-0,0332
C4	120	µM	Acylcarnitine	LC-MS/MS	1,710	-0,0489
C4	15	µM	Acylcarnitine	LC-MS/MS	1,213	-0,0347
C4	240	µM	Acylcarnitine	LC-MS/MS	1,328	-0,0380
C4	30	µM	Acylcarnitine	LC-MS/MS	1,391	-0,0398
C4	60	µM	Acylcarnitine	LC-MS/MS	1,510	-0,0432
C4	90	µM	Acylcarnitine	LC-MS/MS	1,616	-0,0462
C4:1	0	µM	Acylcarnitine	Biocrates	0,247	0,0071
C4:1	120	µM	Acylcarnitine	Biocrates	0,172	0,0049
C4:1	15	µM	Acylcarnitine	Biocrates	0,310	-0,0089
C4:1	240	µM	Acylcarnitine	Biocrates	0,158	0,0045
C4:1	30	µM	Acylcarnitine	Biocrates	0,122	0,0035
C4:1	60	µM	Acylcarnitine	Biocrates	0,546	0,0156
C4:1	90	µM	Acylcarnitine	Biocrates	0,246	-0,0070
C4-DC	0	µM	Acylcarnitine	LC-MS/MS	2,035	-0,0582
C4-DC	120	µM	Acylcarnitine	LC-MS/MS	1,462	-0,0418
C4-DC	15	µM	Acylcarnitine	LC-MS/MS	1,804	-0,0516
C4-DC	240	µM	Acylcarnitine	LC-MS/MS	1,447	-0,0414

Supplemental Table 1. Variables included in the PLS-DA model. Variables are presented with their respective VIP values and loadings, method of detection and units.

Primary ID	Sampling time	Unit	Category	Method	VIP	Loadings
C4-DC	30	µM	Acylcarnitine	LC-MS/MS	**1,813**	**-0,0518**
C4-DC	60	µM	Acylcarnitine	LC-MS/MS	**0,708**	**-0,0203**
C4-DC	90	µM	Acylcarnitine	LC-MS/MS	**0,063**	**-0,0018**
C4-OH - a	0	µM	Acylcarnitine	LC-MS/MS	**0,286**	**-0,0082**
C4-OH - a	120	µM	Acylcarnitine	LC-MS/MS	**0,787**	**-0,0225**
C4-OH - a	15	µM	Acylcarnitine	LC-MS/MS	**0,091**	**0,0026**
C4-OH - a	240	µM	Acylcarnitine	LC-MS/MS	**0,407**	**0,0117**
C4-OH - a	30	µM	Acylcarnitine	LC-MS/MS	**0,240**	**-0,0069**
C4-OH - a	60	µM	Acylcarnitine	LC-MS/MS	**0,118**	**-0,0034**
C4-OH - a	90	µM	Acylcarnitine	LC-MS/MS	**0,275**	**-0,0079**
C4-OH - b	0	µM	Acylcarnitine	LC-MS/MS	**0,432**	**-0,0123**
C4-OH - b	120	µM	Acylcarnitine	LC-MS/MS	**0,431**	**-0,0123**
C4-OH - b	15	µM	Acylcarnitine	LC-MS/MS	**0,155**	**-0,0044**
C4-OH - b	240	µM	Acylcarnitine	LC-MS/MS	**0,461**	**0,0132**
C4-OH - b	30	µM	Acylcarnitine	LC-MS/MS	**0,092**	**-0,0026**
C4-OH - b	60	µM	Acylcarnitine	LC-MS/MS	**0,232**	**-0,0066**
C4-OH - b	90	µM	Acylcarnitine	LC-MS/MS	**0,294**	**-0,0084**
C5	0	µM	Acylcarnitine	Biocrates	**0,597**	**-0,0171**
C5	120	µM	Acylcarnitine	Biocrates	**0,429**	**-0,0123**
C5	15	µM	Acylcarnitine	Biocrates	**1,151**	**-0,0329**
C5	240	µM	Acylcarnitine	Biocrates	**0,910**	**-0,0260**
C5	30	µM	Acylcarnitine	Biocrates	**0,944**	**-0,0270**
C5	60	µM	Acylcarnitine	Biocrates	**0,068**	**-0,0020**
C5	90	µM	Acylcarnitine	Biocrates	**0,801**	**-0,0229**
C5:1	0	µM	Acylcarnitine	Biocrates	**0,354**	**0,0101**
C5:1	120	µM	Acylcarnitine	Biocrates	**0,101**	**0,0029**
C5:1	15	µM	Acylcarnitine	Biocrates	**0,999**	**-0,0286**
C5:1	240	µM	Acylcarnitine	Biocrates	**0,019**	**-0,0005**
C5:1	30	µM	Acylcarnitine	Biocrates	**0,373**	**0,0107**
C5:1	60	µM	Acylcarnitine	Biocrates	**0,127**	**0,0036**
C5:1	90	µM	Acylcarnitine	Biocrates	**0,825**	**-0,0236**
C5:1-DC	0	µM	Acylcarnitine	Biocrates	**0,063**	**0,0018**
C5:1-DC	120	µM	Acylcarnitine	Biocrates	**0,344**	**0,0098**
C5:1-DC	15	µM	Acylcarnitine	Biocrates	**0,504**	**-0,0144**
C5:1-DC	240	µM	Acylcarnitine	Biocrates	**0,220**	**-0,0063**
C5:1-DC	30	µM	Acylcarnitine	Biocrates	**0,179**	**0,0051**
C5:1-DC	60	µM	Acylcarnitine	Biocrates	**0,660**	**0,0189**
C5:1-DC	90	µM	Acylcarnitine	Biocrates	**0,157**	**-0,0045**
C5-DC	0	µM	Acylcarnitine	LC-MS/MS	**0,680**	**-0,0194**
C5-DC	120	µM	Acylcarnitine	LC-MS/MS	**0,477**	**-0,0136**
C5-DC	15	µM	Acylcarnitine	LC-MS/MS	**0,663**	**-0,0190**
C5-DC	240	µM	Acylcarnitine	LC-MS/MS	**0,336**	**-0,0096**
C5-DC	30	µM	Acylcarnitine	LC-MS/MS	**0,615**	**-0,0176**
C5-DC	60	µM	Acylcarnitine	LC-MS/MS	**0,423**	**-0,0121**
C5-DC	90	µM	Acylcarnitine	LC-MS/MS	**0,262**	**-0,0075**
C5-M-DC	0	µM	Acylcarnitine	LC-MS/MS	**0,510**	**-0,0146**
C5-M-DC	120	µM	Acylcarnitine	LC-MS/MS	**0,428**	**-0,0123**
C5-M-DC	15	µM	Acylcarnitine	LC-MS/MS	**0,565**	**-0,0161**
C5-M-DC	240	µM	Acylcarnitine	LC-MS/MS	**0,481**	**-0,0137**
C5-M-DC	30	µM	Acylcarnitine	LC-MS/MS	**0,490**	**-0,0140**
C5-M-DC	60	µM	Acylcarnitine	LC-MS/MS	**0,439**	**-0,0126**
C5-M-DC	90	µM	Acylcarnitine	LC-MS/MS	**0,186**	**0,0053**
C5-OH	0	µM	Acylcarnitine	LC-MS/MS	**0,106**	**-0,0030**
C5-OH	120	µM	Acylcarnitine	LC-MS/MS	**0,304**	**-0,0087**
C5-OH	15	µM	Acylcarnitine	LC-MS/MS	**0,259**	**-0,0074**
C5-OH	240	µM	Acylcarnitine	LC-MS/MS	**0,474**	**-0,0136**
C5-OH	30	µM	Acylcarnitine	LC-MS/MS	**0,593**	**-0,0169**
C5-OH	60	µM	Acylcarnitine	LC-MS/MS	**0,398**	**-0,0114**
C5-OH	90	µM	Acylcarnitine	LC-MS/MS	**0,193**	**-0,0055**
C5-OH (C3-DC-M)	0	µM	Acylcarnitine	Biocrates	**0,085**	**-0,0024**
C5-OH (C3-DC-M)	120	µM	Acylcarnitine	Biocrates	**0,589**	**0,0168**
C5-OH (C3-DC-M)	15	µM	Acylcarnitine	Biocrates	**0,588**	**-0,0168**
C5-OH (C3-DC-M)	240	µM	Acylcarnitine	Biocrates	**0,163**	**0,0047**
C5-OH (C3-DC-M)	30	µM	Acylcarnitine	Biocrates	**0,059**	**0,0017**
C5-OH (C3-DC-M)	60	µM	Acylcarnitine	Biocrates	**0,782**	**0,0224**
C5-OH (C3-DC-M)	90	µM	Acylcarnitine	Biocrates	**0,321**	**0,0092**
C6	0	µM	Acylcarnitine	LC-MS/MS	**1,213**	**-0,0347**
C6	120	µM	Acylcarnitine	LC-MS/MS	**1,884**	**-0,0539**
C6	15	µM	Acylcarnitine	LC-MS/MS	**1,363**	**-0,0390**
C6	240	µM	Acylcarnitine	LC-MS/MS	**0,506**	**-0,0145**

Supplemental Table 1. Variables included in the PLS-DA model. Variables are presented with their respective VIP values and loadings, method of detection and units.

C6	30	µM	Acylcarnitine	LC-MS/MS	1,656	-0,0473
C6	60	µM	Acylcarnitine	LC-MS/MS	2,023	-0,0578
C6	90	µM	Acylcarnitine	LC-MS/MS	1,936	-0,0554
C6 (C4:1-DC)	0	µM	Acylcarnitine	LC-MS/MS	0,327	0,0094
C6 (C4:1-DC)	120	µM	Acylcarnitine	LC-MS/MS	0,256	0,0073
C6 (C4:1-DC)	15	µM	Acylcarnitine	LC-MS/MS	0,091	0,0026
C6 (C4:1-DC)	240	µM	Acylcarnitine	LC-MS/MS	0,435	0,0124
**Primary ID**	**Sampling time**	**Unit**	**Category**	**Method**	**VIP**	**Loadings**
C6 (C4:1-DC)	30	µM	Acylcarnitine	LC-MS/MS	0,458	0,0131
C6 (C4:1-DC)	60	µM	Acylcarnitine	LC-MS/MS	0,284	0,0081
C6 (C4:1-DC)	90	µM	Acylcarnitine	LC-MS/MS	0,314	-0,0090
C6:1	0	µM	Acylcarnitine	Biocrates	1,096	0,0313
C6:1	120	µM	Acylcarnitine	Biocrates	0,545	0,0156
C6:1	15	µM	Acylcarnitine	Biocrates	0,287	0,0082
C6:1	240	µM	Acylcarnitine	Biocrates	0,734	0,0210
C6:1	30	µM	Acylcarnitine	Biocrates	0,811	0,0232
C6:1	60	µM	Acylcarnitine	Biocrates	0,905	0,0259
C6:1	90	µM	Acylcarnitine	Biocrates	0,120	-0,0034
C6-DC	0	µM	Acylcarnitine	LC-MS/MS	0,920	-0,0263
C6-DC	120	µM	Acylcarnitine	LC-MS/MS	0,898	-0,0257
C6-DC	15	µM	Acylcarnitine	LC-MS/MS	0,964	-0,0276
C6-DC	240	µM	Acylcarnitine	LC-MS/MS	0,798	-0,0228
C6-DC	30	µM	Acylcarnitine	LC-MS/MS	1,063	-0,0304
C6-DC	60	µM	Acylcarnitine	LC-MS/MS	0,866	-0,0248
C6-DC	90	µM	Acylcarnitine	LC-MS/MS	1,015	-0,0290
C7-DC	0	µM	Acylcarnitine	LC-MS/MS	0,102	-0,0029
C7-DC	120	µM	Acylcarnitine	LC-MS/MS	0,001	0,0000
C7-DC	15	µM	Acylcarnitine	LC-MS/MS	0,221	-0,0063
C7-DC	240	µM	Acylcarnitine	LC-MS/MS	0,194	0,0056
C7-DC	30	µM	Acylcarnitine	LC-MS/MS	0,077	-0,0022
C7-DC	60	µM	Acylcarnitine	LC-MS/MS	0,069	-0,0020
C7-DC	90	µM	Acylcarnitine	LC-MS/MS	0,165	0,0047
C8	0	µM	Acylcarnitine	LC-MS/MS	0,569	-0,0163
C8	120	µM	Acylcarnitine	LC-MS/MS	1,644	-0,0470
C8	15	µM	Acylcarnitine	LC-MS/MS	0,778	-0,0222
C8	240	µM	Acylcarnitine	LC-MS/MS	0,759	-0,0217
C8	30	µM	Acylcarnitine	LC-MS/MS	0,756	-0,0216
C8	60	µM	Acylcarnitine	LC-MS/MS	1,511	-0,0432
C8	90	µM	Acylcarnitine	LC-MS/MS	1,456	-0,0416
C9	0	µM	Acylcarnitine	Biocrates	0,229	0,0065
C9	120	µM	Acylcarnitine	Biocrates	0,704	0,0201
C9	15	µM	Acylcarnitine	Biocrates	0,094	-0,0027
C9	240	µM	Acylcarnitine	Biocrates	0,106	0,0030
C9	30	µM	Acylcarnitine	Biocrates	0,557	0,0159
C9	60	µM	Acylcarnitine	Biocrates	0,975	0,0279
C9	90	µM	Acylcarnitine	Biocrates	0,272	0,0078
CA	0	µM	Bile acid	LC-MS/MS	0,396	0,0113
CA	120	µM	Bile acid	LC-MS/MS	0,160	0,0046
CA	15	µM	Bile acid	LC-MS/MS	0,072	-0,0021
CA	240	µM	Bile acid	LC-MS/MS	0,423	0,0121
CA	30	µM	Bile acid	LC-MS/MS	0,124	0,0035
CA	60	µM	Bile acid	LC-MS/MS	0,154	0,0044
CA	90	µM	Bile acid	LC-MS/MS	0,106	0,0030
CA	0	%	Bile acid	LC-MS/MS	0,070	0,0020
CA	120	%	Bile acid	LC-MS/MS	0,324	-0,0093
CA	15	%	Bile acid	LC-MS/MS	0,157	-0,0045
CA	240	%	Bile acid	LC-MS/MS	0,203	-0,0058
CA	30	%	Bile acid	LC-MS/MS	0,070	-0,0020
CA	60	%	Bile acid	LC-MS/MS	0,328	-0,0094
CA	90	%	Bile acid	LC-MS/MS	0,499	-0,0143
CDCA	0	µM	Bile acid	LC-MS/MS	0,146	-0,0042
CDCA	120	µM	Bile acid	LC-MS/MS	0,266	0,0076
CDCA	15	µM	Bile acid	LC-MS/MS	0,258	-0,0074
CDCA	240	µM	Bile acid	LC-MS/MS	0,258	0,0074
CDCA	30	µM	Bile acid	LC-MS/MS	0,053	0,0015
CDCA	60	µM	Bile acid	LC-MS/MS	0,191	0,0055
CDCA	90	µM	Bile acid	LC-MS/MS	0,197	0,0056
CDCA	0	%	Bile acid	LC-MS/MS	0,784	-0,0224
CDCA	120	%	Bile acid	LC-MS/MS	0,882	-0,0252
CDCA	15	%	Bile acid	LC-MS/MS	0,779	-0,0223
CDCA	240	%	Bile acid	LC-MS/MS	1,008	-0,0288

Supplemental Table 1. Variables included in the PLS-DA model. Variables are presented with their respective VIP values and loadings, method of detection and units.

CDCA	30	%	Bile acid	LC-MS/MS	**0,542**	**-0,0155**
CDCA	60	%	Bile acid	LC-MS/MS	**0,377**	**-0,0108**
CDCA	90	%	Bile acid	LC-MS/MS	**0,679**	**-0,0194**
Cholesterol	fasting	mM	Lipid metabolism	GC-MS	**0,259**	**0,0074**
Cholesterol	0	AU	Lipid metabolism	GC-MS	**1,578**	**-0,0451**
Cholesterol	120	AU	Lipid metabolism	GC-MS	**0,928**	**-0,0265**
Cholesterol	15	AU	Lipid metabolism	GC-MS	**0,931**	**-0,0266**
Cholesterol	240	AU	Lipid metabolism	GC-MS	**0,925**	**-0,0264**
**Primary ID**	**Sampling time**	**Unit**	**Category**	**Method**	**VIP**	**Loadings**
Cholesterol	30	AU	Lipid metabolism	GC-MS	**1,625**	**-0,0465**
Cholesterol	60	AU	Lipid metabolism	GC-MS	**1,439**	**-0,0411**
Cholesterol	90	AU	Lipid metabolism	GC-MS	**1,357**	**-0,0388**
Cit	0	µM	Amino acid metabolism	Biocrates	**1,020**	**-0,0292**
Cit	120	µM	Amino acid metabolism	Biocrates	**0,462**	**-0,0132**
Cit	15	µM	Amino acid metabolism	Biocrates	**0,768**	**-0,0219**
Cit	240	µM	Amino acid metabolism	Biocrates	**1,053**	**-0,0301**
Cit	30	µM	Amino acid metabolism	Biocrates	**0,269**	**-0,0077**
Cit	60	µM	Amino acid metabolism	Biocrates	**0,437**	**-0,0125**
Cit	90	µM	Amino acid metabolism	Biocrates	**0,233**	**-0,0067**
Creatinine	0	µM	Biogenic amine	Biocrates	**1,046**	**-0,0299**
Creatinine	120	µM	Biogenic amine	Biocrates	**0,897**	**-0,0257**
Creatinine	15	µM	Biogenic amine	Biocrates	**1,268**	**-0,0363**
Creatinine	240	µM	Biogenic amine	Biocrates	**1,599**	**-0,0457**
Creatinine	30	µM	Biogenic amine	Biocrates	**0,724**	**-0,0207**
Creatinine	60	µM	Biogenic amine	Biocrates	**0,608**	**-0,0174**
Creatinine	90	µM	Biogenic amine	Biocrates	**0,971**	**-0,0278**
CRP	fasting	ng/ml	Inflammation	Enzymatic	**0,761**	**0,0218**
DBP	fasting	mmHg	General	Sphygmomanometer	**0,322**	**-0,0092**
DCA	0	nM	Bile acid	LC-MS/MS	**0,743**	**-0,0212**
DCA	120	nM	Bile acid	LC-MS/MS	**0,613**	**-0,0175**
DCA	15	nM	Bile acid	LC-MS/MS	**1,151**	**-0,0329**
DCA	240	nM	Bile acid	LC-MS/MS	**0,525**	**-0,0150**
DCA	30	nM	Bile acid	LC-MS/MS	**0,646**	**-0,0185**
DCA	60	nM	Bile acid	LC-MS/MS	**0,500**	**-0,0143**
DCA	90	nM	Bile acid	LC-MS/MS	**0,527**	**-0,0151**
DCA	0	%	Bile acid	LC-MS/MS	**0,911**	**-0,0261**
DCA	120	%	Bile acid	LC-MS/MS	**1,667**	**-0,0477**
DCA	15	%	Bile acid	LC-MS/MS	**0,589**	**-0,0168**
DCA	240	%	Bile acid	LC-MS/MS	**2,245**	**-0,0642**
DCA	30	%	Bile acid	LC-MS/MS	**0,006**	**-0,0002**
DCA	60	%	Bile acid	LC-MS/MS	**0,300**	**-0,0086**
DCA	90	%	Bile acid	LC-MS/MS	**1,003**	**-0,0287**
Eosinophil	0	giga/l	Leucocyte	Cell counter	**0,120**	**-0,0034**
Eosinophil	120	giga/l	Leucocyte	Cell counter	**0,139**	**-0,0040**
Eosinophil	15	giga/l	Leucocyte	Cell counter	**0,033**	**0,0009**
Eosinophil	240	giga/l	Leucocyte	Cell counter	**0,110**	**-0,0031**
Eosinophil	30	giga/l	Leucocyte	Cell counter	**0,199**	**-0,0057**
Eosinophil	60	giga/l	Leucocyte	Cell counter	**0,321**	**0,0092**
Eosinophil	90	giga/l	Leucocyte	Cell counter	**0,315**	**0,0090**
Fructose	0	AU	Glucose metabolism	GC-MS	**1,006**	**-0,0288**
Fructose	120	AU	Glucose metabolism	GC-MS	**0,720**	**-0,0206**
Fructose	15	AU	Glucose metabolism	GC-MS	**0,834**	**-0,0238**
Fructose	240	AU	Glucose metabolism	GC-MS	**0,236**	**0,0067**
Fructose	30	AU	Glucose metabolism	GC-MS	**0,993**	**-0,0284**
Fructose	60	AU	Glucose metabolism	GC-MS	**0,531**	**-0,0152**
Fructose	90	AU	Glucose metabolism	GC-MS	**0,649**	**-0,0186**
Gamma-glutamyl transpeptidase	fasting	U/L	General	Enzymatic	**0,808**	**-0,0231**
GCA	0	nM	Bile acid	LC-MS/MS	**0,432**	**0,0124**
GCA	120	nM	Bile acid	LC-MS/MS	**1,365**	**0,0390**
GCA	15	nM	Bile acid	LC-MS/MS	**1,050**	**0,0300**
GCA	240	nM	Bile acid	LC-MS/MS	**1,481**	**0,0423**
GCA	30	nM	Bile acid	LC-MS/MS	**0,483**	**0,0138**
GCA	60	nM	Bile acid	LC-MS/MS	**0,564**	**0,0161**
GCA	90	nM	Bile acid	LC-MS/MS	**0,880**	**0,0252**
GCA	0	%	Bile acid	LC-MS/MS	**0,454**	**0,0130**
GCA	120	%	Bile acid	LC-MS/MS	**1,313**	**0,0376**
GCA	15	%	Bile acid	LC-MS/MS	**1,262**	**0,0361**
GCA	240	%	Bile acid	LC-MS/MS	**1,480**	**0,0423**
GCA	30	%	Bile acid	LC-MS/MS	**0,370**	**0,0106**
GCA	60	%	Bile acid	LC-MS/MS	**0,717**	**0,0205**
GCA	90	%	Bile acid	LC-MS/MS	**1,010**	**0,0289**

Supplemental Table 1. Variables included in the PLS-DA model. Variables are presented with their respective VIP values and loadings, method of detection and units.

GCDCA	0	nM	Bile acid	LC-MS/MS	**0,390**	**0,0111**
GCDCA	120	nM	Bile acid	LC-MS/MS	**1,804**	**0,0516**
GCDCA	15	nM	Bile acid	LC-MS/MS	**0,891**	**0,0255**
GCDCA	240	nM	Bile acid	LC-MS/MS	**2,244**	**0,0642**
GCDCA	30	nM	Bile acid	LC-MS/MS	**0,418**	**0,0120**
GCDCA	60	nM	Bile acid	LC-MS/MS	**0,163**	**0,0047**
GCDCA	90	nM	Bile acid	LC-MS/MS	**1,341**	**0,0383**
GCDCA	0	%	Bile acid	LC-MS/MS	**0,976**	**0,0279**
GCDCA	120	%	Bile acid	LC-MS/MS	**0,888**	**0,0254**
**Primary ID**	**Sampling time**	**Unit**	**Category**	**Method**	**VIP**	**Loadings**
GCDCA	15	%	Bile acid	LC-MS/MS	**1,065**	**0,0305**
GCDCA	240	%	Bile acid	LC-MS/MS	**0,953**	**0,0273**
GCDCA	30	%	Bile acid	LC-MS/MS	**0,610**	**0,0174**
GCDCA	60	%	Bile acid	LC-MS/MS	**0,377**	**0,0108**
GCDCA	90	%	Bile acid	LC-MS/MS	**0,649**	**0,0186**
GDCA	0	nM	Bile acid	LC-MS/MS	**0,141**	**0,0040**
GDCA	120	nM	Bile acid	LC-MS/MS	**1,721**	**0,0492**
GDCA	15	nM	Bile acid	LC-MS/MS	**0,029**	**0,0008**
GDCA	240	nM	Bile acid	LC-MS/MS	**1,897**	**0,0542**
GDCA	30	nM	Bile acid	LC-MS/MS	**0,432**	**-0,0124**
GDCA	60	nM	Bile acid	LC-MS/MS	**0,144**	**-0,0041**
GDCA	90	nM	Bile acid	LC-MS/MS	**1,024**	**0,0293**
GDCA	0	%	Bile acid	LC-MS/MS	**0,711**	**0,0203**
GDCA	120	%	Bile acid	LC-MS/MS	**0,984**	**0,0281**
GDCA	15	%	Bile acid	LC-MS/MS	**0,074**	**0,0021**
GDCA	240	%	Bile acid	LC-MS/MS	**1,192**	**0,0341**
GDCA	30	%	Bile acid	LC-MS/MS	**0,143**	**-0,0041**
GDCA	60	%	Bile acid	LC-MS/MS	**0,020**	**0,0006**
GDCA	90	%	Bile acid	LC-MS/MS	**0,622**	**0,0178**
Gln	0	µM	Amino acid metabolism	Biocrates	**1,026**	**0,0293**
Gln	120	µM	Amino acid metabolism	Biocrates	**1,720**	**0,0492**
Gln	15	µM	Amino acid metabolism	Biocrates	**1,568**	**0,0448**
Gln	240	µM	Amino acid metabolism	Biocrates	**1,254**	**0,0358**
Gln	30	µM	Amino acid metabolism	Biocrates	**2,172**	**0,0621**
Gln	60	µM	Amino acid metabolism	Biocrates	**1,495**	**0,0427**
Gln	90	µM	Amino acid metabolism	Biocrates	**1,268**	**0,0363**
GLP-1	0	pM	Signalling/ hormone	RIA	**0,538**	**0,0154**
GLP-1	120	pM	Signalling/ hormone	RIA	**1,012**	**0,0289**
GLP-1	15	pM	Signalling/ hormone	RIA	**0,074**	**-0,0021**
GLP-1	240	pM	Signalling/ hormone	RIA	**0,358**	**0,0102**
GLP-1	30	pM	Signalling/ hormone	RIA	**0,499**	**-0,0143**
GLP-1	60	pM	Signalling/ hormone	RIA	**0,606**	**0,0173**
GLP-1	90	pM	Signalling/ hormone	RIA	**1,030**	**0,0294**
Glu	0	µM	Amino acid metabolism	Biocrates	**2,490**	**-0,0712**
Glu	120	µM	Amino acid metabolism	Biocrates	**1,950**	**-0,0558**
Glu	15	µM	Amino acid metabolism	Biocrates	**2,471**	**-0,0707**
Glu	240	µM	Amino acid metabolism	Biocrates	**1,557**	**-0,0445**
Glu	30	µM	Amino acid metabolism	Biocrates	**2,592**	**-0,0741**
Glu	60	µM	Amino acid metabolism	Biocrates	**1,914**	**-0,0547**
Glu	90	µM	Amino acid metabolism	Biocrates	**2,215**	**-0,0633**
Glucagon	0	pg/ml	Signalling/ hormone	RIA	**0,857**	**-0,0245**
Glucagon	120	pg/ml	Signalling/ hormone	RIA	**0,837**	**-0,0239**
Glucagon	15	pg/ml	Signalling/ hormone	RIA	**1,105**	**-0,0316**
Glucagon	240	pg/ml	Signalling/ hormone	RIA	**0,102**	**-0,0029**
Glucagon	30	pg/ml	Signalling/ hormone	RIA	**1,111**	**-0,0318**
Glucagon	60	pg/ml	Signalling/ hormone	RIA	**1,008**	**-0,0288**
Glucagon	90	pg/ml	Signalling/ hormone	RIA	**0,623**	**-0,0178**
Gluconic acid	0	AU	Glucose metabolism	GC-MS	**0,019**	**0,0005**
Gluconic acid	120	AU	Glucose metabolism	GC-MS	**0,403**	**0,0115**
Gluconic acid	15	AU	Glucose metabolism	GC-MS	**0,567**	**-0,0162**
Gluconic acid	240	AU	Glucose metabolism	GC-MS	**0,479**	**-0,0137**
Gluconic acid	30	AU	Glucose metabolism	GC-MS	**1,371**	**-0,0392**
Gluconic acid	60	AU	Glucose metabolism	GC-MS	**1,455**	**-0,0416**
Gluconic acid	90	AU	Glucose metabolism	GC-MS	**0,459**	**0,0131**
**Glucose**	**0**	**mM**	**Glucose metabolism**	**Enzymatic**		
**Glucose**	**120**	**mM**	**Glucose metabolism**	**Enzymatic**		
**Glucose**	**15**	**mM**	**Glucose metabolism**	**Enzymatic**	**Not used in the PLS-DA model**	
**Glucose**	**240**	**mM**	**Glucose metabolism**	**Enzymatic**		
**Glucose**	**30**	**mM**	**Glucose metabolism**	**Enzymatic**		
**Glucose**	**60**	**mM**	**Glucose metabolism**	**Enzymatic**		
**Glucose**	**90**	**mM**	**Glucose metabolism**	**Enzymatic**		

Supplemental Table 1. Variables included in the PLS-DA model. Variables are presented with their respective VIP values and loadings, method of detection and units.

Primary ID	Sampling time	Unit	Category	Method	VIP	Loadings
Gly	0	µM	Amino acid metabolism	Biocrates	**1,508**	**0,0431**
Gly	120	µM	Amino acid metabolism	Biocrates	**1,676**	**0,0479**
Gly	15	µM	Amino acid metabolism	Biocrates	**1,598**	**0,0457**
Gly	240	µM	Amino acid metabolism	Biocrates	**1,485**	**0,0425**
Gly	30	µM	Amino acid metabolism	Biocrates	**1,937**	**0,0554**
Gly	60	µM	Amino acid metabolism	Biocrates	**1,756**	**0,0502**
Gly	90	µM	Amino acid metabolism	Biocrates	**1,875**	**0,0536**
Glycerol	0	AU	Lipid metabolism	GC-MS	**0,055**	**0,0016**
Glycerol	120	AU	Lipid metabolism	GC-MS	**0,769**	**-0,0220**
Glycerol	15	AU	Lipid metabolism	GC-MS	**0,084**	**-0,0024**
Glycerol	240	AU	Lipid metabolism	GC-MS	**0,411**	**0,0118**
Glycerol	30	AU	Lipid metabolism	GC-MS	**0,522**	**-0,0149**
Glycerol	60	AU	Lipid metabolism	GC-MS	**0,809**	**-0,0231**
Glycerol	90	AU	Lipid metabolism	GC-MS	**0,837**	**-0,0239**
Gly-conj BA	0	%	Bile acid	LC-MS/MS	**1,157**	**0,0331**
Gly-conj BA	120	%	Bile acid	LC-MS/MS	**1,867**	**0,0534**
Gly-conj BA	15	%	Bile acid	LC-MS/MS	**1,302**	**0,0372**
Gly-conj BA	240	%	Bile acid	LC-MS/MS	**2,208**	**0,0631**
Gly-conj BA	30	%	Bile acid	LC-MS/MS	**0,642**	**0,0184**
Gly-conj BA	60	%	Bile acid	LC-MS/MS	**0,776**	**0,0222**
Gly-conj BA	90	%	Bile acid	LC-MS/MS	**1,411**	**0,0403**
Gly-conj. BA	0	nM	Bile acid	LC-MS/MS	**0,395**	**0,0113**
Gly-conj. BA	120	nM	Bile acid	LC-MS/MS	**1,958**	**0,0560**
Gly-conj. BA	15	nM	Bile acid	LC-MS/MS	**0,867**	**0,0248**
Gly-conj. BA	240	nM	Bile acid	LC-MS/MS	**2,229**	**0,0637**
Gly-conj. BA	30	nM	Bile acid	LC-MS/MS	**0,339**	**0,0097**
Gly-conj. BA	60	nM	Bile acid	LC-MS/MS	**0,330**	**0,0094**
Gly-conj. BA	90	nM	Bile acid	LC-MS/MS	**1,292**	**0,0369**
GUDCA	0	nM	Bile acid	LC-MS/MS	**0,473**	**0,0135**
GUDCA	120	nM	Bile acid	LC-MS/MS	**1,481**	**0,0424**
GUDCA	15	nM	Bile acid	LC-MS/MS	**0,649**	**0,0186**
GUDCA	240	nM	Bile acid	LC-MS/MS	**1,918**	**0,0549**
GUDCA	30	nM	Bile acid	LC-MS/MS	**0,732**	**0,0209**
GUDCA	60	nM	Bile acid	LC-MS/MS	**0,719**	**0,0206**
GUDCA	90	nM	Bile acid	LC-MS/MS	**1,047**	**0,0299**
GUDCA	0	%	Bile acid	LC-MS/MS	**0,825**	**0,0236**
GUDCA	120	%	Bile acid	LC-MS/MS	**0,707**	**0,0202**
GUDCA	15	%	Bile acid	LC-MS/MS	**0,658**	**0,0188**
GUDCA	240	%	Bile acid	LC-MS/MS	**0,914**	**0,0261**
GUDCA	30	%	Bile acid	LC-MS/MS	**0,608**	**0,0174**
GUDCA	60	%	Bile acid	LC-MS/MS	**0,586**	**0,0167**
GUDCA	90	%	Bile acid	LC-MS/MS	**0,495**	**0,0141**
HbA1c	fasting	mmol/mol Hb	Glucose metabolism	Enzymatic	**1,886**	**-0,0539**
HDL-chol	fasting	mM	Lipid metabolism	Enzymatic	**1,219**	**0,0349**
Hip	fasting	cm	Body composition	Measuring tape	**0,326**	**0,0093**
His	0	µM	Amino acid metabolism	Biocrates	**0,543**	**0,0155**
His	120	µM	Amino acid metabolism	Biocrates	**0,541**	**0,0155**
His	15	µM	Amino acid metabolism	Biocrates	**0,168**	**0,0048**
His	240	µM	Amino acid metabolism	Biocrates	**0,559**	**0,0160**
His	30	µM	Amino acid metabolism	Biocrates	**1,442**	**0,0412**
His	60	µM	Amino acid metabolism	Biocrates	**0,503**	**0,0144**
His	90	µM	Amino acid metabolism	Biocrates	**0,782**	**0,0224**
Histamine	0	µM	Biogenic amine	Biocrates	**0,270**	**-0,0077**
Histamine	120	µM	Biogenic amine	Biocrates	**0,034**	**-0,0010**
Histamine	15	µM	Biogenic amine	Biocrates	**0,419**	**-0,0120**
Histamine	240	µM	Biogenic amine	Biocrates	**0,187**	**-0,0053**
Histamine	30	µM	Biogenic amine	Biocrates	**0,267**	**-0,0076**
Histamine	60	µM	Biogenic amine	Biocrates	**0,477**	**-0,0136**
Histamine	90	µM	Biogenic amine	Biocrates	**0,034**	**-0,0010**
IAAT	fasting	%BW	Body composition	MRI	**2,201**	**-0,0629**
IAAT	fasting	%TBF	Body composition	MRI	**1,971**	**-0,0564**
IAAT	fasting	kg	Body composition	MRI	**2,228**	**-0,0637**
IAAT:ASAT	fasting	ratio	Body composition	MRI	**2,054**	**-0,0587**
ICAM-1	0	ng/ml	Inflammation	ELISA	**0,633**	**-0,0181**
ICAM-1	120	ng/ml	Inflammation	ELISA	**0,697**	**-0,0199**
ICAM-1	60	ng/ml	Inflammation	ELISA	**0,370**	**-0,0106**
IL-10	0	pg/ml	Inflammation	ELISA	**0,545**	**0,0156**
IL-10	120	pg/ml	Inflammation	ELISA	**0,766**	**0,0219**
IL-10	60	pg/ml	Inflammation	ELISA	**0,779**	**0,0223**
IL-18	fasting	pg/ml	Inflammation	ELISA	**0,894**	**-0,0256**

Supplemental Table 1. Variables included in the PLS-DA model. Variables are presented with their respective VIP values and loadings, method of detection and units.

IL1-RA	0	pg/ml	Inflammation	ELISA	0,955	-0,0273
IL1-RA	120	pg/ml	Inflammation	ELISA	1,572	-0,0449
IL1-RA	60	pg/ml	Inflammation	ELISA	1,382	-0,0395
IL-8	0	pg/ml	Inflammation	ELISA	0,008	0,0002
IL-8	120	pg/ml	Inflammation	ELISA	0,551	0,0158
IL-8	60	pg/ml	Inflammation	ELISA	1,092	0,0312
Ile	0	µM	Amino acid metabolism	Biocrates	1,180	-0,0338
Ile	120	µM	Amino acid metabolism	Biocrates	1,584	-0,0453
Ile	15	µM	Amino acid metabolism	Biocrates	1,496	-0,0428
Ile	240	µM	Amino acid metabolism	Biocrates	0,472	-0,0135
Ile	30	µM	Amino acid metabolism	Biocrates	0,877	-0,0251
**Primary ID**	**Sampling time**	**Unit**	**Category**	**Method**	**VIP**	**Loadings**
Ile	60	µM	Amino acid metabolism	Biocrates	1,616	-0,0462
Ile	90	µM	Amino acid metabolism	Biocrates	1,663	-0,0476
**Insulin**	**0**	**mIU/l**	**Signalling/ hormone**	**RIA**		
**Insulin**	**120**	**mIU/l**	**Signalling/ hormone**	**RIA**		
**Insulin**	**15**	**mIU/l**	**Signalling/ hormone**	**RIA**		
**Insulin**	**240**	**mIU/l**	**Signalling/ hormone**	**RIA**	**Not used in the PLS-DA model**	
**Insulin**	**30**	**mIU/l**	**Signalling/ hormone**	**RIA**		
**Insulin**	**60**	**mIU/l**	**Signalling/ hormone**	**RIA**		
**Insulin**	**90**	**mIU/l**	**Signalling/ hormone**	**RIA**		
Intern.: subcut. fat	fasting	ratio	Body composition	MRI	1,953	-0,0559
Internal	fasting	%TBF	Body composition	MRI	1,975	-0,0565
Internal body fat	fasting	%BW	Body composition	MRI	2,167	-0,0620
Internal body fat	fasting	kg	Body composition	MRI	2,168	-0,0620
Kynurenine	0	µM	Biogenic amine	Biocrates	0,478	-0,0137
Kynurenine	120	µM	Biogenic amine	Biocrates	0,502	-0,0143
Kynurenine	15	µM	Biogenic amine	Biocrates	0,291	-0,0083
Kynurenine	240	µM	Biogenic amine	Biocrates	0,796	-0,0228
Kynurenine	30	µM	Biogenic amine	Biocrates	0,071	0,0020
Kynurenine	60	µM	Biogenic amine	Biocrates	0,170	-0,0049
Kynurenine	90	µM	Biogenic amine	Biocrates	0,210	-0,0060
Lactic acid	0	AU	Glucose metabolism	GC-MS	0,054	-0,0015
Lactic acid	120	AU	Glucose metabolism	GC-MS	0,782	-0,0224
Lactic acid	15	AU	Glucose metabolism	GC-MS	0,973	-0,0278
Lactic acid	240	AU	Glucose metabolism	GC-MS	0,554	-0,0158
Lactic acid	30	AU	Glucose metabolism	GC-MS	0,385	-0,0110
Lactic acid	60	AU	Glucose metabolism	GC-MS	1,300	-0,0372
Lactic acid	90	AU	Glucose metabolism	GC-MS	1,070	-0,0306
LDL-chol	fasting	mM	Lipid metabolism	Enzymatic	0,580	0,0166
Leptin	fasting	ng/ml	Signalling/ hormone	ELISA	0,362	-0,0104
Leu	0	µM	Amino acid metabolism	Biocrates	1,135	-0,0324
Leu	120	µM	Amino acid metabolism	Biocrates	1,508	-0,0431
Leu	15	µM	Amino acid metabolism	Biocrates	1,412	-0,0404
Leu	240	µM	Amino acid metabolism	Biocrates	0,729	-0,0208
Leu	30	µM	Amino acid metabolism	Biocrates	1,056	-0,0302
Leu	60	µM	Amino acid metabolism	Biocrates	1,616	-0,0462
Leu	90	µM	Amino acid metabolism	Biocrates	1,827	-0,0522
Linoleic acid	0	AU	Lipid metabolism	GC-MS	0,075	-0,0022
Linoleic acid	120	AU	Lipid metabolism	GC-MS	0,811	-0,0232
Linoleic acid	15	AU	Lipid metabolism	GC-MS	0,656	-0,0188
Linoleic acid	240	AU	Lipid metabolism	GC-MS	0,817	0,0233
Linoleic acid	30	AU	Lipid metabolism	GC-MS	0,781	-0,0223
Linoleic acid	60	AU	Lipid metabolism	GC-MS	1,126	-0,0322
Linoleic acid	90	AU	Lipid metabolism	GC-MS	0,708	-0,0202
Lipid hydroperoxides	fasting	ng/ml	Oxidative stress	x	0,125	0,0036
Liver fat	fasting	ratio	Body composition	MRI	2,830	-0,0809
Liver T2	fasting	ratio	Body composition	MRI	1,219	0,0349
Lymphocyte	0	giga/l	Leucocyte	Cell counter	1,073	-0,0307
Lymphocyte	120	giga/l	Leucocyte	Cell counter	0,413	-0,0118
Lymphocyte	15	giga/l	Leucocyte	Cell counter	1,108	-0,0317
Lymphocyte	240	giga/l	Leucocyte	Cell counter	1,594	-0,0456
Lymphocyte	30	giga/l	Leucocyte	Cell counter	1,242	-0,0355
Lymphocyte	60	giga/l	Leucocyte	Cell counter	0,761	-0,0217
Lymphocyte	90	giga/l	Leucocyte	Cell counter	0,400	-0,0114
Lys	0	µM	Amino acid metabolism	Biocrates	0,134	-0,0038
Lys	120	µM	Amino acid metabolism	Biocrates	0,257	-0,0074
Lys	15	µM	Amino acid metabolism	Biocrates	0,270	-0,0077
Lys	240	µM	Amino acid metabolism	Biocrates	0,340	-0,0097
Lys	30	µM	Amino acid metabolism	Biocrates	0,581	0,0166
Lys	60	µM	Amino acid metabolism	Biocrates	0,459	-0,0131

Supplemental Table 1. Variables included in the PLS-DA model. Variables are presented with their respective VIP values and loadings, method of detection and units.

Lys	90	µM	Amino acid metabolism	Biocrates	0,227	-0,0065
Mannose	0	AU	Glucose metabolism	GC-MS	0,791	-0,0226
Mannose	120	AU	Glucose metabolism	GC-MS	1,323	-0,0378
Mannose	15	AU	Glucose metabolism	GC-MS	0,379	-0,0108
Mannose	240	AU	Glucose metabolism	GC-MS	0,686	-0,0196
Mannose	30	AU	Glucose metabolism	GC-MS	0,833	-0,0238
Mannose	60	AU	Glucose metabolism	GC-MS	1,837	-0,0525
Mannose	90	AU	Glucose metabolism	GC-MS	1,740	-0,0497
MCP-1	0	pg/ml	Inflammation	ELISA	0,417	0,0119
MCP-1	120	pg/ml	Inflammation	ELISA	0,477	0,0136
MCP-1	60	pg/ml	Inflammation	ELISA	0,552	0,0158
MDA	fasting	µM	Oxidative stress	x	1,567	-0,0448
**Primary ID**	**Sampling time**	**Unit**	**Category**	**Method**	**VIP**	**Loadings**
meso-Erythritol	0	AU	Glucose metabolism	GC-MS	1,405	-0,0402
meso-Erythritol	120	AU	Glucose metabolism	GC-MS	1,077	-0,0308
meso-Erythritol	15	AU	Glucose metabolism	GC-MS	1,161	-0,0332
meso-Erythritol	240	AU	Glucose metabolism	GC-MS	0,893	-0,0255
meso-Erythritol	30	AU	Glucose metabolism	GC-MS	1,296	-0,0370
meso-Erythritol	60	AU	Glucose metabolism	GC-MS	1,737	-0,0497
meso-Erythritol	90	AU	Glucose metabolism	GC-MS	1,348	-0,0386
Met	0	µM	Amino acid metabolism	Biocrates	0,314	-0,0090
Met	120	µM	Amino acid metabolism	Biocrates	0,636	-0,0182
Met	15	µM	Amino acid metabolism	Biocrates	0,557	-0,0159
Met	240	µM	Amino acid metabolism	Biocrates	0,042	-0,0012
Met	30	µM	Amino acid metabolism	Biocrates	0,277	0,0079
Met	60	µM	Amino acid metabolism	Biocrates	0,564	-0,0161
Met	90	µM	Amino acid metabolism	Biocrates	0,582	-0,0166
Met-SO	0	µM	Amino acid metabolism	Biocrates	0,051	0,0015
Met-SO	120	µM	Amino acid metabolism	Biocrates	0,088	-0,0025
Met-SO	15	µM	Amino acid metabolism	Biocrates	0,341	-0,0098
Met-SO	240	µM	Amino acid metabolism	Biocrates	0,051	0,0015
Met-SO	30	µM	Amino acid metabolism	Biocrates	0,099	-0,0028
Met-SO	60	µM	Amino acid metabolism	Biocrates	0,232	0,0066
Met-SO	90	µM	Amino acid metabolism	Biocrates	0,046	0,0013
Monocyte count	0	giga/l	Leucocyte	Cell counter	1,615	-0,0462
Monocyte count	120	giga/l	Leucocyte	Cell counter	0,930	-0,0266
Monocyte count	15	giga/l	Leucocyte	Cell counter	2,078	-0,0594
Monocyte count	240	giga/l	Leucocyte	Cell counter	2,579	-0,0737
Monocyte count	30	giga/l	Leucocyte	Cell counter	1,857	-0,0531
Monocyte count	60	giga/l	Leucocyte	Cell counter	1,238	-0,0354
Monocyte count	90	giga/l	Leucocyte	Cell counter	0,711	-0,0203
Myo-inositol	0	AU	Glucose metabolism	GC-MS	1,041	-0,0298
Myo-inositol	120	AU	Glucose metabolism	GC-MS	0,284	-0,0081
Myo-inositol	15	AU	Glucose metabolism	GC-MS	0,060	-0,0017
Myo-inositol	240	AU	Glucose metabolism	GC-MS	0,499	0,0143
Myo-inositol	30	AU	Glucose metabolism	GC-MS	0,558	-0,0160
Myo-inositol	60	AU	Glucose metabolism	GC-MS	0,533	-0,0153
Myo-inositol	90	AU	Glucose metabolism	GC-MS	0,424	-0,0121
NAIAT	fasting	%BW	Body composition	MRI	1,271	-0,0363
NAIAT	fasting	%TBF	Body composition	MRI	1,570	-0,0449
NAIAT (kg)	fasting	kg	Body composition	MRI	1,561	-0,0446
NASAT	fasting	%BW	Body composition	MRI	1,042	0,0298
NASAT	fasting	%TBF	Body composition	MRI	1,710	0,0489
NASAT (kg)	fasting	kg	Body composition	MRI	0,357	0,0102
NEFA	0	mM	Lipid metabolism	Enzymatic	0,907	-0,0259
NEFA	120	mM	Lipid metabolism	Enzymatic	1,359	-0,0389
NEFA	15	mM	Lipid metabolism	Enzymatic	1,113	-0,0318
NEFA	240	mM	Lipid metabolism	Enzymatic	0,590	0,0169
NEFA	30	mM	Lipid metabolism	Enzymatic	1,857	-0,0531
NEFA	60	mM	Lipid metabolism	Enzymatic	2,406	-0,0688
NEFA	90	mM	Lipid metabolism	Enzymatic	1,864	-0,0533
Neutrophil count	0	giga/l	Leucocyte	Cell counter	1,473	-0,0421
Neutrophil count	120	giga/l	Leucocyte	Cell counter	1,315	-0,0376
Neutrophil count	15	giga/l	Leucocyte	Cell counter	1,629	-0,0466
Neutrophil count	240	giga/l	Leucocyte	Cell counter	1,637	-0,0468
Neutrophil count	30	giga/l	Leucocyte	Cell counter	1,503	-0,0430
Neutrophil count	60	giga/l	Leucocyte	Cell counter	1,381	-0,0395
Neutrophil count	90	giga/l	Leucocyte	Cell counter	1,231	-0,0352
Oleic acid	0	AU	Lipid metabolism	GC-MS	0,810	-0,0232
Oleic acid	120	AU	Lipid metabolism	GC-MS	1,681	-0,0481
Oleic acid	15	AU	Lipid metabolism	GC-MS	0,897	-0,0257

Supplemental Table 1. Variables included in the PLS-DA model. Variables are presented with their respective VIP values and loadings, method of detection and units.

Oleic acid	240	AU	Lipid metabolism	GC-MS	0,827	0,0237
Oleic acid	30	AU	Lipid metabolism	GC-MS	2,078	-0,0594
Oleic acid	60	AU	Lipid metabolism	GC-MS	2,112	-0,0604
Oleic acid	90	AU	Lipid metabolism	GC-MS	2,061	-0,0589
Orn	0	µM	Biogenic amine	Biocrates	0,030	0,0009
Orn	120	µM	Biogenic amine	Biocrates	0,146	0,0042
Orn	15	µM	Biogenic amine	Biocrates	0,713	0,0204
Orn	240	µM	Biogenic amine	Biocrates	0,488	0,0139
Orn	30	µM	Biogenic amine	Biocrates	0,949	0,0271
Orn	60	µM	Biogenic amine	Biocrates	0,721	0,0206
Orn	90	µM	Biogenic amine	Biocrates	1,034	0,0296
Palmitic acid	0	AU	Lipid metabolism	GC-MS	1,368	-0,0391
Palmitic acid	120	AU	Lipid metabolism	GC-MS	1,685	-0,0482
**Primary ID**	**Sampling time**	**Unit**	**Category**	**Method**	**VIP**	**Loadings**
Palmitic acid	15	AU	Lipid metabolism	GC-MS	1,133	-0,0324
Palmitic acid	240	AU	Lipid metabolism	GC-MS	0,065	0,0019
Palmitic acid	30	AU	Lipid metabolism	GC-MS	1,647	-0,0471
Palmitic acid	60	AU	Lipid metabolism	GC-MS	2,001	-0,0572
Palmitic acid	90	AU	Lipid metabolism	GC-MS	1,920	-0,0549
Pancreas fat	fasting	ratio	Body composition	MRI	0,069	-0,0020
Phe	0	µM	Amino acid metabolism	Biocrates	1,101	-0,0315
Phe	120	µM	Amino acid metabolism	Biocrates	1,360	-0,0389
Phe	15	µM	Amino acid metabolism	Biocrates	1,566	-0,0448
Phe	240	µM	Amino acid metabolism	Biocrates	0,735	-0,0210
Phe	30	µM	Amino acid metabolism	Biocrates	0,581	-0,0166
Phe	60	µM	Amino acid metabolism	Biocrates	1,364	-0,0390
Phe	90	µM	Amino acid metabolism	Biocrates	1,514	-0,0433
Pro	0	µM	Amino acid metabolism	Biocrates	0,686	-0,0196
Pro	120	µM	Amino acid metabolism	Biocrates	1,077	-0,0308
Pro	15	µM	Amino acid metabolism	Biocrates	1,023	-0,0293
Pro	240	µM	Amino acid metabolism	Biocrates	0,705	-0,0201
Pro	30	µM	Amino acid metabolism	Biocrates	0,439	-0,0126
Pro	60	µM	Amino acid metabolism	Biocrates	0,990	-0,0283
Pro	90	µM	Amino acid metabolism	Biocrates	1,081	-0,0309
Putrescine	0	µM	Biogenic amine	Biocrates	0,294	-0,0084
Putrescine	120	µM	Biogenic amine	Biocrates	0,236	0,0068
Putrescine	15	µM	Biogenic amine	Biocrates	0,154	0,0044
Putrescine	240	µM	Biogenic amine	Biocrates	0,420	0,0120
Putrescine	30	µM	Biogenic amine	Biocrates	0,190	-0,0054
Putrescine	60	µM	Biogenic amine	Biocrates	0,046	-0,0013
Putrescine	90	µM	Biogenic amine	Biocrates	0,624	0,0178
Pyruvate	0	AU	Glucose metabolism	GC-MS	0,861	-0,0246
Pyruvate	120	AU	Glucose metabolism	GC-MS	0,542	-0,0155
Pyruvate	15	AU	Glucose metabolism	GC-MS	0,420	-0,0120
Pyruvate	240	AU	Glucose metabolism	GC-MS	0,571	-0,0163
Pyruvate	30	AU	Glucose metabolism	GC-MS	1,413	-0,0404
Pyruvate	60	AU	Glucose metabolism	GC-MS	1,404	-0,0401
Pyruvate	90	AU	Glucose metabolism	GC-MS	1,646	-0,0471
PYY	0	pM	Signalling/ hormone	RIA	0,954	-0,0273
PYY	120	pM	Signalling/ hormone	RIA	0,003	0,0001
PYY	15	pM	Signalling/ hormone	RIA	0,952	-0,0272
PYY	240	pM	Signalling/ hormone	RIA	0,702	-0,0201
PYY	30	pM	Signalling/ hormone	RIA	0,365	-0,0104
PYY	60	pM	Signalling/ hormone	RIA	0,769	-0,0220
PYY	90	pM	Signalling/ hormone	RIA	0,612	-0,0175
Ribitol	0	AU	Glucose metabolism	GC-MS	0,457	-0,0131
Ribitol	120	AU	Glucose metabolism	GC-MS	0,665	-0,0190
Ribitol	15	AU	Glucose metabolism	GC-MS	0,519	-0,0148
Ribitol	240	AU	Glucose metabolism	GC-MS	0,253	-0,0072
Ribitol	30	AU	Glucose metabolism	GC-MS	0,357	-0,0102
Ribitol	60	AU	Glucose metabolism	GC-MS	0,431	-0,0123
Ribitol	90	AU	Glucose metabolism	GC-MS	0,925	-0,0264
SAT	fasting	%BW	Body composition	MRI	1	0,0294602
SAT	fasting	%TBF	Body composition	MRI	1,975	0,0565
SAT	fasting	kg	Body composition	MRI	0,333	0,0095
SBP	fasting	mmHg	General	Sphygmomanometer	0,008	0,0002
SDMA	0	µM	Biogenic amine	Biocrates	0,110	0,0031
SDMA	120	µM	Biogenic amine	Biocrates	1,145	0,0327
SDMA	15	µM	Biogenic amine	Biocrates	0,624	-0,0179
SDMA	240	µM	Biogenic amine	Biocrates	0,552	0,0158
SDMA	30	µM	Biogenic amine	Biocrates	0,804	-0,0230

Supplemental Table 1. Variables included in the PLS-DA model. Variables are presented with their respective VIP values and loadings, method of detection and units.

SDMA	60	µM	Biogenic amine	Biocrates	**0,273**	**0,0078**
SDMA	90	µM	Biogenic amine	Biocrates	**0,534**	**0,0153**
Ser	0	µM	Amino acid metabolism	Biocrates	**1,424**	**0,0407**
Ser	120	µM	Amino acid metabolism	Biocrates	**1,108**	**0,0317**
Ser	15	µM	Amino acid metabolism	Biocrates	**1,216**	**0,0348**
Ser	240	µM	Amino acid metabolism	Biocrates	**1,372**	**0,0392**
Ser	30	µM	Amino acid metabolism	Biocrates	**1,946**	**0,0556**
Ser	60	µM	Amino acid metabolism	Biocrates	**1,129**	**0,0323**
Ser	90	µM	Amino acid metabolism	Biocrates	**1,135**	**0,0325**
Serotonin	0	µM	Biogenic amine	Biocrates	**0,056**	**-0,0016**
Serotonin	120	µM	Biogenic amine	Biocrates	**0,882**	**0,0252**
Serotonin	15	µM	Biogenic amine	Biocrates	**0,045**	**-0,0013**
Serotonin	240	µM	Biogenic amine	Biocrates	**0,548**	**0,0157**
Serotonin	30	µM	Biogenic amine	Biocrates	**0,779**	**-0,0223**
**Primary ID**	**Sampling time**	**Unit**	**Category**	**Method**	**VIP**	**Loadings**
Serotonin	60	µM	Biogenic amine	Biocrates	**0,491**	**0,0140**
Serotonin	90	µM	Biogenic amine	Biocrates	**0,775**	**0,0222**
s-E-Selectin	fasting	ng/ml	Inflammation	ELISA	**1,551**	**-0,0444**
Soleus-IMCL	fasting	ratio	Body composition	MRI	**1,381**	**-0,0395**
Spermidine	0	µM	Biogenic amine	Biocrates	**1,267**	**-0,0362**
Spermidine	120	µM	Biogenic amine	Biocrates	**1,124**	**-0,0321**
Spermidine	15	µM	Biogenic amine	Biocrates	**1,267**	**-0,0362**
Spermidine	240	µM	Biogenic amine	Biocrates	**1,107**	**-0,0317**
Spermidine	30	µM	Biogenic amine	Biocrates	**1,421**	**-0,0406**
Spermidine	60	µM	Biogenic amine	Biocrates	**1,068**	**-0,0305**
Spermidine	90	µM	Biogenic amine	Biocrates	**0,588**	**-0,0168**
Spermine	0	µM	Biogenic amine	Biocrates	**1,477**	**-0,0422**
Spermine	120	µM	Biogenic amine	Biocrates	**1,359**	**-0,0389**
Spermine	15	µM	Biogenic amine	Biocrates	**1,239**	**-0,0354**
Spermine	240	µM	Biogenic amine	Biocrates	**1,332**	**-0,0381**
Spermine	30	µM	Biogenic amine	Biocrates	**1,405**	**-0,0402**
Spermine	60	µM	Biogenic amine	Biocrates	**1,167**	**-0,0334**
Spermine	90	µM	Biogenic amine	Biocrates	**1,174**	**-0,0336**
Stearic acid	0	AU	Lipid metabolism	GC-MS	**1,729**	**-0,0494**
Stearic acid	120	AU	Lipid metabolism	GC-MS	**1,976**	**-0,0565**
Stearic acid	15	AU	Lipid metabolism	GC-MS	**1,209**	**-0,0346**
Stearic acid	240	AU	Lipid metabolism	GC-MS	**0,246**	**-0,0070**
Stearic acid	30	AU	Lipid metabolism	GC-MS	**2,558**	**-0,0731**
Stearic acid	60	AU	Lipid metabolism	GC-MS	**2,360**	**-0,0675**
Stearic acid	90	AU	Lipid metabolism	GC-MS	**1,568**	**-0,0448**
Sum CA	0	nM	Bile acid	LC-MS/MS	**0,572**	**0,0163**
Sum CA	120	nM	Bile acid	LC-MS/MS	**1,142**	**0,0327**
Sum CA	15	nM	Bile acid	LC-MS/MS	**0,430**	**0,0123**
Sum CA	240	nM	Bile acid	LC-MS/MS	**1,277**	**0,0365**
Sum CA	30	nM	Bile acid	LC-MS/MS	**0,386**	**0,0110**
Sum CA	60	nM	Bile acid	LC-MS/MS	**0,488**	**0,0139**
Sum CA	90	nM	Bile acid	LC-MS/MS	**0,783**	**0,0224**
Sum CDCA	0	nM	Bile acid	LC-MS/MS	**0,230**	**0,0066**
Sum CDCA	120	nM	Bile acid	LC-MS/MS	**1,553**	**0,0444**
Sum CDCA	15	nM	Bile acid	LC-MS/MS	**0,495**	**0,0142**
Sum CDCA	240	nM	Bile acid	LC-MS/MS	**1,960**	**0,0561**
Sum CDCA	30	nM	Bile acid	LC-MS/MS	**0,220**	**0,0063**
Sum CDCA	60	nM	Bile acid	LC-MS/MS	**0,146**	**0,0042**
Sum CDCA	90	nM	Bile acid	LC-MS/MS	**1,203**	**0,0344**
Sum DCA	0	nM	Bile acid	LC-MS/MS	**0,470**	**-0,0134**
Sum DCA	120	nM	Bile acid	LC-MS/MS	**1,064**	**0,0304**
Sum DCA	15	nM	Bile acid	LC-MS/MS	**0,619**	**-0,0177**
Sum DCA	240	nM	Bile acid	LC-MS/MS	**1,276**	**0,0365**
Sum DCA	30	nM	Bile acid	LC-MS/MS	**0,574**	**-0,0164**
Sum DCA	60	nM	Bile acid	LC-MS/MS	**0,272**	**-0,0078**
Sum DCA	90	nM	Bile acid	LC-MS/MS	**0,754**	**0,0216**
Sum lyso PC	0	µM	Glycerophopspholipid	Biocrates	**0,630**	**0,0180**
Sum lyso PC	120	µM	Glycerophopspholipid	Biocrates	**0,666**	**0,0190**
Sum lyso PC	15	µM	Glycerophopspholipid	Biocrates	**0,303**	**0,0087**
Sum lyso PC	240	µM	Glycerophopspholipid	Biocrates	**0,502**	**0,0143**
Sum lyso PC	30	µM	Glycerophopspholipid	Biocrates	**0,972**	**0,0278**
Sum lyso PC	60	µM	Glycerophopspholipid	Biocrates	**1,170**	**0,0334**
Sum lyso PC	90	µM	Glycerophopspholipid	Biocrates	**0,467**	**0,0134**
Sum PC aa	0	µM	Glycerophopspholipid	Biocrates	**0,131**	**0,0037**
Sum PC aa	120	µM	Glycerophopspholipid	Biocrates	**0,097**	**-0,0028**
Sum PC aa	15	µM	Glycerophopspholipid	Biocrates	**0,036**	**0,0010**

Supplemental Table 1. Variables included in the PLS-DA model. Variables are presented with their respective VIP values and loadings, method of detection and units.

Sum PC aa	240	µM	Glycerophopspholipid	Biocrates	**0,347**	**0,0099**
Sum PC aa	30	µM	Glycerophopspholipid	Biocrates	**0,421**	**0,0120**
Sum PC aa	60	µM	Glycerophopspholipid	Biocrates	**0,826**	**0,0236**
Sum PC aa	90	µM	Glycerophopspholipid	Biocrates	**0,357**	**0,0102**
Sum PC ae	0	µM	Glycerophopspholipid	Biocrates	**0,575**	**0,0164**
Sum PC ae	120	µM	Glycerophopspholipid	Biocrates	**0,564**	**0,0161**
Sum PC ae	15	µM	Glycerophopspholipid	Biocrates	**0,597**	**0,0171**
Sum PC ae	240	µM	Glycerophopspholipid	Biocrates	**1,013**	**0,0290**
Sum PC ae	30	µM	Glycerophopspholipid	Biocrates	**0,982**	**0,0281**
Sum PC ae	60	µM	Glycerophopspholipid	Biocrates	**1,314**	**0,0376**
Sum PC ae	90	µM	Glycerophopspholipid	Biocrates	**0,940**	**0,0269**
Sum sphingomyelins	0	µM	Sphingomyelin	Biocrates	**0,490**	**0,0140**
Sum sphingomyelins	120	µM	Sphingomyelin	Biocrates	**0,598**	**0,0171**
Sum sphingomyelins	15	µM	Sphingomyelin	Biocrates	**0,531**	**0,0152**
Sum sphingomyelins	240	µM	Sphingomyelin	Biocrates	**0,856**	**0,0245**
**Primary ID**	**Sampling time**	**Unit**	**Category**	**Method**	**VIP**	**Loadings**
Sum sphingomyelins	30	µM	Sphingomyelin	Biocrates	**0,820**	**0,0235**
Sum sphingomyelins	60	µM	Sphingomyelin	Biocrates	**1,648**	**0,0471**
Sum sphingomyelins	90	µM	Sphingomyelin	Biocrates	**0,752**	**0,0215**
Sum UDCA	0	nM	Bile acid	LC-MS/MS	**0,125**	**0,0036**
Sum UDCA	120	nM	Bile acid	LC-MS/MS	**1,774**	**0,0507**
Sum UDCA	15	nM	Bile acid	LC-MS/MS	**0,013**	**0,0004**
Sum UDCA	240	nM	Bile acid	LC-MS/MS	**1,943**	**0,0556**
Sum UDCA	30	nM	Bile acid	LC-MS/MS	**0,340**	**-0,0097**
Sum UDCA	60	nM	Bile acid	LC-MS/MS	**0,058**	**-0,0017**
Sum UDCA	90	nM	Bile acid	LC-MS/MS	**1,074**	**0,0307**
T4	fasting	pM	Signalling/ hormone	x	**0,244**	**-0,0070**
TAT	fasting	%BW	Body composition	MRI	**0,552**	**0,0158**
TAT	fasting	kg	Body composition	MRI	**0,243**	**-0,0069**
Tau-conj. BA	0	%	Bile acid	LC-MS/MS	**0,017**	**-0,0005**
Tau-conj. BA	120	%	Bile acid	LC-MS/MS	**0,211**	**-0,0060**
Tau-conj. BA	15	%	Bile acid	LC-MS/MS	**0,748**	**-0,0214**
Tau-conj. BA	240	%	Bile acid	LC-MS/MS	**0,206**	**-0,0059**
Tau-conj. BA	30	%	Bile acid	LC-MS/MS	**0,973**	**-0,0278**
Tau-conj. BA	60	%	Bile acid	LC-MS/MS	**0,670**	**-0,0191**
Tau-conj. BA	90	%	Bile acid	LC-MS/MS	**0,441**	**-0,0126**
Tau-conj. BA	0	nM	Bile acid	LC-MS/MS	**0,063**	**0,0018**
Tau-conj. BA	120	nM	Bile acid	LC-MS/MS	**1,011**	**0,0289**
Tau-conj. BA	15	nM	Bile acid	LC-MS/MS	**0,339**	**-0,0097**
Tau-conj. BA	240	nM	Bile acid	LC-MS/MS	**1,148**	**0,0328**
Tau-conj. BA	30	nM	Bile acid	LC-MS/MS	**0,375**	**-0,0107**
Tau-conj. BA	60	nM	Bile acid	LC-MS/MS	**0,055**	**-0,0016**
Tau-conj. BA	90	nM	Bile acid	LC-MS/MS	**0,710**	**0,0203**
Taurine	0	µM	Amino acid metabolism	Biocrates	**0,285**	**-0,0081**
Taurine	120	µM	Amino acid metabolism	Biocrates	**0,405**	**0,0116**
Taurine	15	µM	Amino acid metabolism	Biocrates	**0,795**	**-0,0227**
Taurine	240	µM	Amino acid metabolism	Biocrates	**0,355**	**0,0102**
Taurine	30	µM	Amino acid metabolism	Biocrates	**0,514**	**-0,0147**
Taurine	60	µM	Amino acid metabolism	Biocrates	**0,247**	**0,0071**
Taurine	90	µM	Amino acid metabolism	Biocrates	**0,323**	**0,0092**
TCA	0	nM	Bile acid	LC-MS/MS	**0,415**	**0,0119**
TCA	120	nM	Bile acid	LC-MS/MS	**0,843**	**0,0241**
TCA	15	nM	Bile acid	LC-MS/MS	**0,350**	**-0,0100**
TCA	240	nM	Bile acid	LC-MS/MS	**0,806**	**0,0231**
TCA	30	nM	Bile acid	LC-MS/MS	**0,231**	**0,0066**
TCA	60	nM	Bile acid	LC-MS/MS	**0,365**	**0,0104**
TCA	90	nM	Bile acid	LC-MS/MS	**0,540**	**0,0154**
TCA	0	%	Bile acid	LC-MS/MS	**0,057**	**0,0016**
TCA	120	%	Bile acid	LC-MS/MS	**0,413**	**0,0118**
TCA	15	%	Bile acid	LC-MS/MS	**0,578**	**-0,0165**
TCA	240	%	Bile acid	LC-MS/MS	**0,024**	**0,0007**
TCA	30	%	Bile acid	LC-MS/MS	**0,427**	**-0,0122**
TCA	60	%	Bile acid	LC-MS/MS	**0,203**	**-0,0058**
TCA	90	%	Bile acid	LC-MS/MS	**0,447**	**-0,0128**
TCDCA	0	nM	Bile acid	LC-MS/MS	**0,230**	**-0,0066**
TCDCA	120	nM	Bile acid	LC-MS/MS	**0,836**	**0,0239**
TCDCA	15	nM	Bile acid	LC-MS/MS	**0,011**	**0,0003**
TCDCA	240	nM	Bile acid	LC-MS/MS	**1,054**	**0,0301**
TCDCA	30	nM	Bile acid	LC-MS/MS	**0,376**	**-0,0107**
TCDCA	60	nM	Bile acid	LC-MS/MS	**0,159**	**-0,0045**
TCDCA	90	nM	Bile acid	LC-MS/MS	**0,730**	**0,0209**

Supplemental Table 1. Variables included in the PLS-DA model. Variables are presented with their respective VIP values and loadings, method of detection and units.

TCDCA	0	%	Bile acid	LC-MS/MS	**0,292**	**-0,0084**
TCDCA	120	%	Bile acid	LC-MS/MS	**0,588**	**-0,0168**
TCDCA	15	%	Bile acid	LC-MS/MS	**0,542**	**-0,0155**
TCDCA	240	%	Bile acid	LC-MS/MS	**0,501**	**-0,0143**
TCDCA	30	%	Bile acid	LC-MS/MS	**0,828**	**-0,0237**
TCDCA	60	%	Bile acid	LC-MS/MS	**0,673**	**-0,0193**
TCDCA	90	%	Bile acid	LC-MS/MS	**0,510**	**-0,0146**
TDCA	0	nM	Bile acid	LC-MS/MS	**0,296**	**0,0085**
TDCA	120	nM	Bile acid	LC-MS/MS	**1,208**	**0,0345**
TDCA	15	nM	Bile acid	LC-MS/MS	**0,485**	**-0,0139**
TDCA	240	nM	Bile acid	LC-MS/MS	**1,420**	**0,0406**
TDCA	30	nM	Bile acid	LC-MS/MS	**0,782**	**-0,0224**
TDCA	60	nM	Bile acid	LC-MS/MS	**0,321**	**-0,0092**
TDCA	90	nM	Bile acid	LC-MS/MS	**0,826**	**0,0236**
TDCA	0	%	Bile acid	LC-MS/MS	**0,426**	**0,0122**
TDCA	120	%	Bile acid	LC-MS/MS	**0,005**	**0,0001**
**Primary ID**	**Sampling time**	**Unit**	**Category**	**Method**	**VIP**	**Loadings**
TDCA	15	%	Bile acid	LC-MS/MS	**0,761**	**-0,0218**
TDCA	240	%	Bile acid	LC-MS/MS	**0,277**	**0,0079**
TDCA	30	%	Bile acid	LC-MS/MS	**0,966**	**-0,0276**
TDCA	60	%	Bile acid	LC-MS/MS	**0,518**	**-0,0148**
TDCA	90	%	Bile acid	LC-MS/MS	**0,090**	**0,0026**
Thr	0	µM	Amino acid metabolism	Biocrates	**0,295**	**-0,0084**
Thr	120	µM	Amino acid metabolism	Biocrates	**0,046**	**0,0013**
Thr	15	µM	Amino acid metabolism	Biocrates	**0,302**	**-0,0086**
Thr	240	µM	Amino acid metabolism	Biocrates	**0,025**	**0,0007**
Thr	30	µM	Amino acid metabolism	Biocrates	**0,122**	**0,0035**
Thr	60	µM	Amino acid metabolism	Biocrates	**0,311**	**-0,0089**
Thr	90	µM	Amino acid metabolism	Biocrates	**0,406**	**0,0116**
Threitol	0	AU	Glucose metabolism	GC-MS	**1,126**	**-0,0322**
Threitol	120	AU	Glucose metabolism	GC-MS	**1,525**	**-0,0436**
Threitol	15	AU	Glucose metabolism	GC-MS	**1,330**	**-0,0380**
Threitol	240	AU	Glucose metabolism	GC-MS	**1,397**	**-0,0399**
Threitol	30	AU	Glucose metabolism	GC-MS	**1,026**	**-0,0293**
Threitol	60	AU	Glucose metabolism	GC-MS	**1,595**	**-0,0456**
Threitol	90	AU	Glucose metabolism	GC-MS	**1,347**	**-0,0385**
Tibialis-IMCL	fasting	ratio	Body composition	MRI	**1,243**	**-0,0355**
TLCA	0	nM	Bile acid	LC-MS/MS	**0,435**	**-0,0125**
TLCA	120	nM	Bile acid	LC-MS/MS	**1,164**	**0,0333**
TLCA	15	nM	Bile acid	LC-MS/MS	**0,625**	**0,0179**
TLCA	240	nM	Bile acid	LC-MS/MS	**1,246**	**0,0356**
TLCA	30	nM	Bile acid	LC-MS/MS	**0,258**	**0,0074**
TLCA	60	nM	Bile acid	LC-MS/MS	**0,216**	**0,0062**
TLCA	90	nM	Bile acid	LC-MS/MS	**0,981**	**0,0281**
TLCA	0	%	Bile acid	LC-MS/MS	**0,413**	**0,0118**
TLCA	120	%	Bile acid	LC-MS/MS	**0,212**	**-0,0061**
TLCA	15	%	Bile acid	LC-MS/MS	**0,072**	**-0,0021**
TLCA	240	%	Bile acid	LC-MS/MS	**0,585**	**-0,0167**
TLCA	30	%	Bile acid	LC-MS/MS	**0,155**	**-0,0044**
TLCA	60	%	Bile acid	LC-MS/MS	**0,151**	**-0,0043**
TLCA	90	%	Bile acid	LC-MS/MS	**0,194**	**-0,0055**
TNF-alpha	fasting	pg/ml	Inflammation	ELISA	**0,003**	**-0,0001**
total DMA	0	µM	Biogenic amine	Biocrates	**0,541**	**-0,0155**
total DMA	120	µM	Biogenic amine	Biocrates	**0,142**	**0,0041**
total DMA	15	µM	Biogenic amine	Biocrates	**0,009**	**-0,0003**
total DMA	240	µM	Biogenic amine	Biocrates	**0,386**	**-0,0110**
total DMA	30	µM	Biogenic amine	Biocrates	**0,365**	**-0,0104**
total DMA	60	µM	Biogenic amine	Biocrates	**0,257**	**-0,0074**
total DMA	90	µM	Biogenic amine	Biocrates	**0,301**	**0,0086**
Total thiols	fasting	mM	Oxidative stress	x	**0,653**	**0,0187**
Triglycerides	0	mM	Lipid metabolism	Enzymatic	**2,774**	**-0,0793**
Triglycerides	120	mM	Lipid metabolism	Enzymatic	**2,559**	**-0,0732**
Triglycerides	15	mM	Lipid metabolism	Enzymatic	**2,427**	**-0,0694**
Triglycerides	240	mM	Lipid metabolism	Enzymatic	**2,483**	**-0,0710**
Triglycerides	30	mM	Lipid metabolism	Enzymatic	**2,338**	**-0,0669**
Triglycerides	60	mM	Lipid metabolism	Enzymatic	**2,214**	**-0,0633**
Triglycerides	90	mM	Lipid metabolism	Enzymatic	**2,687**	**-0,0768**
Trp	0	µM	Amino acid metabolism	Biocrates	**1,538**	**-0,0440**
Trp	120	µM	Amino acid metabolism	Biocrates	**1,528**	**-0,0437**
Trp	15	µM	Amino acid metabolism	Biocrates	**1,546**	**-0,0442**
Trp	240	µM	Amino acid metabolism	Biocrates	**1,212**	**-0,0347**

Supplemental Table 1. Variables included in the PLS-DA model. Variables are presented with their respective VIP values and loadings, method of detection and units.

Primary ID	Sampling time	Unit	Category	Method	VIP	Loadings
Trp	30	µM	Amino acid metabolism	Biocrates	**1,136**	**-0,0325**
Trp	60	µM	Amino acid metabolism	Biocrates	**1,004**	**-0,0287**
Trp	90	µM	Amino acid metabolism	Biocrates	**1,895**	**-0,0542**
Trunk:periph. fat	fasting	ratio	Body composition	MRI	**1,178**	**-0,0337**
TSH	fasting	mIU/l	Signalling/ hormone	x	**0,227**	**0,0065**
TUDCA	120	nM	Bile acid	LC-MS/MS	**0,956**	**0,0273**
TUDCA	15	nM	Bile acid	LC-MS/MS	**0,766**	**-0,0219**
TUDCA	240	nM	Bile acid	LC-MS/MS	**1,005**	**0,0288**
TUDCA	30	nM	Bile acid	LC-MS/MS	**0,050**	**0,0014**
TUDCA	60	nM	Bile acid	LC-MS/MS	**0,147**	**-0,0042**
TUDCA	90	nM	Bile acid	LC-MS/MS	**0,484**	**0,0138**
TUDCA	120	%	Bile acid	LC-MS/MS	**1,258**	**-0,0360**
TUDCA	15	%	Bile acid	LC-MS/MS	**0,989**	**-0,0283**
TUDCA	240	%	Bile acid	LC-MS/MS	**1,682**	**-0,0481**
TUDCA	30	%	Bile acid	LC-MS/MS	**1,290**	**-0,0369**
TUDCA	60	%	Bile acid	LC-MS/MS	**1,366**	**-0,0391**
TUDCA	90	%	Bile acid	LC-MS/MS	**1,564**	**-0,0447**
Tyr	0	µM	Amino acid metabolism	Biocrates	**1,416**	**-0,0405**
Tyr	120	µM	Amino acid metabolism	Biocrates	**1,437**	**-0,0411**
Tyr	15	µM	Amino acid metabolism	Biocrates	**1,013**	**-0,0290**
Tyr	240	µM	Amino acid metabolism	Biocrates	**0,746**	**-0,0213**
Tyr	30	µM	Amino acid metabolism	Biocrates	**0,428**	**-0,0122**
Tyr	60	µM	Amino acid metabolism	Biocrates	**0,944**	**-0,0270**
Tyr	90	µM	Amino acid metabolism	Biocrates	**1,371**	**-0,0392**
Urea	0	mM	Amino acid metabolism	Enzymatic	**1,067**	**-0,0305**
Urea	120	mM	Amino acid metabolism	Enzymatic	**1,237**	**-0,0354**
Urea	15	mM	Amino acid metabolism	Enzymatic	**1,123**	**-0,0321**
Urea	240	mM	Amino acid metabolism	Enzymatic	**1,290**	**-0,0369**
Urea	30	mM	Amino acid metabolism	Enzymatic	**1,180**	**-0,0337**
Urea	60	mM	Amino acid metabolism	Enzymatic	**1,359**	**-0,0389**
Urea	90	mM	Amino acid metabolism	Enzymatic	**1,210**	**-0,0346**
Uric acid	fasting	µM	Amino acid metabolism	Enzymatic	**1,194**	**-0,0341**
Val	0	µM	Amino acid metabolism	Biocrates	**1,154**	**-0,0330**
Val	120	µM	Amino acid metabolism	Biocrates	**1,590**	**-0,0455**
Val	15	µM	Amino acid metabolism	Biocrates	**1,194**	**-0,0341**
Val	240	µM	Amino acid metabolism	Biocrates	**1,112**	**-0,0318**
Val	30	µM	Amino acid metabolism	Biocrates	**0,739**	**-0,0211**
Val	60	µM	Amino acid metabolism	Biocrates	**1,216**	**-0,0348**
Val	90	µM	Amino acid metabolism	Biocrates	**1,559**	**-0,0446**
VCAM	fasting	ng/ml	Inflammation	ELISA	**0,744**	**-0,0213**
Waist	fasting	cm	Body composition	Measuring tape	**1,373**	**-0,0393**
White blood cell	0	giga/l	Leucocyte	Cell counter	**1,716**	**-0,0491**
White blood cell	120	giga/l	Leucocyte	Cell counter	**1,401**	**-0,0401**
White blood cell	15	giga/l	Leucocyte	Cell counter	**1,859**	**-0,0532**
White blood cell	240	giga/l	Leucocyte	Cell counter	**2,174**	**-0,0622**
White blood cell	30	giga/l	Leucocyte	Cell counter	**1,750**	**-0,0501**
White blood cell	60	giga/l	Leucocyte	Cell counter	**1,495**	**-0,0428**
White blood cell	90	giga/l	Leucocyte	Cell counter	**1,260**	**-0,0360**
